# The effect of exposure to long working hours on ischaemic heart disease: A systematic review and meta-analysis from the WHO/ILO Joint Estimates of the Work-related Burden of Disease and Injury

**DOI:** 10.1016/j.envint.2020.105739

**Published:** 2020-09

**Authors:** Jian Li, Frank Pega, Yuka Ujita, Chantal Brisson, Els Clays, Alexis Descatha, Marco M. Ferrario, Lode Godderis, Sergio Iavicoli, Paul A. Landsbergis, Maria-Inti Metzendorf, Rebecca L. Morgan, Daniela V. Pachito, Hynek Pikhart, Bernd Richter, Mattia Roncaioli, Reiner Rugulies, Peter L. Schnall, Grace Sembajwe, Xavier Trudel, Akizumi Tsutsumi, Tracey J. Woodruff, Johannes Siegrist

**Affiliations:** a Department of Environmental Health Sciences, Fielding School of Public Health, School of Nursing, University of California, Los Angeles, United States; b Environment, Climate Change and Health Department, World Health Organization, 20 Avenue Appia, 1211 Geneva 27, Switzerland; c Labour Administration, Labour Inspection and Occupational Safety and Health Branch, International Labour Organization, Route des Morillons 4, 1211 Geneva, Switzerland; d Centre de Recherche du CHU de Québec, Université Laval, 1050 Chemin Ste-Foy, Quebec City G1S 4L8, Quebec, Canada; e Department of Public Health and Primary Care, Ghent University, Campus University Hospital Ghent (4K3 – entrance 42), 4K3, Corneel Heymanslaan 10, B-9000 Ghent, Belgium; f AP-HP (Paris Hospital), Occupational Health Unit, Poincaré University Hospital, Garches, France; g Inserm Versailles St-Quentin Univ – Paris Saclay Univ (UVSQ), UMS 011, UMR-S 1168, Villejuif, France; h Univ Angers, CHU Angers, Univ Rennes, Inserm, EHESP, Irset (Institut de recherche en santé, environnement et travail) – UMR_S 1085, F-49000 Angers, France; i Research Centre EPIMED, University of Insubria, Via O Rossi 9, 21100 Varese, Italy; j Centre for Environment and Health, KU Leuven, Leuven, Belgium; k KIR Department (Knowledge, Information & Research), IDEWE, External Service for Prevention and Protection at Work, Leuven, Belgium; l Inail, Department of Occupational and Environmental Medicine, Epidemiology and Hygiene, Via Fontana Candida 1, 00078 Monte Porzio Catone (Rome), Italy; m SUNY-Downstate Health Sciences University, School of Public Health, 450 Clarkson Ave., Brooklyn, NY 11238, United States; n Cochrane Metabolic and Endocrine Disorders Group, Institute of General Practice, Medical Faculty of the Heinrich-Heine-University, Düsseldorf, Germany; o Department of Health Research Methods, Evidence, and Impact, McMaster University, Health Sciences Centre, Hamilton, Canada; p Hospital Sírio-Libanês and Disciplina de Economia e Gestão em Saúde, Universidade Federal de São Paulo, 412 Barata Ribeiro, Sao Paulo, Brazil; q Institute of Epidemiology and Health Care, University College London, 1-19 Torrington Place, London WC1E 6BT, United Kingdom; r National Research Centre for the Working Environment, Lersø Parkallé 105, DK-2100 Copenhagen, Denmark; s Department of Public Health, University of Copenhagen, Øster Farimagsgade 5, DK-1014 Copenhagen, Denmark; t Department of Psychology, University of Copenhagen, Øster Farimagsgade 2A, DK-1353 Copenhagen, Denmark; u Center for Occupational and Environmental Health, University of California-Irvine, 100 Theory Way, Irvine, CA, United States; v Department of Occupational Medicine, Epidemiology and Prevention (OMEP), Donald and Barbara Zucker School of Medicine at Hofstra University, 175 Community Drive, NY 11021, United States; w Department of Environmental, Occupational, and Geospatial Health Sciences, CUNY Graduate School of Public Health and Health Policy, 55 W 125th Street, New York, NY 10027, United States; x Department of Public Health, School of Medicine, Kitasato University, 1-15-1 Kitasato, Minami, Sagamihara 252-0374, Japan; y Program on Reproductive Health and the Environment, University of California San Francisco, San Francisco, United States; z Life Science Centre, University of Düsseldorf, Merowingerplatz 1a, Düsseldorf 40225, Germany

**Keywords:** Long working hours, Ischaemic heart disease, Systematic review, Meta-analysis

## Abstract

**Background:**

The World Health Organization (WHO) and the International Labour Organization (ILO) are developing Joint Estimates of the work-related burden of disease and injury (WHO/ILO Joint Estimates), with contributions from a large network of experts. Evidence from mechanistic data suggests that exposure to long working hours may cause ischaemic heart disease (IHD). In this paper, we present a systematic review and meta-analysis of parameters for estimating the number of deaths and disability-adjusted life years from IHD that are attributable to exposure to long working hours, for the development of the WHO/ILO Joint Estimates.

**Objectives:**

We aimed to systematically review and meta-analyse estimates of the effect of exposure to long working hours (three categories: 41–48, 49–54 and ≥55 h/week), compared with exposure to standard working hours (35–40 h/week), on IHD (three outcomes: prevalence, incidence and mortality).

**Data sources:**

We developed and published a protocol, applying the Navigation Guide as an organizing systematic review framework where feasible. We searched electronic databases for potentially relevant records from published and unpublished studies, including MEDLINE, Scopus, Web of Science, CISDOC, PsycINFO, and WHO ICTRP. We also searched grey literature databases, Internet search engines and organizational websites; hand-searched reference lists of previous systematic reviews; and consulted additional experts.

**Study eligibility and criteria:**

We included working-age (≥15 years) workers in the formal and informal economy in any WHO and/or ILO Member State but excluded children (aged < 15 years) and unpaid domestic workers. We included randomized controlled trials, cohort studies, case-control studies and other non-randomized intervention studies which contained an estimate of the effect of exposure to long working hours (41–48, 49–54 and ≥55 h/week), compared with exposure to standard working hours (35–40 h/week), on IHD (prevalence, incidence or mortality).

**Study appraisal and synthesis methods:**

At least two review authors independently screened titles and abstracts against the eligibility criteria at a first stage and full texts of potentially eligible records at a second stage, followed by extraction of data from qualifying studies. Missing data were requested from principal study authors. We combined relative risks using random-effect meta-analysis. Two or more review authors assessed the risk of bias, quality of evidence and strength of evidence, using Navigation Guide and GRADE tools and approaches adapted to this project.

**Results:**

Thirty-seven studies (26 prospective cohort studies and 11 case-control studies) met the inclusion criteria, comprising a total of 768,751 participants (310,954 females) in 13 countries in three WHO regions (Americas, Europe and Western Pacific). The exposure was measured using self-reports in all studies, and the outcome was assessed with administrative health records (30 studies) or self-reported physician diagnosis (7 studies). The outcome was defined as incident non-fatal IHD event in 19 studies (8 cohort studies, 11 case-control studies), incident fatal IHD event in two studies (both cohort studies), and incident non-fatal or fatal (“mixed”) event in 16 studies (all cohort studies). Because we judged cohort studies to have a relatively lower risk of bias, we prioritized evidence from these studies and treated evidence from case-control studies as supporting evidence. For the bodies of evidence for both outcomes with any eligible studies (i.e. IHD incidence and mortality), we did not have serious concerns for risk of bias (at least for the cohort studies).

No eligible study was found on the effect of long working hours on IHD prevalence. Compared with working 35–40 h/week, we are uncertain about the effect on acquiring (or incidence of) IHD of working 41–48 h/week (relative risk (RR) 0.98, 95% confidence interval (CI) 0.91 to 1.07, 20 studies, 312,209 participants, I^2^ 0%, low quality of evidence) and 49–54 h/week (RR 1.05, 95% CI 0.94 to 1.17, 18 studies, 308,405 participants, I^2^ 0%, low quality of evidence). Compared with working 35–40 h/week, working ≥55 h/week may have led to a moderately, clinically meaningful increase in the risk of acquiring IHD, when followed up between one year and 20 years (RR 1.13, 95% CI 1.02 to 1.26, 22 studies, 339,680 participants, I^2^ 5%, moderate quality of evidence).

Compared with working 35–40 h/week, we are very uncertain about the effect on dying (mortality) from IHD of working 41–48 h/week (RR 0.99, 95% CI 0.88 to 1.12, 13 studies, 288,278 participants, I^2^ 8%, low quality of evidence) and 49–54 h/week (RR 1.01, 95% CI 0.82 to 1.25, 11 studies, 284,474 participants, I^2^ 13%, low quality of evidence). Compared with working 35–40 h/week, working ≥55 h/week may have led to a moderate, clinically meaningful increase in the risk of dying from IHD when followed up between eight and 30 years (RR 1.17, 95% CI 1.05 to 1.31, 16 studies, 726,803 participants, I^2^ 0%, moderate quality of evidence).

Subgroup analyses found no evidence for differences by WHO region and sex, but RRs were higher among persons with lower SES. Sensitivity analyses found no differences by outcome definition (exclusively non-fatal or fatal versus “mixed”), outcome measurement (health records versus self-reports) and risk of bias (“high”/“probably high” ratings in any domain versus “low”/“probably low” in all domains).

**Conclusions:**

We judged the existing bodies of evidence for human evidence as “inadequate evidence for harmfulness” for the exposure categories 41–48 and 49–54 h/week for IHD prevalence, incidence and mortality, and for the exposure category ≥55 h/week for IHD prevalence. Evidence on exposure to working ≥55 h/week was judged as “sufficient evidence of harmfulness” for IHD incidence and mortality. Producing estimates for the burden of IHD attributable to exposure to working ≥55 h/week appears evidence-based, and the pooled effect estimates presented in this systematic review could be used as input data for the WHO/ILO Joint Estimates.

## 1 Introduction background

The World Health Organization (WHO) and the International Labour Organization (ILO) are finalizing Joint Estimates of the work-related burden of disease and injury (WHO/ILO Joint Estimates) (Ryder, 2017). The organizations are estimating the numbers of deaths and disability-adjusted life years (DALYs) that are attributable to selected occupational risk factors. The WHO/ILO Joint Estimates are based on already existing WHO and ILO methodologies for estimating the burden of disease for selected occupational risk factors (Ezzati et al., 2004, Internation Labour Organization, 1999, Internation Labour Organization, 2014, Pruss-Ustun et al., 2017). They expand these existing methodologies with estimation of the burden of several prioritized additional pairs of occupational risk factors and health outcomes. For this purpose, population attributable fractions (Murray et al., 2004) are being calculated for each additional risk factor-outcome pair, and these fractions are being applied to the total disease burden envelopes for the health outcome from the WHO *Global Health Estimates* for the years 2000–2016 (World Health Organization, 2020). Population attributable fractions are the proportional reduction in burden from the health outcome achieved by a reduction of exposure to the risk factor to zero.

The WHO/ILO Joint Estimates may include estimates of the burden of ischaemic heart disease (IHD) attributable to exposure to long working hours, if feasible, as one additional risk factor-outcome pair whose global burden of disease has not previously been estimated. To select parameters with the best and least biased evidence for our estimation models, we conducted a systematic review and meta-analysis of studies on the relationship between exposure to long working hours and IHD. We present our findings in the current paper. WHO and ILO, supported by a large network of experts, are in parallel also producing a systematic review of studies estimating the prevalence of exposure to long working hours (Li et al., 2018). The review of prevalence of exposure is applying novel systematic review methods (e.g., the RoB-SPEO risk of bias tool (Pega et al., 2020). The organizations are also in parallel conducting several other systematic reviews and meta-analyses on other additional risk factor-outcome pairs (Descatha et al., 2018, Godderis et al., 2018, Hulshof et al., 2019, Li et al., 2018, Mandrioli et al., 2018, Paulo et al., 2019, Rugulies et al., 2019, Teixeira et al., 2019, Tenkate et al., 2019). To our knowledge, these are the first systematic reviews and meta-analyses (with a pre-published protocol) conducted specifically for an occupational burden of disease study (Mandrioli et al., 2018). The WHO’s and ILO’s joint estimation methodology and the WHO/ILO Joint Estimates are separate from these systematic reviews, and they will be described in more detail and reported elsewhere.

### 1.1 Rationale

To consider the feasibility of estimating the burden of IHD attributable to exposure to long working hours, and to ensure that potential estimates of burden of IHD are reported in adherence with the *Guidelines for Accurate and Transparent Health Estimates Reporting* (GATHER) (Stevens et al., 2016), WHO and ILO require a systematic review of studies on the prevalence of relevant levels of exposure to long working hours (forthcoming). The WHO and ILO also require a systematic review and meta-analysis with estimates of the relative effect of exposure to long work hours on IHD prevalence, incidence and mortality, compared with the theoretical minimum risk exposure level (the systematic review presented here). The theoretical minimum risk exposure level is the exposure level that would result in the lowest possible population risk, even if it is not feasible to attain this exposure level in practice (Murray et al., 2004). These data and effect estimates should be tailored to serve as parameters for estimating the burden of IHD from exposure to long work hours in the WHO/ILO Joint Estimates.

We are aware of at least five prior systematic reviews on the effect of long working hours on IHD published since 2012. First, a 2012 systematic review and meta-analysis on the effect of exposure to long working hours on cardiovascular disease, which included five cohort studies and six case-control studies published up to September 2011, reported a pooled odds ratio (OR) of 1.37 (95% confidence interval (CI) 1.11–1.70) (Kang et al., 2012). Second, a 2012 systematic review on the effect of long working hours on IHD included four prospective cohort studies and seven case-control studies published between 1966 and 19 January 2011 and reported pooled relative risks (RRs) of 1.39 (95% CI 1.12–1.72) for the prospective cohort studies and 2.43 (95% CI 1.81–3.26) for the case-control studies, respectively. Third, a 2015 systematic review, individual-participant data analysis and meta-analysis of 25 cohort studies (including 20 unpublished studies) in countries in the WHO regions of the Americas, Europe and the Western Pacific up to 20 August 2014 found a relative risk (RR) of 1.13 (95% CI 1.02–1.26; 22 cohort studies) for the effect of long working hours (≥55 h/week) on IHD (Kivimaki et al., 2015). Fourth, a 2018 update of the Kivimaki et al., 2015 systematic review added one additional cohort study (i.e., the Danish Labour Force Survey) and found that exposure to working ≥55 h/week led to an increase in risk of IHD by an estimated 12% (95% CI 1.03–1.21; 23 cohort studies) (Virtanen and Kivimaki, 2018). Both the Kivimaki et al., 2015 systematic review and its 2018 update combined in meta-analyses studies with non-fatal, fatal and fatal or non-fatal (“mixed”) IHD events. However, burden of disease estimation requires evidence separately on IHD incidence (ideally non-fatal events only) and mortality (ideally fatal events only). Fifth, a 2019 meta-analysis included cross-sectional studies, case-control studies, and prospective cohort studies published between 1998 and 2018, and it reported that long working hours were associated with cardiovascular heart diseases (pooled OR 1.54, 95% CI 1.32–1.79) (Wong et al., 2019). In summary, all previous systematic reviews and meta-analyses consistently concluded that working long hours increases the risk of IHD. To our knowledge, none of the prior systematic reviews had a pre-published protocol and/or missed other essential aspects of a systematic review. Our systematic review is fully compliant with latest systematic review methods (including use of a protocol) and expands the scope of the existing systematic review evidence by covering evidence from studies published up to 27 August 2019.

Our systematic review covers workers in the formal and in the informal economy. The informal economy is defined as “all economic activities by workers and economic units that are – in law or in practice – not covered or insufficiently covered by formal arrangements” (104th International Labour Conference, 2015). It does not comprise “illicit activities, in particular the provision of services or the production, sale, possession or use of goods forbidden by law, including the illicit production and trafficking of drugs, the illicit manufacturing of and trafficking in firearms, trafficking in persons and money laundering, as defined in the relevant international treaties” (104th International Labour Conference, 2015). Work in the informal economy may lead to different exposures and exposure effects than does work in the formal economy. Consequently, formality of work (informal vs. formal) may be an effect modifier of the effect of long working hours on IHD. Therefore, we consider in the systematic review the formality of the economy reported in included studies.

### 1.2 Description of the risk factor

Burden of disease estimation requires unambiguous definition of the risk factor, risk factor levels and the theoretical minimum risk exposure level. Long working hours are defined as working hours exceeding standard working hours, i.e. any working hours of ≥41 h/week (Table 1). Based on results from earlier studies on long working hours and health endpoints (e.g., (Kivimaki et al., 2015, Virtanen et al., 2015)), the preferred four exposure level categories for our systematic review are 35–40, 41–48, 49–54 and ≥55  h/week (Table 1).

**Table 1:** Definitions of the risk factor, risk factor levels and the minimum risk exposure level.

	Definition
Risk factor	Long working hours (including those spent in secondary jobs), defined as working hours >40 h/week, i.e. working hours exceeding standard working hours (35–40 h/week).
Risk factor levels	Four levels: 1. 35–40 h/week. 2. 41–48 h/week. 3. 49–54 h/week. 4. ≥55 h/week.
Theoretical minimum risk exposure level	Standard working hours, defined as working hours of 35–40 h/week.

The theoretical minimum risk exposure is standard working hours defined as 35–40  h/week (Table 1). We acknowledge that it is possible that the theoretical minimum risk exposure might be lower than standard working hours, but working hours ≤35 h/week had to be excluded because studies indicate that some persons working less than standard hours do so because of existing health problems (Kivimaki et al., 2015, Virtanen et al., 2012). In other words, persons working less than standard hours might belong to a health-selected group or a group concerned with family care and therefore cannot serve as comparators. Consequently, if a study used as the reference group persons working less than standard hours or a combination of persons working standard hours and persons working less than standard hours, it would be excluded from the systematic review and meta-analysis. The category 35–40  h/week is the reference group used in many large studies and previous systematic reviews (Kang et al., 2012, Virtanen et al., 2012).

### 1.3 Definition of the outcome

The *WHO Global Health Estimates* group outcomes into standard burden of disease categories (World Health Organization, 2017), based on standard codes from the *International Statistical Classification of Diseases and Related Health Problems 10th Revision* (ICD-10) (World Health Organization, 2015). The relevant *WHO Global Health Estimates* category for this systematic review is “II.H.2 Ischaemic heart disease” (World Health Organization, 2017). In line with the *WHO Global Health Estimates*, we define the health outcome covered in this systematic review as IHD, defined as conditions with ICD-10 codes I20 to I25 (Table 2). Table 2 shows that this review covers all the relevant categories of diseases or health problems included in the *WHO Global Health Estimates* category.

**Table 2:** ICD-10 codes and disease and health problems covered by the WHO Global Health Estimates categories “II.H.2 Ischaemic heart disease” and their inclusion in the systematic review.

ICD-10 code	Disease or health problem	Included in this review
I20	Angina pectoris	Yes
I21	Acute myocardial infarction	Yes
I22	Subsequent myocardial infarction	Yes
I23	Certain current complications following acute myocardial infarction	Yes
I24	Other acute ischaemic heart diseases	Yes
I25	Chronic ischaemic heart disease	Yes

### 1.4 How the risk factor may impact the outcome

**Fig. 1:**
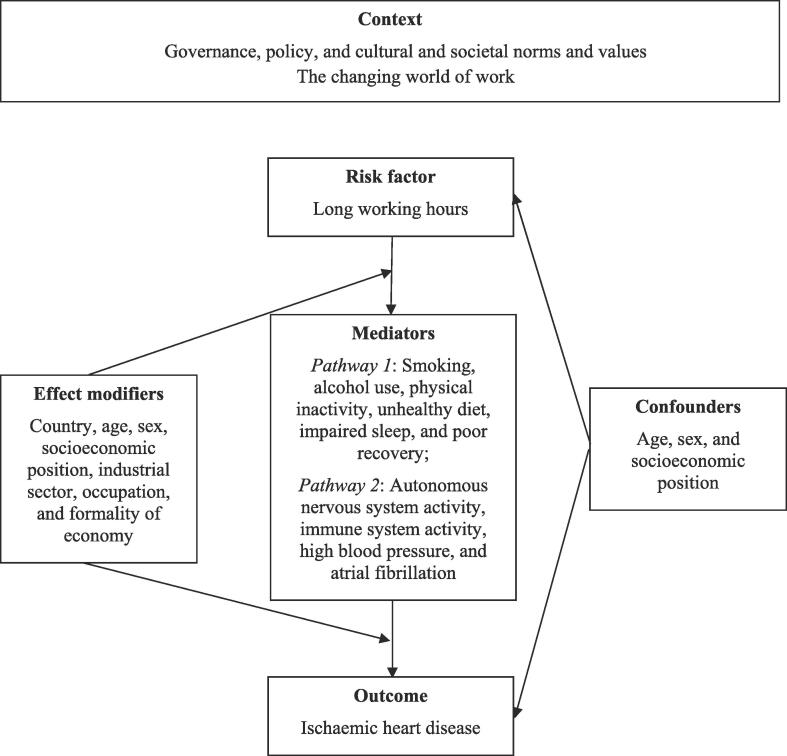
Logic model of the possible causal relationship between exposure to long working hours and ischaemic heart disease.

Fig. 1 presents the logic model for our systematic review of the causal relationship between exposure to long working hours and IHD, taken from our protocol (Li et al., 2018). This logic model is an a priori, process-orientated one (Rehfuess et al., 2018) that seeks to capture the complexity of the risk factor–outcome causal relationship (Anderson et al., 2011).

Theoretically, distinct social contexts in the world of work are likely to exacerbate or mitigate the effect of exposure to long working hours on IHD risk. While empirical tests of this assumption are not available, these contexts can exert a direct effect on working hours. Evidence suggests that economic globalization drives people around the world to work longer hours (Lee et al., 2007).

Based on knowledge of previous research on long working hours and IHD (Kivimaki et al., 2015, Virtanen et al., 2015), we assume that the effect of exposure to long working hours on IHD could be modified by country (or WHO region), sex, age, industrial sector, occupation, socioeconomic position and formality of the economy. Three important individual-level variables, age, sex and socioeconomic position (usually assessed by income, education or occupational grade) have been included in several previous studies as confounding factors, given the probability of differential exposure and effect modification by these variables. Up to now, there is no empirical evidence on potential interactions between them, nor do we know to what extent they do modify the effect of long working hours on IHD (an example would be that the effect is stronger among participants with low socioeconomic position due to their increased general susceptibility). These three variables were analyzed in this systematic review, whenever available. Exceptions are accepted for studies whose study samples were homogenous (such as men only) or that conducted sensitivity analyses to test the presence of confounding (such as sex-disaggregated analyses that can help identify confounding by sex).

Several variables may mediate the effects of this exposure on disease risk through two major pathways. The first one concerns behavioural responses that result in an increase in health-adverse behaviours, such as cigarette smoking, high alcohol consumption, unhealthy diet and physical inactivity. These behaviours are established risk factors of IHD (Taris et al., 2011, Virtanen et al., 2015). Moreover, impaired sleep and poor recovery resulting from this exposure increase the risk of IHD (Sonnentag et al., 2017, Virtanen et al., 2009). Chronic psychosocial stress responses define a second pathway mediating the effects of exposure on IHD. According to established physiological evidence, recurrent high effort (exposure) results in continued activation of the autonomic nervous/immune systems and associated stress axes, the sympatho-adrenal medullary and the hypothalamic-pituitary adrenal axes, with excessive release of respective stress hormones (i.e., adrenalin, noradrenalin and cortisol) (Chandola et al., 2010, Jarczok et al., 2013, Nakata, 2012). In the longer run, this recurrent activation exceeds the regulatory capacity of the cardiovascular system, thus triggering functional dysregulations (e.g., disturbed sympatho-vagal balance resulting in low heart rate variability, sustained high blood pressure) and structural lesions (e.g., atherogenesis in coronary vessels) (Kivimaki and Steptoe, 2018). Importantly, some experiments representing psychosocial stress at work (e.g., high work pressure, unfair pay) demonstrated direct effects on indicators of cardiovascular dysregulation (e.g., reduced heart rate variability; Dulleck et al., 2014, Falk et al., 2018). Extended exposure to these conditions, as is the case with long working hours, promotes cardiovascular disease susceptibility by the processes identified in the ‘allostatic load’ model (McEwen, 1998).

In addition to epidemiological, clinical and experimental evidence suggesting that chronic psychosocial stress (including that from working long hours) presents a risk factor of IHD, there is indirect evidence on its causal role from animal studies. In classical experiments with cynomolgus macaques, a direct effect of exposure to a chronic psychosocial stressor on growth of atherosclerotic plaques in coronary vessels was demonstrated, and this process was prevented by administration of beta-adrenergic blocking agents (Kaplan and Manuck, 1994).

## 2 Objectives

To systematically review and meta-analyse evidence on the effect of exposure to long working hours (three categories: 41–48, 49–54 and ≥55 h/week) on IHD prevalence, incidence and mortality among workers of working age, compared with the minimum risk exposure level (standard working hours: 35–40 h/week).

## 3 Methods

### 3.1 Developed protocol

We applied the Navigation Guide (Woodruff and Sutton, 2014) methodology for systematic reviews in environmental and occupational health as our guiding methodological framework, wherever feasible. The guide applies established systematic review methods from clinical medicine, including standard Cochrane methods for systematic reviews of interventions, to the field of environmental and occupational health. The methods ensure systematic and rigorous evidence synthesis on environmental and occupational risk factors that reduces bias and maximizes transparency (Woodruff and Sutton, 2014). The need for further methodological development and refinement of the relatively novel Navigation Guide has been acknowledged (Woodruff and Sutton, 2014). From the perspective of the Navigation Guide framework, all steps were conducted (i.e., steps 1–6 in Fig. 1 in (Woodruff and Sutton, 2014) for the stream on human data and none of the steps for the stream on non-human data, although we narratively synthesized the mechanistic evidence from non-human data that we were aware of (Section 1.4).

We registered the protocol in PROSPERO under CRD42017084243. The protocol adheres to the preferred reporting items for systematic review and meta-analysis protocols statement (PRISMA-P) (Moher et al., 2015, Shamseer et al., 2015), with the abstract adhering to the reporting items for systematic reviews in journal and conference abstracts (PRISMA-A) (Beller et al., 2013). Any modification of the methods stated in the protocol was registered in PROSPERO and reported in the systematic review itself (Section 8). Our systematic review is reported according to the preferred reporting items for systematic review and meta-analysis statement (PRISMA) (Liberati et al., 2009). Our reporting of the parameters for estimating the burden of IHD to long working hours in the systematic review adheres to the requirements of the GATHER guidelines (Stevens et al., 2016). This is done because the WHO/ILO burden of disease estimates that may be produced following the systematic review must also adhere to these reporting guidelines.

### 3.2 Searched literature

#### 3.2.1 Electronic databases

We searched the six following electronic databases:

1. WHO International Clinical Trials Registry Platform (to 6 July 2018).
2. Ovid MEDLINE(R) Epub Ahead of Print, In-Process & Other Non-Indexed Citations, Ovid MEDLINE(R) Daily and Ovid MEDLINE(R) 1946 to Present (1 January 1946 to 27 August 2019).
3. Scopus (1 January 1995 to 6 July 2018).
4. Web of Science (1 January 1900 to 6 July 2018).
5. CISDOC archived database (1901–2012 searched on 6 July 2018).
6. PsycINFO Ovid (1 January 1880 to 6 July 2018)

The Ovid MEDLINE search strategy was presented in the protocol (Li et al., 2018). The full search strategies for all databases were revised by an information scientist and are presented in Appendix 3 in the Supplementary data. Searches were performed in electronic databases operated in the English language using a search strategy in the English language. When we neared completion of the review, we conducted a top-up search of the MEDLINE database on 27th August 2019 to capture the most recent publications (e.g., publications ahead of print). Deviations from the proposed search strategy and the actual search strategy are documented in Section 8.

#### 3.2.2 Grey literature databases

We also searched the two following two grey literature databases in July 2018;

OpenGrey (http://www.opengrey.eu/)
Grey Literature Report (http://greylit.org/)

We used the following search strategy: ((“work hours” OR “working hours” OR “long work” OR “long working” OR “long hours” OR overtime OR overwork OR workload OR employee*) AND (myocardial OR heart OR coronary OR cardiovascular OR angina)).

#### 3.2.3 Internet search engines

We also searched the Google (www.google.com/) and Google Scholar (www.google.com/scholar/) Internet search engines and screened the first 100 hits for potentially relevant records, as was previously done (Pega et al., 2015, Pega et al., 2017).

#### 3.2.4 Organizational websites

The websites of the seven following international organizations and national government departments were searched on the 15th September 2018 using the keywords “myocardial”, “coronary”, “cardiovascular”, “heart”:

1. International Labour Organization (www.ilo.org/).
2. World Health Organization (www.who.int).
3. European Agency for Safety and Health at Work (https://osha.europa.eu/en).
4. Eurostat (http://www.ec.europa.eu/eurostat/web/main/home).
5. China National Knowledge Infrastructure (http://www.cnki.net/).
6. Finnish Institute of Occupational Health (https://www.ttl.fi/en/).
7. United States National Institute for Occupational Safety and Health (NIOSH) of the United States of America, using the NIOSH data and statistics gateway (https://www.cdc.gov/niosh/data/).

#### 3.2.5 Hand-searching and expert consultation

We hand-searched for potentially eligible studies in:

• Reference lists of previous systematic reviews.
• Reference lists of all included trials register records.
• Study protocols published over the past 24 months in the three peer-reviewed academic journals with the largest number of included studies.
• Published study protocols that have cited the included studies (identified in Web of Science citation database).
• Collections of the review authors.

Additional experts were contacted with a list of included studies, with the request to identify potentially eligible additional studies.

### 3.3 Selected studies

Study selection was carried out with the Covidence software (Veritas Health Innovation). All records identified in the search were downloaded and duplicates were identified and deleted. Afterwards, two review authors independently and in duplicate screened titles and abstracts (step 1) and then full texts (step 2) of potentially relevant records. A third review author resolved any disagreements between the two review authors. If a study record identified in the literature search was authored by a review author assigned to study selection or if an assigned review author was involved in the study, the record was re-assigned to another review author for study selection. The study selection is presented in a flow chart, as per PRISMA guidelines (Liberati et al., 2009).

### 3.4 Eligibility criteria

The PECO (Morgan et al., 2018) criteria are described below.

#### 3.4.1 Types of populations

We included studies of the working-age population (≥15 years) in the formal and informal economy. Studies of children (aged < 15 years) and unpaid domestic workers were excluded. Participants residing in any WHO and/or ILO Member State and any industrial setting or occupational group were included. Exposure to long working hours may potentially have further population reach (e.g., across generations for workers of reproductive age) and acknowledged that the scope of our systematic review does not capture these populations and impacts on them. Our protocol paper (in Appendix F) provides a complete, but briefer overview of the PECO criteria (Li et al., 2018).

#### 3.4.2 Types of exposures

We included studies that defined long working hours in accordance with our standard definition (Table 1). We again prioritized measures of the total number of hours worked, including in both of: main and secondary jobs, self-employment and salaried employment, whether in the informal or the formal economy. We included studies with objective (e.g., by means of time recording technology) or subjective measurements of long working hours, whether, including studies that used measurements by experts (e.g., scientists with subject matter expertise) and self-reports by the worker, workplace administrator or manager. If a study presented both objective and subjective measurements, then we prioritized objective ones. Studies with measures from any data source, including registry data, were included.

For studies that reported exposure levels differing from our standard levels (Table 1), we converted the reported levels to the standard levels if possible, and reported analyses on these alternate exposure levels if impossible.

#### 3.4.3 Types of comparators

The included comparator were participants exposed to the theoretical minimum risk exposure level: worked 35–40 h/week (Table 1). We excluded all other comparators.

#### 3.4.4 Types of outcomes

This systematic review included three outcomes:

1. Has IHD (or, in other words, IHD prevalence).
2. Acquired IHD (IHD incidence).
3. Died from IHD (IHD mortality).

We included studies that define IHD in accordance with our standard definition (Table 2). Other coronary-related unspecific symptoms (e.g., chest pain) were excluded. We did, however, include the outcome definition of IHD via a proxy term of heart trouble, given the fact that approximately 80% of heart disease is IHD, while at the same time acknowledging that heart trouble includes other small portion of heart conditions such as hypertensive heart disease, rheumatic heart disease, and inflammatory heart disease (Mendis et al., 2011). This outcome definition has also been included in previous systematic reviews and meta-analyses of the effect of working long hours on IHD, including the (Kivimaki et al., 2015) one. We expected that most studies examining exposure to long working hours and its effect on IHD have documented ICD-10 diagnostic codes. In the remaining cases, methods that approximate ICD-10 criteria ascertained the outcome, such as physician-diagnosed self-reports (see also Appendix 4 in the supplementary data and Section 5.3. *Limitations of this systematic review*).

The following measurements of IHD are regarded as eligible:

i. Diagnosis by a physician with imaging.
ii. Hospital discharge records.
iii. Other relevant administrative data (e.g., records of sickness absence or disability).
iv. Medically certified cause of death.

All other measures were excluded from this systematic review.

Objective (e.g., health records) and subjective (e.g., self-reports) measures of the outcome are eligible. If a study presents both objective and subjective measurements, then the objective ones were prioritized.

Studies with “mixed” outcome definitions (i.e., including both fatal IHD events and non-fatal IHD events) provide evidence on both the outcome IHD incidence and the outcome IHD mortality. These studies were consequently included in analyses on both of these outcomes, as long as they were sufficiently heterogeneous statistically with studies of non-fatal events only and fatal events only, respectively (as determinded by sensitivity analyses; Section 3.9).

#### 3.4.5 Types of studies

We included studies that investigated the effect of long working hours on IHD for any years. Eligible study designs were randomized controlled trials (including parallel-group, cluster, cross-over and factorial trials), cohort studies (both prospective and retrospective), case-control studies, and other non-randomized intervention studies (including quasi-randomized controlled trials, controlled before-after studies and interrupted time series studies). We included a broader set of observational study designs than is commonly included, because a recent augmented Cochrane Review of complex interventions identified valuable additional studies using such a broader set of designs (Arditi et al., 2016). As we have an interest in quantifying risk and not in qualitative assessment of hazard (Barroga and Kojima, 2013), all other study designs were excluded (e.g., uncontrolled before-and-after, cross-sectional, qualitative, modelling, case and non-original studies).

Records published in any year and any language were included. Again, the search was conducted using English language terms, so that records published in any language that present essential information (i.e. title and abstract) in English were included. If a record was written in a language other than those spoken by the authors of this review or those of other reviews (Descatha et al., 2018, Godderis et al., 2018, Hulshof et al., 2019, Mandrioli et al., 2018, Paulo et al., 2019, Rugulies et al., 2019, Teixeira et al., 2019, Tenkate et al., 2019) in the series (i.e. Arabic, Bulgarian, Chinese, Danish, Dutch, English, French, Finnish, German, Hungarian, Italian, Japanese, Norwegian, Portuguese, Russian, Spanish, Swedish and Thai), then the record was translated into English. Published and unpublished studies were included. Studies conducted using unethical practices were excluded (e.g., studies that deliberately exposed humans to a known risk factor to human health).

#### 3.4.6 Types of effect measures

We included measures of the relative effect of a relevant level of long working hours on the risk of IHD (prevalence, incidence and mortality), compared with the theoretical minimum risk exposure level. We included relative effect measures such as RRs, ORs and hazard ratios for both incidence measures and mortality measures (e.g., developed or died from IHD). Measures of absolute effects (e.g., mean differences in risks or odds) were converted into relative effect measures, but if conversion was impossible, they were excluded. To ensure comparability of effect estimates and to facilitate meta-analysis, if a study presented an OR, then it was converted into an RR, if possible, using the guidance provided in the Cochrane handbook for systematic reviews of interventions (Higgins and Green, 2011).

If a study presented estimates for the effect from two or more alternative models that had been adjusted for different variables, then we systematically prioritized the estimate from the model that provided information on the relevant confounders or mediators, at least the core variables defined in Fig. 1: age, sex, and socioeconomic position. We prioritized estimates from models adjusted for more potential confounders over those from models adjusted for fewer. For example, if a study presents estimates from a crude, unadjusted model (Model A), a model adjusted for one potential confounder (e.g., age; Model B) and a model adjusted for two potential confounders (e.g., age and sex; Model C), then we prioritized the estimate from Model C. We prioritized estimates from models unadjusted for mediators over those from models that adjusted for mediators, because adjustment for mediators can introduce bias. For example, if Model A has been adjusted for two confounders, and Model B has been adjusted for the same two confounders and a potential mediator, then we chose the estimate from Model A over that from Model B. We prioritized estimates from models that can adjust for time-varying confounders that are at the same time also mediators, such as marginal structural models (Pega et al., 2016), over estimates from models that can only adjust for time-varying confounders, such as fixed-effects models (Gunasekara et al., 2014). Similarly, we prioritized estimates from models that adjust for time-varying confounders over models that do not adjust for time-varying confounding. If a study presents effect estimates from two or more potentially eligible models, then we documented why we prioritized the selected model.

### 3.5 Extracted data

A standard data extraction form was developed and trialled until data extractors reached convergence and agreement. At least two review authors independently extracted data on study characteristics (including study authors, study year, study country, participants, exposure and outcome), study design (including study type, comparator, epidemiological model(s) used and effect estimate measure) and risk of bias (including source population representation, blinding, exposure assessment, outcome assessment, confounding, incomplete outcome data, selective outcome reporting, conflict of interest and other sources of bias). A third review author resolved conflicts in data extraction. Data were entered into and managed with Excel.

We also extracted data on potential conflict of interest in included studies. For each author and affiliated organization of each included study record, their financial disclosures and funding sources were extracted. We used a modification of a previous method to identify and assess undisclosed financial interest of authors (Forsyth et al., 2014). Where no financial disclosure or conflict of interest statements were available, the names of all authors were searched in other study records gathered for this study and published in the prior 36 months and in other publicly available declarations of interests (Drazen et al., 2010a, Drazen et al., 2010b).

### 3.6 Requested missing data

We requested missing data from the principal study author by email or phone, using the contact details provided in the principal study record. If we did not receive a positive response from the study author, follow-up emails were sent twice, at two and four weeks. We present a description of missing data, the study author from whom the data were requested, the date of requests sent, the date on which data were received (if any), and a summary of the responses provided by the study authors (Appendix 1 in the Supplementary data). If we did not receive some or all of the requested missing data, we nevertheless retained the study in the systematic review as long as it fulfilled our eligibility criteria.

### 3.7 Assessed risk of bias

Standard risk of bias tools do not exist for systematic reviews for hazard identification or those for risk assessment in occupational and environmental health. The five such tools developed specifically for occupational and environmental health are for either or both hazard identification and risk assessment, and they differ substantially in the types of studies (randomized, observational and/or simulation studies) and data (e.g., human, animal and/or *in vitro*) they seek to assess (Rooney et al., 2016). However, all five tools, including the Navigation Guide one (Lam et al., 2016c), assess risk of bias in human studies similarly (Rooney et al., 2016).

Consistent with using the Navigation Guide as our organizing framework, we used its risk of bias tool, which builds on the standard risk of bias assessment methods of Cochrane (Higgins et al., 2011) and the US Agency for Healthcare Research and Quality (Viswanathan et al., 2008). Some further refinements of the Navigation Guide method may be warranted (Goodman et al., 2017), but it has been successfully applied in several completed and ongoing systematic reviews (Johnson et al., 2016, Johnson et al., 2014, Koustas et al., 2014, Lam et al., 2016a, Lam et al., 2014, Lam et al., 2017;125., Lam et al., 2016b, Vesterinen et al., 2014). In our application of the Navigation Guide method, we draw heavily on one of its latest versions, as presented in the protocol for an ongoing systematic review (Lam et al., 2016c).

Risk of bias was assessed on the individual study level and across the body of evidence for each outcome. The nine risk of bias domains included in the Navigation Guide method for human data were: (i) source population representation; (ii) blinding; (iii) exposure assessment; (iv) outcome assessment; (v) confounding; (vi) incomplete outcome data; (vii) selective outcome reporting; (viii) conflict of interest; and (ix) other sources of bias. Risk of bias or confounding ratings for all domains were: “low”; “probably low”; “probably high”; “high” or “not applicable” (Lam et al., 2016c). To judge the risk of bias in each domain, we applied a priori instructions (Li et al., 2018), which were adapted from an ongoing Navigation Guide systematic review (Lam et al., 2016c), and further described in our protocol (Li et al., 2018). For example, a study was assessed as carrying “low” risk of bias from source population representation, if we judge the source population to be described in sufficient detail (including eligibility criteria, recruitment, enrolment, participation and loss to follow up) and the distribution and characteristics of the study sample to indicate minimal or no risk of selection effects.

All risk of bias assessors jointly trialled the application of the risk of bias criteria until they had synchronized their understanding and application of these criteria. Two or more study authors independently judged (or assessed) the risk of bias for each study by outcome. Where individual assessments differed, a third author resolved the conflict. For each included study, we reported our risk of bias assessment at the level of the individual study by domain in a standard ‘Risk of bias table’ (Higgins et al., 2011). For the entire body of evidence, we presented the study-level risk of bias ratings by domains in a ‘Risk of bias summary figure’ (or ‘Risk of bias matrix’) (Higgins et al., 2011).

### 3.8 Synthesised evidence (including conducted meta-analysis)

We conducted separate meta-analyses for all outcomes: Has IHD, Acquired IHD, and Died from IHD. Studies of different designs were not combined quantitatively. If we found two or more studies with an eligible effect estimate, two or more review authors independently investigated the clinical heterogeneity (Deeks et al., 2011) of the studies in terms of participants (including country, sex, age and industrial sector or occupation), level of risk factor exposure, comparator and outcomes, following our protocol (Li et al., 2018). If we found that effect estimates differed considerably by WHO region, sex and/or age, or a combination of these, then evidence was synthesized for the relevant populations defined by WHO region, sex and/or age, or combination thereof. If we found effect estimates to be clinically homogenous across WHO regions, sex and age groups, then we combined studies from all of these populations into one pooled effect estimate that can be applied across all combinations of WHO regions, sexes and age groups in the WHO/ILO Joint Estimates.

If we judged two or more studies for the relevant combination of WHO region, sex and age group, or combination thereof, to be sufficiently clinically homogenous to potentially be combined quantitatively using quantitative meta-analysis, then the statistical heterogeneity of the studies was tested using the I^2^ statistic (Figueroa, 2014). If two or more clinically homogenous studies were found to be sufficiently homogenous statistically to be combined in a meta-analysis, we pooled the RRs of the studies in a quantitative meta-analysis, using the inverse variance method with a random effects model to account for cross-study heterogeneity (Figueroa, 2014). The meta-analysis was conducted in RevMan 5.3.

We neither quantitatively combined data from studies with different designs (e.g., did not combine cohort studies with case-control studies), nor did we combine unadjusted with adjusted models. We only combined studies that we judged to have a minimum acceptable level of adjustment for the three core confounders identified (Fig. 1, Section 3.4.5).

If we found studies with “pure” outcome definitions (i.e. capturing exclusively either non-fatal or fatal IHD events) and “mixed” outcome definitions (i.e. capturing any IHD events, whether non-fatal or fatal), then we conducted “exploratory subgroup analyses” in which we sub-grouped studies by “pure” versus “mixed” outcome definitions. Before conducting these analyses, we formulated the following rules for determining inclusion of these studies in quantitative meta-analyses:

• If there was no evidence for (meaningful) subgroup differences, then we would pool studies with “mixed” and “pure” outcome definitions.
• If there was evidence for (meaningful) subgroup differences, then we would not pool studies with “mixed” and “pure” outcome definitions.

If quantitative synthesis was not feasible (for instance, due to different exposure levels as defined above), the study findings were synthesized narratively and we identified the estimates that we judged to be the highest quality evidence available.

### 3.9 Conducted subgroup and sensitivity analyses

Subgroup analyses were conducted only for the main meta-analysis and comparison of interest (i.e., the meta-analysis of cohort studies for the comparison of worked ≥55 h/week versus worked 35–40 h/week). We conducted subgroup analyses by:

• WHO region.
• Sex.
• Age group.
• SES.

We also planned to conduct subgroup analyses by occupation, industrial sector and formality of economy, but did not find evidence or receive missing data to populate these subgroup analyses.

We conducted the following sensitivity analyses:

• Studies with exclusively non-fatal or fatal IHD events, compared with studies with “mixed” (non-fatal and/or fatal) IHD events.
• Studies judged to be of “high”/“probably high” risk of bias in any domain, compared with “low”/“probably low” risk of bias in all domains.
• Studies with documented or approximated ICD-10 diagnostic codes (e.g., as recorded in administrative health records), compared with studies without ICD-10 diagnostic codes (e.g., self-reports).

We planned to also compare studies with “low” or “probably low” risk of bias from conflict of interest with studies with any “high” or “probably high” risk of bias in this domain. However, we did not conduct such sensitivity analyses because we rated no included study to have any such risk of bias from conflict of interest.

### 3.10 Assessed quality of evidence

We assessed quality of evidence using a modified version of the Navigation Guide quality of evidence assessment tool (Lam et al., 2016c). The tool is based on the Grading of Recommendations Assessment, Development and Evaluation (GRADE) approach (Schünemann et al., 2011) adapted specifically to systematic reviews in occupational and environmental health (Morgan et al., 2016).

At least two review authors assessed quality of evidence for the entire body of evidence by outcome, with any disagreements resolved by a third review author. We adapted the latest Navigation Guide instructions (Lam et al., 2016c) for grading the quality of evidence and presented the adapted instructions in our protocol (Li et al., 2018). We downgraded the quality of evidence for the following five reasons: (i) risk of bias; (ii) inconsistency; (iii) indirectness; (iv) imprecision; and (v) publication bias (Balshem et al., 2011). These items were considered downgrades if they could not be explained. If our systematic review had included ten or more studies, we aimed to generate an Egger’s funnel plot to judge concerns on publication bias. If it included nine or fewer studies, we judged the risk of publication bias qualitatively.

We graded the quality of the entire body of evidence by outcome, using the three Navigation Guide standard quality of evidence ratings: “high”, “moderate” and “low” (Lam et al., 2016c). Within each of the relevant domains, the concern was rated for the quality of evidence, using the ratings “none”, “serious” and “very serious”. As per Navigation Guide, we started at “high” for randomized studies and “moderate” for observational studies. Quality was downgraded if there was no concern by nil grades (0), for a serious concern by one grade (-1) and for a very serious concern by two grades (-2). We upgraded the quality of evidence for the following other reasons: large effect, dose–response and plausible residual confounding and bias. There had to be compelling reasons to upgrade or downgrade. If we had a serious concern for risk of bias in a body of evidence consisting of observational studies (-1), but had no other concerns, and had no reasons for upgrading, then we downgraded the quality of evidence by one grade from “moderate” to “low”.

### 3.11 Assessed strength of evidence

Our systematic review included observational epidemiologic studies of human data only, and no other streams of evidence (e.g., no studies of non-human data). The standard Navigation Guide methodology (Lam et al., 2016c) allows for rating human and non-human animal studies separately, and then combining the strength of evidence for each stream for an overall strength of evidence rating. However, the Navigation Guide also allows for rating one stream of evidence based on the factors described above (i.e., risk of bias, indirectness, inconsistency, imprecisions, publication bias, large magnitude of effect, dose–response and residual confounding) to arrive at an overall rating of the quality of evidence as ‘high’, ‘moderate’ or ‘low’ (see above and the protocol). The approach of evaluating only the human evidence stream is consistent with the GRADE methodology that has adopted the Bradford Hill considerations (Schunemann et al., 2011). So, using the method above based on the Navigation Guide incorporates the considerations of Bradford Hill (Table 3).

**Table 3:** Bradford Hill considerations and their relationship to GRADE and the Navigation Guide for evaluating the overall quality of the evidence for human observational studies.

Bradford Hill	GRADE	Navigation Guide
Strength	Strength of association and imprecision in effect estimate	Strength of association and imprecision in effect estimate
Consistency	Consistency across studies, i.e., across different situations (different researchers)	Consistency across studies, i.e., across different situations (different researchers)
Temporality	Study design, properly designed and conducted observational studies	Study design, properly designed and conducted observational studies
Biological Gradient	Dose response gradient	Dose response gradient
Specificity	Indirectness	Indirectness
Coherence	Indirectness	Indirectness
Experiment	Study design, properly designed and conducted observational studies	Study design, properly designed and conducted observational studies
Analogy	Existing association for critical outcomes leads to not downgrading the quality, indirectness	Existing association for critical outcomes leads to not downgrading the quality, indirectness. Evaluating the overall strength of body of human evidence allows consideration of other compelling attributes of the data that may influence certainty.

Table adapted from (Schunemann et al., 2011).

There is an additional step that is described in the protocol that integrates the quality of the evidence (method for assessing described above) with other elements including direction of effect and confidence in the effect and other compelling attributes of the data. These attributes may influence our certainty to allow for an overall rating that consists of “sufficient evidence of toxicity/harmfulness”, “limited of toxicity/harmfulness”, “inadequate of toxicity/harmfulness” and “evidence of lack of toxicity/harmfulness” based on human evidence. This approach to evaluate only the human evidence has been applied in previous systematic reviews (Lam et al., 2016c, Lam et al., 2017;125.) and verified by the US National Academy of Sciences (National Academies of Sciences, 2017). It also provides two steps that integrate Bradford Hill criteria (evaluating the quality of the evidence and then evaluating the overall strength of evidence). Finally, the GRADE quality of evidence ratings (which are the same as for Navigation Guide) are analogous to the final ratings from Bradford Hill for causality which has been described in Schunemann et al., 2011 (Table 4).

**Table 4:** Interpretation of the GRADE ratings of the overall quality of evidence and the Navigation Guide ratings for strength of evidence evaluation.

GRADE rating for quality of evidence	Interpretation of GRADE rating	Navigation Guide rating for strength of evidence for human evidence	Interpretation of Navigation Guide rating
High	There is high confidence that the true effect lies close to that of the estimate of the effect.	Sufficient evidence of toxicity	A positive relationship is observed between exposure and outcome where chance, bias, and confounding can be ruled out with reasonable confidence. The available evidence includes results from one or more well-designed, well conducted studies, and the conclusion is unlikely to be strongly affected by the results of future studies.
Moderate	There is moderate confidence in the effect estimate: the true effect is likely to be close to the estimate of the effect, but there is a possibility that it is substantially different.	Limited evidence of toxicity	A positive relationship is observed between exposure and outcome where chance, bias, and confounding cannot be ruled out with reasonable confidence. Confidence in the relationship is constrained by such factors as: the number, size, or quality of individual studies, or inconsistency of findings across individual studies. As more information becomes available, the observed effect could change, and this change may be large enough to alter the conclusion.
Low	The panel’s confidence in the effect estimate is limited: the true effect may be substantially different from the estimate of the effect	Inadequate evidence of toxicity	The available evidence is insufficient to assess effects of the exposure. Evidence is insufficient because of: the limited number or size of studies, low quality of individual studies, or inconsistency of findings across individual studies. More information may allow an assessment of effects.
Very Low	There is little confidence in the effect estimate: the true effect is likely to be substantially different from the estimate of effect.		

Adapted from (Schunemann et al., 2011) and (Lam et al., 2016c).

## 4 Results

### 4.1 Study selection

**Fig. 2:**
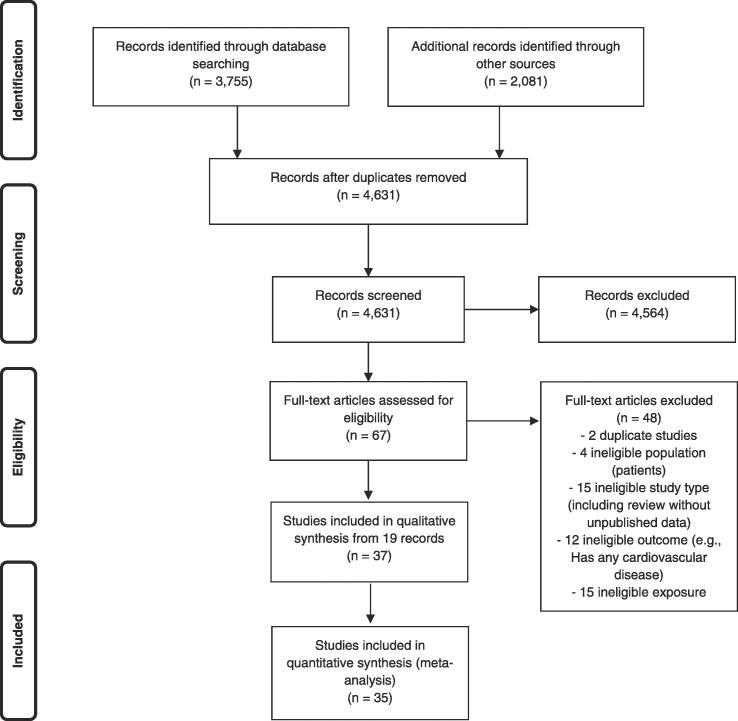
Flow diagram of study selection.

Of the total of 4,631 individual study records identified in our searches, 19 records reporting results from 37 studies fulfilled the eligibility criteria and were included in the systematic review (Fig. 2). For the 45 excluded studies that most closely resembled inclusion criteria, the reasons for exclusion are listed in Appendix 2 in the Supplementary data. Of the 37 included studies, 35 studies were included in one or more quantitative meta-analyses (Fig. 2).

### 4.2 Characteristics of included studies

The characteristics of the included studies are summarized in Table 5.

**Table 5:** Characteristics of included studies in the systematic review of long working hours and ischaemic heart disease.

(Part I: study population and study type)										
Study	Study population							Study type		
Study ID	Total number of study participants	Number of female study participants	Country of study population	Geographic location (specify as 'national' or list regions or sites)	Industrial sector (specify ISIC-4 code provided in worksheet “Industrial sector codes”)	Occupation (specify ISCO-08 code provided in worksheet “Occupation code”)	Age	Study design	Study period (month of first collection of any data and month of last collection of any data)	Follow-up period (period in months between exposure and outcome)
Virtanen 2012 - Russek 1958	200 (100 cases and 100 controls)	3 or more unclear	United States	Not reported	Not reported	Not reported	20–45 years	Case-control study	1948–1958	N/A
Virtanen 2012 - Theorell and Rahe (1972)	171 (62 cases and 109 controls)	0	Sweden	Local	Not reported	Not reported	Mean age: cases 56 years, controls 53 years	Case-control study	January 1966 to January 1970	N/A
Virtanen 2012 - Thiel et al. (1973)	100 (50 MI case and 50 control)	0	United States	Local	Not reported	Not reported	40–60 years	Case-control study	Unclear	N/A
Virtanen 2012 - Falger 1992	458 (133 cases, 133 neighbourhood controls and 192 hospital controls)	0	Netherlands	Region	Not reported	Not reported	Mean age: cases 53.1 years, neighbourhood controls 49.2 years, hospital controls 51.1 years	Case-control study	Unclear	N/A
Virtanen 2012 - Sokejima and Kagamimori (1998)	526 (195 cases, 331 controls)	0	Japan	Not reported	Not reported	Not reported	Mean age: cases 55.5 years, controls 54.4 years	Case-control study	November 1990 to November 1993	N/A
Virtanen 2012 - Liu and Tanaka (2002)	705 (260 cases and 445 controls)	0	Japan	Local	Not reported	Not reported	40–79 years	Case-control study	September 1996 to March 1999	N/A
Virtanen 2012 - Fukuoka 2005	94 (47 cases; 47 controls)	2	Japan	Region	Not reported	Not reported	Mean age: cases 52 years, controls 50 years	Case-control study	2002	N/A
Jeong 2013	868 (99 cases; 769 controls)	149 (male 82.8%)	Korea, Republic of	Not reported	Not reported	Not reported	Mean age: cases 49.5 years, controls 49.8 years	Case-control study	November 2009 to October 2011	N/A
Cheng et al. (2014)	966 (322 cases and 644 controls)	0	China (Taiwan)	Region	Not reported	Not reported	cases: 26–64 years controls: 27–64 years	Case-control study	January 2008 - November 2011 (cases). September 2007 (controls)	N/A
Ma et al. (2017)	595 (354 cases and 241 controls)	189	China	Local	Not reported	Not reported	Mean age: cases 55.28 years controls 51.94 years	Case-control study	December 2015 to November 2016	N/A
McGwin 2005	692 (116 cases and 576 controls)	215	United States	National	Not reported	Not reported	Mean age: cases 53.7 years controls 53.8 years	Case-control study	1996 to 2000	N/A
Kivimaki 2015 - Virtanen et al. (2010)	6014	1752	United Kingdom	Local	84 Public administration and defence; compulsory social security	Not reported	Mean age 48.7 years at baseline	Prospective cohort study	1991–1994 (baseline) to 2002–2004	11 years
Kivimaki 2015 - Netterstrøm et al. (2010)	1146	595	Denmark	Local	Not reported	Not reported	Mean age 47.0 years at baseline	Prospective cohort study	January 1993-June 1994 (baseline) to July 2007	13 to 14.5 years
Kivimaki 2015 - Holtermann 2010	4943	0	Denmark	Region	Not reported	Not reported	Mean age 48.6 years at baseline	Prospective cohort study	1970–1971 (baseline) to end of 2001	30 years
Kivimaki 2015 - Toker et al. (2012)	8838	3126	Israel	Local	Not reported	Not reported	Mean age 44.9 years at baseline	Prospective cohort study	2003–2008 (baseline) to December 2010	Averagely 3.6 years (12 months to 8 years)
Kivimaki 2015 - O'Reilly 2013	414,949	144,938	United Kingdom	Region	Not reported	Not reported	Mean age 39.0 years at baseline	Prospective cohort study	2001 (baseline) to 2009	8.7 years
Kivimaki 2015 - WOLF-S 1992	5554	2395	Sweden	Region	Not reported	Not reported	Mean age 41.5 years at baseline	Prospective cohort study	1992–1995 (baseline)	unclear
Kivimaki 2015 - Belstress 1994	11,685	0	Belgium	National	Not reported	Not reported	Mean age 45.8 years at baseline	Prospective cohort study	1994–1998 (baseline)	unclear
Kivimaki 2015 - WOLF-N 1996	4648	772	Sweden	Region	Not reported	Not reported	Mean age 44.0 years at baseline	Prospective cohort study	1996–1998 (baseline)	unclear
Kivimaki 2015 - COPSOQ-I 1997	1803	876	Denmark	National	Not reported	Not reported	Mean age 40.6 years at baseline	Prospective cohort study	1997 (baseline)	unclear
Kivimaki 2015 - HeSSup 1998	16,150	8971	Finland	National	Not reported	Not reported	Mean age 39.6 years at baseline	Prospective cohort study	1998 (baseline)	unclear
Kivimaki 2015 - FPS 2000	44,565	35,840	Finland	National	Not reported	Not reported	Mean age 44.6 years at baseline	Prospective cohort study	2000 (baseline)	unclear
Kivimaki 2015 - HNR 2000	1774	732	Germany	Region	Not reported	Not reported	Mean age 53.3 years at baseline	Prospective cohort study	2000 (baseline)	unclear
Kivimaki 2015 - DWECS 2000	5535	2590	Denmark	National	Not reported	Not reported	Mean age 41.8 years at baseline	Prospective cohort study	2000 (baseline)	unclear
Kivimaki 2015 - COPSOQ-II 2004	3389	1785	Denmark	National	Not reported	Not reported	Mean age 42.7 years at baseline	Prospective cohort study	2004 (baseline)	unclear
Kivimaki 2015 - IPAW 1996	2021	1360	Denmark	Region	Not reported	Not reported	Mean age 41.2 years at baseline	Prospective cohort study	1996–1997 (baseline)	unclear
Kivimaki 2015 - PUMA 1999	1783	1473	Denmark	Region	Not reported	Not reported	Mean age 42.7 years at baseline	Prospective cohort study	1999 (baseline)	unclear
Kivimaki 2015 - NWCS 2005	43,510	22,178	Netherlands	National	Not reported	Not reported	Mean age 40.1 years at baseline	Prospective cohort study	2005–2006 (baseline)	unclear
Kivimaki 2015 - Alameda 1973	1585	666	United States	Region	Not reported	Not reported	Mean age 44.4 years at baseline	Prospective cohort study	1973 (baseline) to 1994	20 years
Kivimaki 2015 - NHANES I 1982	4875	2800	United States	National	Not reported	Not reported	Mean age 48.8 years at baseline	Prospective cohort study	1982 (baseline) to 1992	10 years
Kivimaki 2015 - ACL 1986	1502	802	United States	National	Not reported	Not reported	Mean age 44.5 years at baseline	Prospective cohort study	1986 (baseline) to 2002	16 years
Kivimaki 2015 - WLSG 1992	5421	2883	United States	Region	Not reported	Not reported	Mean age 54.1 years at baseline	Prospective cohort study	1992 (baseline) to 2003–2005	10 years
Kivimaki 2015 - WLSS 1993	2366	1299	United States	Region	Not reported	Not reported	Mean age 52.4 years at baseline	Prospective cohort study	1993 (baseline) to 2004–2007	12 years
Kivimaki 2015 - MIDUS 1995	3303	1637	United States	National	Not reported	Not reported	Mean age 44.2 years at baseline	Prospective cohort study	1995 (baseline) to 2005	10 years
Kivimaki 2015 - HILDA 2003	4879	2343	Australia	National	Not reported	Not reported	Mean age 41.4 years at baseline	Prospective cohort study	2003 (baseline) to 2007	4 years
Hannerz et al. (2018)	145,861	68,583	Denmark	National	Not reported	Not reported	15–75 years at baseline	Prospective cohort study	1999 (baseline) to 2013	average 7.7 years
Hayashi 2019	15,277	0	Japan	region	Not reported	Not reported	40–59 years at baseline (means 48.1 to 51.2)	Prospective cohort study	1993 (baseline) to2012	20 years

*Part II: exposure assessment and comparator*								

Study	Exposure assessment							Comparator

Study ID	Exposure definition (i.e., how was the exposure defined?)	Unit for which exposure was assessed	Mode of exposure data collection	Exposure assessment methods	Levels/intensity of exposure (specify unit)	Number of study participants in exposed group	Number of study participants in unexposed group	Definition of comparator (define comparator group, including specific level of exposure)

Virtanen 2012 - Russek 1958	engaged jobs other than main occupation or working 60 h or more per week	Individual level	Face-to-face survey	Self-report	50 and below hours/week, 51 and more hours/week	Not reported	Not reported	Non-occupational stress OR worked<60 h per week OR worked<60 h per week and non-contemporary day and evening work.
Virtanen 2012 - Theorell and Rahe (1972)	overtime work more than the Swedish standard	Individual level	Pen-and-paper survey	Self-report	overtime work	Not reported	Not reported	non-overtime work
Virtanen 2012 - Thiel et al. (1973)	average working hours per week	Individual level	Face-to-face survey	Self-report	50 and below hours/week, 51 and more hours/week	Not reported	Not reported	50 and below hours/week
Virtanen 2012 - Falger 1992	prolonged overtime	Individual level	Face-to-face survey	Self-report	overtime work	Not reported	Not reported	no overtime work
Virtanen 2012 - Sokejima and Kagamimori (1998)	Mean daily working hours≥11.01	Individual level	Pen-and-paper survey	Self-report	≤7h/d, 7.01–9h/d, 9.01–11*h*/d, ≥11*h*/d	Not reported	Not reported	7.01–9h/d
Virtanen 2012 - Liu and Tanaka (2002)	Weekly working hours>61 was defined as overtime work.	Individual level	Face-to-face survey	Self-report	≤ 40 h/week, 41–60 h/week, ≥61 h/week	Not reported	Not reported	≤ 40 h/week
Virtanen 2012 - Fukuoka 2005	working hours per week	Individual level	Face-to-face survey	Self-report	≤ 40 h/week, 41–49 h/week, 50–54 h/week, ≥55 h/week	Not reported	Not reported	≤ 40 h/week
Jeong 2013	working hours per week	Individual level	Unclear	Self-report	≤ 40 h/week, 40.1–48 h/week, 49–52 h/week, > 52 h/week	Not reported	Not reported	≤ 40 h/week
Cheng et al. (2014)	working hours duration per day during the week prior to their disease onset (case) the same information during the week prior to the survey (control)	Individual level	Unclear	Self-report	<40 h/week, 40–48 h/week, 49–60 h/week, > 60 h/week	Not reported	Not reported	40 to ≤48 h/week
Ma et al. (2017)	Working hours per week	Individual level	Face-to-face survey	Self-report	none, <35 h/week, 35–40 h/week, 41–48 h/week, 49–54 h/week, and 55 h/week	Not reported	Not reported	Worktime: none
McGwin 2005	Average hours worked per week	Individual level	Computer-administered survey	Self-report	< 40 h/week, = 40 h/week, 41–50 h/week, > 50 h/week	Not reported	Not reported	< 40 h/week
Kivimaki 2015 - Virtanen et al. (2010)	overtime work per day	Individual level	Pen-and-paper survey	Self-report	No overtime, daily 1h overtime, 2h overtime, 3-4h overtime)	2758	3256	no overtime work (7–8 working h/d)
Kivimaki 2015 - Netterstrøm et al. (2010)	Weekly working hours	Individual level	Pen-and-paper survey	Self-report	<30 h/week, 30–37 h/week, 38–49 h/week, ≥50 h/week	1034	112	< 30 h/week
Kivimaki 2015 - Holtermann 2010	Weekly work hours	Individual level	Pen-and-paper survey	Self-report	≤ 40 h/week, 41–45 h/week, ≥46 h/week	4326	638	≤ 40 h/week
Kivimaki 2015 - Toker et al. (2012)	number of hours of work per day	Individual level	Pen-and-paper survey	Self-report	Continuous variable	Not reported	Not reported	7–8 h/week
Kivimaki 2015 - O'Reilly 2013	hours per week (55 and over)	Individual level	Pen-and-paper survey	Self-report	35–40 h/week, 41–48 h/week, 49–54 h/week, ≥55 h/week	39,069	375,880	35–40 h/week
Kivimaki 2015 - WOLF-S 1992	weekly working hours	Individual level	Unclear	Self-report	35–40 h/week, 41–48 h/week, 49–54 h/week, ≥55 h/week	232	5322	35–40 h/week
Kivimaki 2015 - Belstress 1994	weekly working hours	Individual level	Unclear	Self-report	35–40 h/week, 41–48 h/week, 49–54 h/week, ≥55 h/week	723	10,962	35–40 h/week
Kivimaki 2015 - WOLF-N 1996	weekly working hours	Individual level	Unclear	Self-report	35–40 h/week, 41–48 h/week, 49–54 h/week, ≥55 h/week	55	4593	35–40 h/week
Kivimaki 2015 - COPSOQ-I 1997	weekly working hours	Individual level	Unclear	Self-report	35–40 h/week, 41–48 h/week, 49–54 h/week, ≥55 h/week	109	1694	35–40 h/week
Kivimaki 2015 - HeSSup 1998	weekly working hours	Individual level	Unclear	Self-report	35–40 h/week, 41–48 h/week, 49–54 h/week, ≥55 h/week	1417	14,733	35–40 h/week
Kivimaki 2015 - FPS 2000	weekly working hours	Individual level	Unclear	Self-report	35–40 h/week, 41–48 h/week, 49–54 h/week, ≥55 h/week	1414	43,151	35–40 h/week
Kivimaki 2015 - HNR 2000	weekly working hours	Individual level	Unclear	Self-report	35–40 h/week, 41–48 h/week, 49–54 h/week, ≥55 h/week	295	1479	35–40 h/week
Kivimaki 2015 - DWECS 2000	weekly working hours	Individual level	Unclear	Self-report	35–40 h/week, 41–48 h/week, 49–54 h/week, ≥55 h/week	440	5095	35–40 h/week
Kivimaki 2015 - COPSOQ-II 2004	weekly working hours	Individual level	Unclear	Self-report	35–40 h/week, 41–48 h/week, 49–54 h/week, ≥55 h/week	177	3212	35–40 h/week
Kivimaki 2015 - IPAW 1996	weekly working hours	Individual level	Unclear	Self-report	35–40 h/week, 41–48 h/week, 49–54 h/week, ≥55 h/week	6	2015	35–40 h/week
Kivimaki 2015 - PUMA 1999	weekly working hours	Individual level	Unclear	Self-report	35–40 h/week, 41–48 h/week, 49–54 h/week, ≥55 h/week	17	1766	35–40 h/week
Kivimaki 2015 - NWCS 2005	weekly working hours	Individual level	Unclear	Self-report	35–40 h/week, 41–48 h/week, 49–54 h/week, ≥55 h/week	2893	40,617	35–40 h/week
Kivimaki 2015 - Alameda 1973	weekly working hours	Individual level	Unclear	Self-report	35–40 h/week, 41–48 h/week, 49–54 h/week, ≥55 h/week	152	1433	35–40 h/week
Kivimaki 2015 - NHANES I 1982	weekly working hours	Individual level	Unclear	Self-report	35–40 h/week, 41–48 h/week, 49–54 h/week, ≥55 h/week	477	4398	35–40 h/week
Kivimaki 2015 - ACL 1986	weekly working hours	Individual level	Unclear	Self-report	35–40 h/week, 41–48 h/week, 49–54 h/week, ≥55 h/week	181	1321	35–40 h/week
Kivimaki 2015 - WLSG 1992	weekly working hours	Individual level	Unclear	Self-report	35–40 h/week, 41–48 h/week, 49–54 h/week, ≥55 h/week	724	4697	35–40 h/week
Kivimaki 2015 - WLSS 1993	weekly working hours	Individual level	Unclear	Self-report	35–40 h/week, 41–48 h/week, 49–54 h/week, ≥55 h/week	324	2042	35–40 h/week
Kivimaki 2015 - MIDUS 1995	weekly working hours	Individual level	Unclear	Self-report	35–40 h/week, 41–48 h/week, 49–54 h/week, ≥55 h/week	464	2893	35–40 h/week
Kivimaki 2015 - HILDA 2003	weekly working hours	Individual level	Unclear	Self-report	35–40 h/week, 41–48 h/week, 49–54 h/week, ≥55 h/week	541	4338	35–40 h/week
Hannerz et al. (2018)	Weekly working hours	Individual level	Face-to-face survey	Self-report	35–40 h/week, 41–48 h/week, 49–54 h/week, ≥55 h/week	23,143	112,593	35–40 h/week
Hayashi 2019	Working hours by day	Individual level	survey	Self-report	<7h/d; 7 to <9h/d; 9 to <11*h*/d; ≥11*h*/d	7987	6596	7 to <9h/d

Part III: outcome assessment and statistical modelling													

Study	Outcome assessment							Statistical modelling					

Study ID	Definition of outcome	Which International Classification of Diseases (ICD) code was reported for the outcome (if any)?	Diagnostic assessment method	Number of cases with outcome of interest in exposed group	Number of non-cases (i.e. without outcome of interest) in exposed group	Number of cases with outcome of interest in unexposed group	Number of non-cases (i.e. without outcome of interest) in unexposed group	Adjusted for confounding by: age	Adjusted for confounding by: sex	Adjusted for confounding by: SES	Other potential confounders adjusted for	Adjusted for any mediation	Treatment effect measure type

Virtanen 2012 - Russek 1958	Coronary Heart Disease (myocardial infarction or typical angina of effort)	Not reported	Physician diagnostic record	N/A because of study design	N/A because of study design	N/A because of study design	N/A because of study design	No	Yes	Yes	Unclear	No	OR
Virtanen 2012 - Theorell and Rahe (1972)	Non-fatal myocardial infarction	Not reported	Hospital discharge record	N/A because of study design	N/A because of study design	N/A because of study design	N/A because of study design	No	No	No	Unclear	No	OR
Virtanen 2012 - Thiel et al. (1973)	Acute myocardial infarction	Not reported	Physician diagnostic record	N/A because of study design	N/A because of study design	N/A because of study design	N/A because of study design	No	No	Unclear	Unclear	No	OR
Virtanen 2012 - Falger 1992	Acute myocardial infarction	Not reported	Hospital admission record	N/A because of study design	N/A because of study design	N/A because of study design	N/A because of study design	Yes	No	Yes	Yes	No	OR
Virtanen 2012 - Sokejima and Kagamimori (1998)	Acute myocardial infarction	Not reported	Physician diagnostic record	N/A because of study design	N/A because of study design	N/A because of study design	N/A because of study design	Yes	No	Yes	Yes	No	OR
Virtanen 2012 - Liu and Tanaka (2002)	Non-fatal acute myocardial infarction	Not reported	Hospital admission record	N/A because of study design	N/A because of study design	N/A because of study design	N/A because of study design	Yes	No	Unclear	Yes	No	OR
Virtanen 2012 - Fukuoka 2005	Acute myocardial infarction	Not reported	Hospital admission record	N/A because of study design	N/A because of study design	N/A because of study design	N/A because of study design	Yes	No	No	Unclear	Unclear	OR
Jeong 2013	Non-fatal acute myocardial infarction	ICD-10 121	Hospital admission record	N/A because of study design	N/A because of study design	N/A because of study design	N/A because of study design	Yes	Yes	Yes	Yes	No	OR
Cheng et al. (2014)	Acute myocardial infarction or severe coronary heart disease	Not reported	Hospital admission record	N/A because of study design	N/A because of study design	N/A because of study design	N/A because of study design	Yes	No	Yes	Yes	No	OR
Ma et al. (2017)	Coronary heart disease	Not reported	Hospital admission record	N/A because of study design	N/A because of study design	N/A because of study design	N/A because of study design	Yes	Yes	Yes	Yes	No	OR
McGwin 2005	Ischaemic heart disease	ICD-9 410–414, 416	Healthcare record	N/A because of study design	N/A because of study design	N/A because of study design	N/A because of study design	No	No	Unclear	Yes	No	OR
Kivimaki 2015 - Virtanen et al. (2010)	Coronary heart disease	ICD-9 410–414 or ICD-10 I20-I25	Hospital admission or death record	180	2578	189	3067	Yes	Yes	Yes	Yes	No	Hazard ratio
Kivimaki 2015 - Netterstrøm et al. (2010)	Ischaemic heart disease	ICD-8 410–414, ICD-10 I20-I25	Hospital admission or death record	Not reported	Not reported	Not reported	Not reported	No	N/A	N/A	Unclear	Yes	OR
Kivimaki 2015 - Holtermann 2010	Ischaemic heart disease mortality	ICD-8 410–414, ICD-10 I20-I25	Death record	537	3789	54	584	Yes	Yes	Yes	Yes	No	Hazard ratio
Kivimaki 2015 - Toker et al. (2012)	Ischaemic heart disease	ICD-9 410–414, ICD-10 I21-I25	Hospital admission or death record	Not reported	Not reported	Not reported	Not reported	Yes	Yes	Yes	Yes	No	Hazard ratio
Kivimaki 2015 - O'Reilly 2013	Ischaemic heart disease	ICD-10 I20-I25	Death record	Not reported	Not reported	Not reported	Not reported	Unclear	Unclear	Unclear	Unclear	Unclear	Hazard ratio
Kivimaki 2015 - WOLF-S 1992	Coronary Heart Disease	ICD-9 410–414, ICD-10 I21-I25	Hospital admission or death record	Not reported	Not reported	Not reported	Not reported	Yes	Yes	Yes	No	No	Hazard ratio
Kivimaki 2015 - Belstress 1994	Coronary Heart Disease	ICD-9 410–414, ICD-10 I21-I25	Hospital admission or death record	Not reported	Not reported	Not reported	Not reported	Yes	Yes	Yes	No	No	Hazard ratio
Kivimaki 2015 - WOLF-N 1996	Coronary Heart Disease	ICD-9 410–414, ICD-10 I21-I25	Hospital admission or death record	Not reported	Not reported	Not reported	Not reported	Yes	Yes	Yes	No	No	Hazard ratio
Kivimaki 2015 - COPSOQ-I 1997	Coronary Heart Disease	ICD-9 410–414, ICD-10 I21-I25	Hospital admission or death record	Not reported	Not reported	Not reported	Not reported	Yes	Yes	Yes	No	No	Hazard ratio
Kivimaki 2015 - HeSSup 1998	Coronary Heart Disease	ICD-9 410–414, ICD-10 I21-I25	Hospital admission or death record	Not reported	Not reported	Not reported	Not reported	Yes	Yes	Yes	No	No	Hazard ratio
Kivimaki 2015 - FPS 2000	Coronary Heart Disease	ICD-9 410–414, ICD-10 I21-I25	Hospital admission or death record	Not reported	Not reported	Not reported	Not reported	Yes	Yes	Yes	No	No	Hazard ratio
Kivimaki 2015 - HNR 2000	Coronary Heart Disease	ICD-9 410–414, ICD-10 I21-I25	Hospital admission or death record	Not reported	Not reported	Not reported	Not reported	Yes	Yes	Yes	No	No	Hazard ratio
Kivimaki 2015 - DWECS 2000	Coronary Heart Disease	ICD-9 410–414, ICD-10 I21-I25	Hospital admission or death record	Not reported	Not reported	Not reported	Not reported	Yes	Yes	Yes	No	No	Hazard ratio
Kivimaki 2015 - COPSOQ-II 2004	Coronary Heart Disease	ICD-9 410–414, ICD-10 I21-I25	Hospital admission or death record	Not reported	Not reported	Not reported	Not reported	Yes	Yes	Yes	No	No	Hazard ratio
Kivimaki 2015 - IPAW 1996	Coronary Heart Disease	ICD-9 410–414, ICD-10 I21-I25	Hospital admission or death record	Not reported	Not reported	Not reported	Not reported	Yes	Yes	Yes	No	No	Hazard ratio
Kivimaki 2015 - PUMA 1999	Coronary Heart Disease	ICD-9 410–414, ICD-10 I21-I25	Hospital admission or death record	Not reported	Not reported	Not reported	Not reported	Yes	Yes	Yes	No	No	Hazard ratio
Kivimaki 2015 - NWCS 2005	Coronary Heart Disease	ICD-9 410–414, ICD-10 I21-I25	Hospital admission or death record	Not reported	Not reported	Not reported	Not reported	Yes	Yes	Yes	No	No	Hazard ratio
Kivimaki 2015 - Alameda 1973	Coronary Heart Disease	Not reported	Self-report	Not reported	Not reported	Not reported	Not reported	Yes	Yes	Yes	No	No	OR
Kivimaki 2015 - NHANES I 1982	Coronary Heart Disease	Not reported	Self-report	Not reported	Not reported	Not reported	Not reported	Yes	Yes	Yes	No	No	OR
Kivimaki 2015 - ACL 1986	Coronary Heart Disease	Not reported	Self-report	Not reported	Not reported	Not reported	Not reported	Yes	Yes	Yes	No	No	OR
Kivimaki 2015 - WLSG 1992	Coronary Heart Disease	Not reported	Self-report	Not reported	Not reported	Not reported	Not reported	Yes	Yes	Yes	No	No	OR
Kivimaki 2015 - WLSS 1993	Coronary Heart Disease	Not reported	Self-report	Not reported	Not reported	Not reported	Not reported	Yes	Yes	Yes	No	No	OR
Kivimaki 2015 - MIDUS 1995	Coronary Heart Disease	Not reported	Self-report	Not reported	Not reported	Not reported	Not reported	Yes	Yes	Yes	No	No	OR
Kivimaki 2015 - HILDA 2003	Coronary Heart Disease	Not reported	Self-report	Not reported	Not reported	Not reported	Not reported	Yes	Yes	Yes	No	No	OR
Hannerz et al. (2018)	Ischaemic heart disease	ICD-10 I20-I25	Hospital admission or death record	664	22,479	2779	109,814	Yes	Yes	Yes	Yes	No	Hazard ratio
Hayashi 2019	Acute myocardial infarction	Not reported	Hospital admission or death record	86	7801	76	6520	Yes	No	Yes	Yes	Yes	Hazard ratio

#### 4.2.1 Study type

Most studies were cohort studies (26 studies), followed by case-control studies (11). The type of effect estimates most commonly reported were ORs (19 studies) and hazard ratios (18 studies). All included studies were adjusted for our three pre-specified minimum confounders. However, several case-control studies in additional also adjusted for further potential confounders (Table 5).

#### 4.2.2 Population studied

The included studies captured 768,751 workers (310,954 females and 457,797 males). The studies were of females only, males only, or both female and male workers. The most commonly studied age groups were those between 20 and 65 years.

By WHO region, most studies examined populations in the European region (20 studies from eight countries), followed by populations in the Americas (nine studies from one country) and populations in the Western Pacific (eight studies from four countries). The most commonly studied countries were the United States (nine studies), Denmark (eight), Japan (four) and Sweden (three). Most studies did not provide quantitative break downs of participants by industrial sectors and occupation, but they did appear to cover several industrial sectors and occupations.

#### 4.2.3 Exposure studied

All studies measured exposure to long working hours with either self-reported questionnaire or face-to-face interview. Other measures such as official or company records of hours worked were not used.

#### 4.2.4 Comparator studied

The comparator for most studies was 35–40 h/week. One of the 26 cohort studies (Hayashi et al., 2019) used a comparator of 7 to <9 h/day, which we judged to be comparable to 35–40 h/week. Nine out of 11 case-control studies used slightly different comparators (Virtanen et al., 2012 – Russek and Zohman, 1958, Virtanen et al., 2012 - Theorell and Rahe, 1972, Virtanen et al., 2012 - Thiel et al., 1973, Virtanen et al., 2012 – Falger and Schouten, 1992, Virtanen et al., 2012 – Sokejima and Kagamimori, 1998, Virtanen et al., 2012 - Liu and Tanaka, 2002, Cheng et al., 2014, Ma et al., 2017, McGwin, 2005).

#### 4.2.5 Outcomes studied

No studies reported evidence on the outcome of IHD prevalence.

Thirty-five studies (24 cohort studies and 11 case-control studies) reported evidence on the outcome “Acquired IHD” (or IHD incidence). Of these, 19 studies (8 cohort studies and 11 case-control studies) defined the outcome as incidence of a non-fatal IHD event, and 16 studies (all cohort studies) as an incident IHD event that was either non-fatal or fatal (“mixed”).

Eighteen studies (all cohort studies) reported evidence on the outcome “Died from IHD” (or IHD mortality). Two of these studies defined the outcome as a fatal IHD event, and the remaining 16 studies used a “mixed” outcome definition including both fatal and/or non-fatal IHD events.

Outcome assessment was by administrative health records in 30 studies and self-reported physician diagnosis in seven studies (Appendix 4 in the Supplementary data for questions).

### 4.3 Risk of bias at individual study level

#### 4.3.1 Acquired IHD (IHD incidence)

**Fig. 3:**
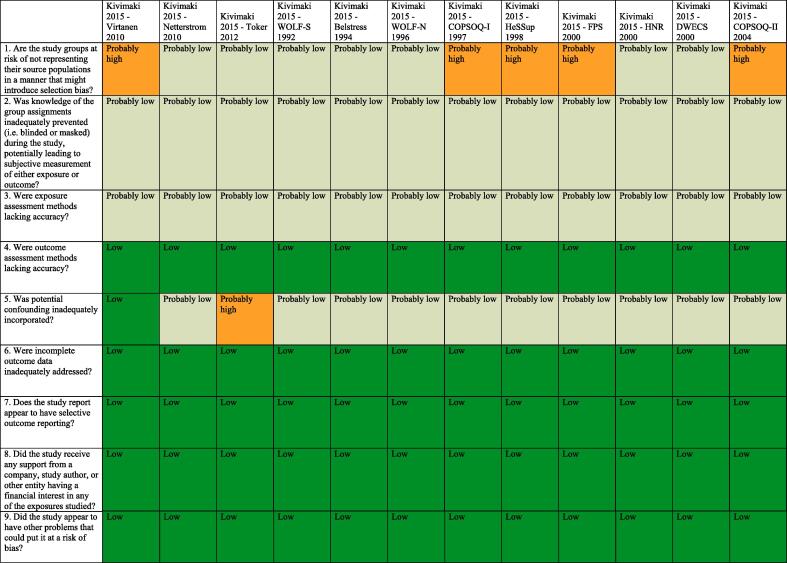

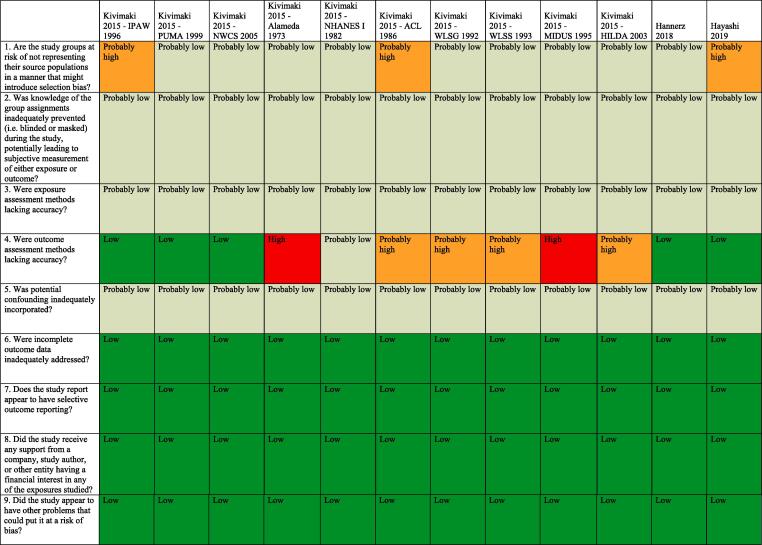

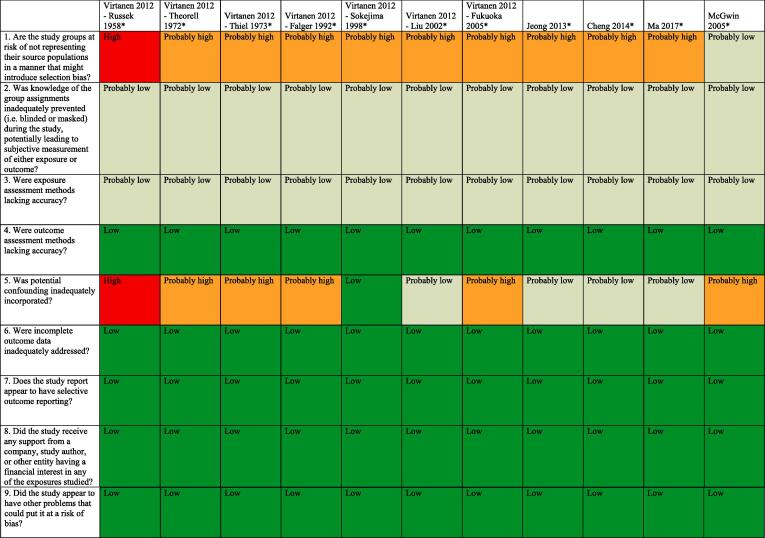
Summary of risk of bias, Acquired ischaemic heart disease (IHD incidence). Footnotes: * Case-control study (supporting evidence).

The risk of bias rating for each domain for all 35 included studies for this outcome are presented in Fig. 3. The justification for each rating for each domain by included study is presented in Appendix 5 in the Supplementary data. We prioritized the evidence from the 24 cohort studies included in our systematic review for this outcome as the main evidence for the outcome, because we judged evidence from these studies to carry relatively less risk of bias, and we deprioritized the evidence from case-control studies as supporting evidence. Therefore, for assessing the quality of evidence for this outcome (see Section 4.6. Quality of evidence), we assessed the risk of bias in the body of evidence for this outcome based on risk of bias in the prioritized studies (cohort studies) only, rather than in the entire or supporting evidence.

##### 4.3.1.1 Selection bias

We assessed risk of bias in this domain based on whether the groups being compared were the same in all relevant ways (or as close to this as possible) apart from the exposure. Of the 24 included cohort studies, the risk of selection bias was rated to be probably high for one study due to unavailability of follow-up data in one region out of six regions and male workers only (Hayashi et al., 2019). However, we are aware that unavailability of one region does not necessarily suggest increased risk of selection bias as the same exposure-outcome effect can be expected. We rated the risk of selection bias as probably high for seven studies because these studies included only a specific subpopulation or selected industrial sector and reported only a low study participation rate (Kivimaki 2015 - Virtanen et al., 2010, Kivimaki 2015 - COPSOQ-I 1997, Kivimaki 2015 - HeSSup 1998, Kivimaki 2015 - FPS 2000, Kivimaki 2015 - COPSOQ-II 2004, Kivimaki 2015 - IPAW 1996, Kivimaki 2015 - ACL 1986). The risk of this bias in the other 16 studies was rated as probably low, because these studies provided indirect evidence that captured large, probably representative populations; these studies described their sample criteria extensively, enabling comparisons with the source population, while at the same time providing indirect evidence on acceptable inclusion criteria, recruitment and enrolment procedures, and participation rates (Fig. 3).

For case-control studies, the risk of selection bias was rated high in one study, probably high in nine studies, and probably low in one study (Appendix 5 in the Supplementary data for ratings given by included study). Unlike cohort studies, most case-control studies due to their high selection bias did not adequately represent the source population (Appendix 5 for justification for the ratings for each included study).

##### 4.3.1.2 Performance bias

For the included cohort studies, blinding of study participants and study personnel to assignments of study participants to exposure to long working hour and to study participants’ characteristics was usually not reported in the study’s record or records. There is a likely minor and negligible risk of bias that knowledge of such exposure assignment and/or study participants’ characteristics could have impacted the reporting and/or analysis of the estimated impact of the exposure on the outcome. The outcome is mostly measured using administrative data, and this further reduced our concerns for risk of detection bias for the outcome. Therefore, we rated all studies as probably low risk (Fig. 3; Appendix 5 in the Supplementary data).

For case-control studies, the risk of performance bias was rated as probably low in all studies. Although study participants and study personnel were blinded to neither exposure assignment, nor study participants’ characteristics, it is unlikely that this lack of blinding impacted analysis and reporting of exposure-outcome associations.

##### 4.3.1.3 Detection bias (exposure assessment)

For cohort studies, although an objective assessment of the exposure would have reduced the risk of detection bias for the exposure assessment, we judged the uniform standard self-report assessment of exposure to not have introduced noteworthy risk of detection bias. Self-report of (long) working hours has been validated against objective measures of long working hours (e.g., Imai et al., 2016), and, in our opinion, it is therefore unlikely that use of self-reported exposure in the included studies introduced any substantial detection bias. We consequently rated all studies as carrying probably low risk of detection bias in the exposure assessment.

For case-control studies, we rated risk of performance bias as probably low in all studies. We again judged that self-report of (long) working hours was unlikely to have introduced any substantial risk of detection bias, since such subjective measures have been validated against objective measures of long working hours (e.g., Imai et al., 2016).

##### 4.3.1.4 Detection bias (outcome assessment)

For the included cohort studies, the 16 studies with a “mixed” outcome definition that comprised both fatal and non-fatal IHD events used administrative data, namely either physician-based clinical diagnoses or ICD-coded records. Therefore, we rated risk of detection bias for these studies as low. However, seven studies used self-report outcome data. There is some evidence that self-reported diagnoses of IHD events are valid measurements (Muggah et al., 2013). However, we rated the risk of detection bias in these studies (Kivimaki 2015 - ACL 1986, Kivimaki 2015 - Alameda 1973, Kivimaki 2015 - HILDA 2003, Kivimaki 2015 - MIDUS 1995, Kivimaki 2015 - WLSG 1992, Kivimaki 2015 - WLSS 1993) as either probably high or high, because the wording used in the survey questions to assess the outcome was unspecific (see Appendix 4 in the Supplementary data). We considered whether inclusion of recurrent IHD events might have produced any misclassification bias of the outcome, but did not find any study that reported data on both first and recurrent IHD events that would allow evaluation of any potential risk of such bias.

For case-control studies, all studies were rated as low risk of detection bias, because the outcome assessment had a high accuracy, given appropriate quality of assessment methods (clinical diagnosis of IHD patients).

##### 4.3.1.5 Confounding

Of the 24 cohort studies included in the systematic review for this outcome, 23 studies:

• appropriately adjusted or controlled for all (or at least most) of our three pre-specified potential confounders that studies should adjust or control for (i.e., age, sex, and socioeconomic position);
• if they additionally controlled their effect estimates for further variables that could be confounders, mediators and/or moderators and/or reported that they adjusted or controlled for additional such variables, but found that these adjustments or controls did not affect the effect estimates, then we judged these additional adjustments to not carry risk of confounding; and
• used appropriate statistical techniques for confounder adjustment and/or control, with the exception of one study which did not apply the requested statistical approaches for adjustment of confounding factors (Kivimaki 2015 - Toker et al., 2012).

Overall, we consequently judged these 23 studies to have probably low risk of confounding.

One study (Kivimaki 2015 - Toker et al., 2012) potentially over-adjusted for working hours and burnout, and we therefore judged this study to have probably high risk of confounding.

Of the seven case-control studies, we rated one study as having high risk, five studies as having probably high risk, and one as having probably low risk of confounding. Our ratings of high risk of confounding were justified by a total lack of adjustment and/or control for the three pre-specified potential confounders. Our ratings of probably high risk of confounding were justified by insufficient such confounder adjustment/control.

##### 4.3.1.6 Selection bias (incomplete outcome data)

We judged that in all 24 cohort studies included for this outcome:

• the proportion of invited persons who participated in the study was acceptably high;
• the proportion of study participants who were lost to follow-up over time was acceptably low;
• the study followed up study participants sufficiently long after exposure to long working hours for them to reasonably have acquired the outcome;
• the proportion of outcome data that was missing at baseline was acceptably low;
• the proportion of outcome data missing at final follow up was acceptably low (i.e., <50%);
• there was balance across exposure groups in the survey non-response at baseline, item non-response at baseline, missing participants at final follow up and missing outcome data at final follow-up, with similar reasons for missing study participants and/or outcome data across groups (if reported); and/or
• the missing outcome data were imputed using appropriate statistical methods.

Based on these considerations, we judged all the cohort studies to have low risk of selection bias due to incomplete outcome data.

For all case-control studies, outcome data were complete, with no outcome data missing from any study participant. Given the case-control design, risk of selection bias due to incomplete outcome data was unlikely as no study participants were lost during follow-up. We are aware that a potential risk of selection bias due to incomplete outcome data may be due to differential item non-responses, but respective information was not available from published studies and was judged as unlikely. We therefore rated these studies as having a low risk of bias in this domain.

##### 4.3.1.7 Reporting bias

In all cohort studies with pre-published protocols the outcomes were reported in the included study record as they had been pre-specified in the protocol. In the cohort studies without a pre-published protocol, the outcomes were reported in the results sections of the study records as they had been reported in the abstracts and methods sections in the study record. We also did not find any other evidence that reporting may have been biased. We consequently judged risk of reporting bias as low in all included cohort studies.

For case-control studies, reporting bias is unlikely as all of the study’s pre-specified outcomes outlined in the pre-published protocol or the published manuscript have been reported in the pre-specified way. Therefore, all studies were rated as low risk of this bias.

##### 4.3.1.8 Conflict of interest

All cohort studies included for this outcome:

• did not receive support from a company or other entity with a financial interest in the study findings;
• were funded by public research agencies or related organizations that were free from commercial interests in the study findings;
• were authored only by persons who were not affiliated with companies or other entities with vested interests; and/or;
• had no conflict of interest declared by study authors.

Therefore, we rated all studies as having low risk of bias from conflict of interest.

Similarly, we judged all case-control studies to have low bias in this domain, because these studies were also conducted exclusively by researchers that were publicly funded, and we also again found no evidence of commercial interests influencing these studies.

##### 4.3.1.9 Other risk of bias

We did not find any evidence for any risk of other types of bias in any included cohort or case-control study and therefore judged all included cohort and case-control studies to have a low risk of other bias.

#### 4.3.2 Died from IHD (IHD mortality)

**Fig. 4:**
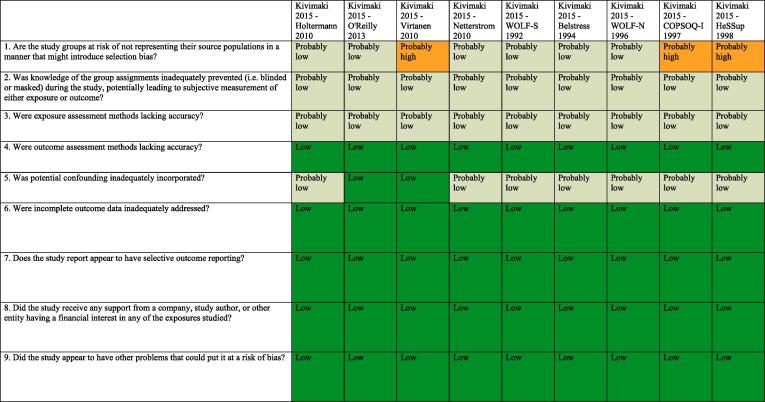

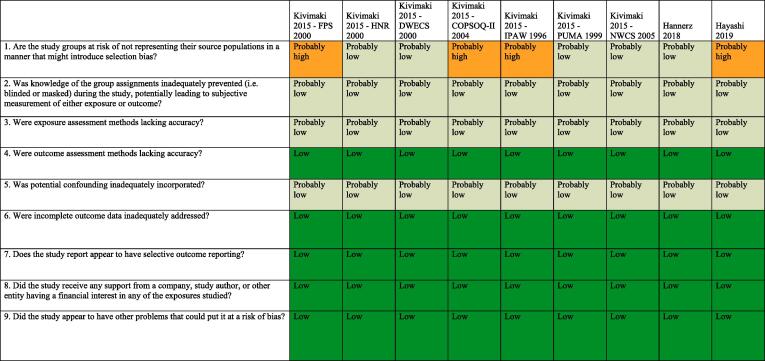
Summary of risk of bias, Died from ischaemic heart disease (IHD mortality).

The risk of bias rating for each domain for all 18 included studies for this outcome are presented in Fig. 4, and our detailed justifications for each rating for each domain are again shown in Appendix 5 in the Supplementary data.

##### 4.3.2.1 Selection bias

For the 18 cohort studies, this bias was rated probably high for one study, due to unavailability of follow-up data in one region out of six regions, and male workers only (Hayashi et al., 2019). In addition, six studies were rated as probably high because of only a specific population or industry being included and, and low study participation rate (Kivimaki 2015 - Virtanen et al., 2010, Kivimaki 2015 - COPSOQ-I 1997, Kivimaki 2015 - HeSSup 1998, Kivimaki 2015 - FPS 2000, Kivimaki 2015 - COPSOQ-II 2004, Kivimaki 2015 - IPAW 1996). This bias was rated as probably low for the other 11 studies because these studies comprised large populations of working age and described their sample criteria extensively, enabling comparisons with the source population, while at the same time providing indirect evidence only on inclusion criteria, recruitment and enrolment procedures, and participation rates (Fig. 4).

##### 4.3.2.2 Performance bias

For the included cohort studies, blinding of study participants and study personnel to assignments of study participants to exposure to long working hours and to study participants’ characteristics was usually not reported in the study’s record or records. There is a likely minor and negligible risk of bias that knowledge of such exposure assignment and/or study participants’ characteristics could have impacted the reporting and/or analysis of the estimated impact of the exposure on the outcome. The outcome is mostly measured using administrative data, and this further reduced our concerns for risk of detection bias for the outcome. Therefore, we rated all studies as probably low risk of performance bias (Fig. 4).

##### 4.3.2.3 Detection bias (exposure assessment)

Although an objective assessment of the exposure would have reduced the risk of detection bias for the exposure assessment, we judged the uniform standard self-report assessment of exposure to not have introduced noteworthy risk of detection bias. Self-report of (long) working hours has been validated against objective measures of long working hours (e.g., Imai et al., 2016), and in our opinion it is therefore unlikely that use of self-reported exposure in the included studies introduced any substantial detection bias. We consequently rated all studies as probably low risk of detection bias in the exposure assessment.

##### 4.3.2.4 Detection bias (outcome assessment)

All studies with a “mixed” outcome definition that comprised both fatal and non-fatal IHD events used administrative data, namely either physician-based clinical diagnoses or ICD-coded records. Two studies used the death register data with specific ICD codes that were very accurate. Therefore, we rated the risk of detection bias for all these studies as low.

##### 4.3.2.5 Confounding

Of the 18 cohort studies included in the systematic review for this outcome, 16 studies:

• appropriately adjusted or control for all (or at least most) of our three-ore-specified potential confounders that studies should adjust or control for (i.e., age, sex, and socioeconomic position).
• if they additionally controlled their effect estimates for further variables that could be confounders, mediators and/or moderators and/or reported that they adjusted or controlled for additional such variables, but found that these adjustments or controls did not affect the effect estimates, then we judged these additional adjustments to not carry risk of confounding; and
• used appropriate statistical techniques for confounder adjustment and/or control.

Overall, we consequently judged these 16 studies to have probably low risk of confounding. Two studies (Kivimaki 2015 - O'Reilly 2013, Kivimaki 2015 - Virtanen et al., 2010) with comprehensive statistical techniques for confounder adjustment and/or control were rated as low risk of confounding.

##### 4.3.2.6 Selection bias (incomplete outcome data)

We judged that in all 18 cohort studies included for this outcome:

• the proportion of invited persons who participated in the study was acceptably high;
• the proportion of study participants who were lost to follow-up over time was acceptably low;
• the study followed up study participants sufficiently long after exposure to long working hours for them to reasonably have acquired the outcome;
• the proportion of outcome data that was missing at baseline was acceptably low;
• the proportion of outcome data missing at final follow up was acceptably low (i.e. <50%);
• there was balance across exposure groups in the survey non-response at baseline, item non-response at baseline, missing participants at final follow up and missing outcome data at final follow-up, with similar reasons for missing study participants and/or outcome data across groups (if reported); and/or
• the missing outcome data were imputed using appropriate statistical methods.

Based on these considerations, we judged all the studies to have low risk of selection bias due to incomplete outcome data.

##### 4.3.2.7 Reporting bias

In all cohort studies with pre-published protocols the outcomes were reported in the included study record as they had been pre-specified in the protocol. In the cohort studies without a pre-published protocol, the outcomes were reported in the results sections of the study records as they had been reported in the abstracts and methods sections in the study record, and we also did not find any other evidence that reporting may have been biased. We consequently judged risk of reporting bias as low in all included cohort studies.

##### 4.3.2.8 Conflict of interest

All cohort studies included for this outcome:

• did not receive support from a company or other entity with a financial interest in the study findings;
• were funded by public research agencies or related organizations that were free from commercial interests in the study findings;
• were authored only by persons who were not affiliated with companies or other entities with vested interests; and/or
• had no conflict of interest declared by study authors.

Therefore, we judged all studies as having low risk of bias from conflict of interest.

##### 4.3.2.9 Other risk of bias

We did not find any evidence for any risk of other types of bias in any included cohort studies and therefore judged all included cohort studies to have a low risk of other bias.

### 4.4 Synthesis of results

#### 4.4.1 Outcome: Has IHD (IHD prevalence)

No eligible study was found on the effect of long working hours on IHD prevalence.

#### 4.4.2 Outcome: Acquired IHD (IHD incidence)

##### 4.4.2.1 Comparison: Worked 41–48 h/week compared with worked 35–40 h/week

A total of 27 studies (20 cohort studies and seven case-control studies) with a total of 315,723 participants reported data on this comparison for this outcome. We meta-analysed evidence from cohort studies separately from that from case-control studies. In our risk of bias assessment for the outcome (Section 4.3.1), we judged cohort studies to carry a relatively lower risk of bias than case-control studies and consequently prioritize evidence from cohort studies over that from case-control studies. Our main meta-analysis for this comparison for this outcome is consequently that of the included cohort studies.

**Fig. 5:**
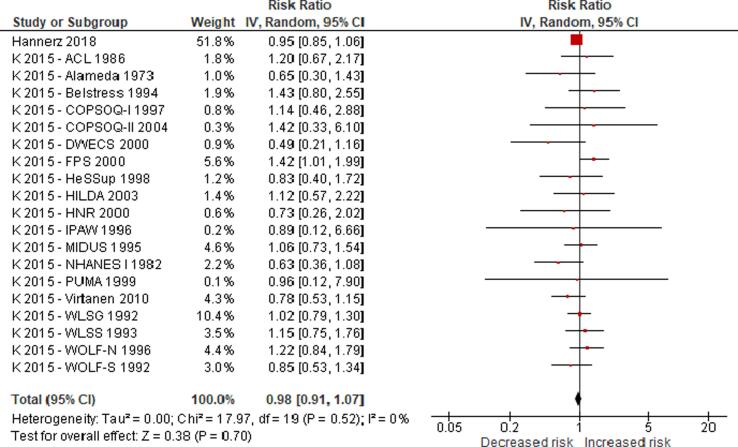
Main meta-analysis of prioritized evidence (cohort studies), Outcome: Acquired ischaemic heart disease, Comparison: Worked 41–48 h/week compared with worked 35–40 h/week.

Twenty cohort studies with a total of 312,209 participants from three WHO regions reported estimates of the effect of exposure to long working hours on the risk of acquiring IHD when working 41–48 h/week, compared with 35–40 h/week. These studies were somewhat heterogeneous in that seven studies defined the outcome as a non-fatal IHD event (Alameda, NHANES I, ACL, WLSG, WLSS, MIDUS, and HILDA), whereas 13 of the studies defined the outcome as a non-fatal or fatal (or “mixed”) IHD event (Hannerz et al., 2018, WOLF-S, Belstress, WOLF-N, COPSOQ-I, HeSSup, FPS, HNR, DWECS, COPSOQ-II, IPAW, PUMA, and NWCS). Because fatal and non-fatal IHD events share an identical pathophysiological basis we considered studies with pure non-fatal events and studies with both fatal and non-fatal (“mixed”) events to be sufficiently homogenous clinically be included in the same meta-analysis. Moreover, subgrouping pure non-fatal and “mixed” event studies demonstrated no evidence for subgroup differences (Appendix 6 in the Supplementary data), suggesting that these studies are sufficiently homogenous statistically to be combined. Therefore, we judged the heterogeneity of the included studies to be sufficiently low overall, and we consequently combined all included studies in one meta-analysis. This has also been done in previous meta-analyses (Kivimaki et al., 2015, Virtanen and Kivimaki, 2018). Compared with working 35–40 h/week, working 41–48 h/week led to a risk of about 1 of acquiring IHD (relative risk (RR) 0.98, 95% CI 0.91 to 1.07, 20 studies, 312,209 participants, I^2^ 0%; Fig. 5).

**Fig. 6:**
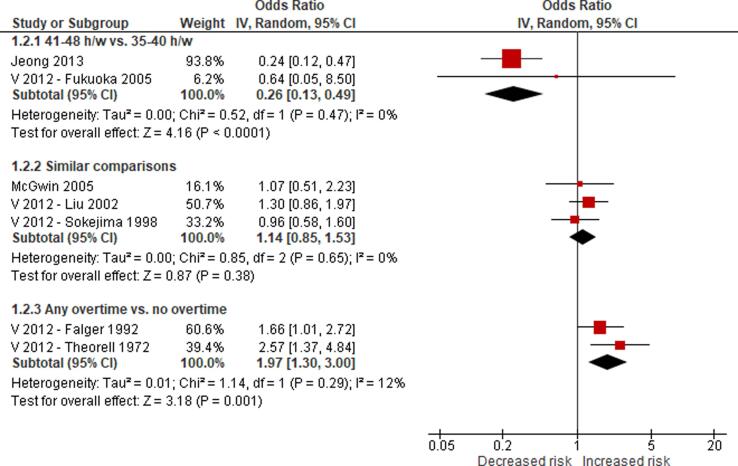
Supporting meta-analysis of deprioritized evidence (case-control studies), Outcome: Acquired ischaemic heart disease, Comparison: Worked 41–48 h/week compared with worked 35–40 h/week (or similar comparisons, or any overtime work). Footnotes: The similar comparisons included in this meta-analysis were: McGwin 2005: 41-50 h/w vs. <40 h/w; Virtanen 2012 - Liu and Tanaka, 2002: 41 to 60 h/w vs. ≤40 h/w; and Virtanen 2012 - Sokejima 1998: 9.01 to 11.00 h/d vs. 7.01 to 9.00 h/d.

Of the seven case-control studies with eligible evidence including a total of 3514 participants, two studies provided evidence with the exact definition of the exposure and reference categories (Fukuoka et al., 2005, Jeong et al., 2013). We considered these two studies to be sufficiently homogenous to be combined in a quantitative meta-analysis. Compared with working 35–40 h/week, working 41–48 h/week led to a reduction in risk of acquiring IHD (OR 0.26, 95% CI 0.13 to 0.49, 2 studies, 962 participants, I^2^ 0%; Fig. 6). Three case-control studies used similar comparisons (Virtanen et al., 2012 – Sokejima and Kagamimori, 1998, Virtanen et al., 2012 - Liu and Tanaka, 2002, McGwin, 2005). Compared with working ≤40 or <45 h/week, there was an elevated OR with a lower confidence bound that crossed 1 that working 40–60 h/week (or a comparable number of hours) had any effect on the risk of acquiring IHD (OR 1.14, 95% CI 0.85 to 1.53, 3 studies, 1923 participants, I^2^ 0%; Fig. 6). Moreover, two studies conducted several decades ago compared any overtime work with no overtime work (Virtanen et al., 2012 – Falger and Schouten, 1992, Virtanen et al., 2012 - Theorell and Rahe, 1972), indicating an increase in the risk of acquiring IHD (OR 1.97, 95% CI 1.30 to 3.00, 2 studies, 629 participants, I^2^ 12%; Fig. 6). When we combined the three subgroups defined by exposure categories in one meta-analysis, our test for subgroup differences found statistically significant differences, and we therefore turned the overall pooled effect estimate off and only report subtotals for each subgroup. In addition, two case-control studies were not included in any meta-analysis because they used different comparators. One of these studies reported that compared with working 40–48 h/week, working <40 h/weeks increased the risk of acquiring IHD with the lower confidence bound crossing 1 (OR 1.5, 95% CI 0.9 to 2.3, 966 participants) (Cheng et al., 2014). The second study reported that compared with working zero hours/week, working 41–48 h/weeks increased the risk of acquiring IHD with the lower confidence bound crossing 1 (OR 1.44, 95% CI 0.76 to 2.73, 595 participants) (Ma et al., 2017).

##### 4.4.2.2 Comparison: Worked 49–54 h/week compared with worked 35–40 h/week

A total of 24 studies (18 cohort studies and six case-control studies) with a total of 311,227 participants reported data on this comparison for this outcome. We again meta-analysed evidence from cohort studies separately from that from case-control studies and prioritize evidence from cohort studies over that from case-control studies, for the reasons outline above (Section 4.4.2.1). Our main meta-analysis for this comparison for this outcome again is also that of the eligible cohort studies.

**Fig. 7:**
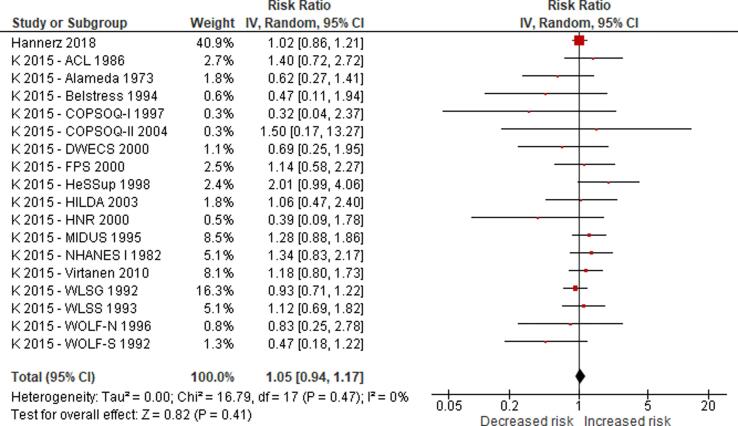
Main *meta*-analysis of prioritized evidence (cohort studies), Outcome: Acquired ischaemic heart disease, Comparison: Worked 49–54 h/week compared with worked 35–40 h/week.

Eighteen cohort studies with a total of 308,405 participants from three WHO regions reported estimates of the effect of exposure to long working hours on the risk of acquiring IHD when working 49–54 h/week, compared with working 35–40 h/week. The included studies were again somewhat heterogeneous in outcome definition, with seven studies defining the outcome as a non-fatal IHD event (Alameda, NHANES I, ACL, WLSG, WLSS, MIDUS, and HILDA) and 11 studies defining the outcome as a non-fatal or fatal (or “mixed”) IHD event (Hannerz et al., 2018, WOLF-S, Belstress, WOLF-N, COPSOQ-I, HeSSup, FPS, HNR, DWECS, COPSOQ-II, and NWCS). As with the previous comparison for the same outcome, we again judged these studies to be sufficiently homogenous clinically to potentially be combined, again also found no evidence for subgroup differences between studies defined by these outcome definitions (Appendix 6 in the Supplementary data), and therefore again decided to combine these studies in one meta-analysis, as has also been done previously (Kivimaki et al., 2015, Virtanen and Kivimaki, 2018). Compared with working 35–40 h/week, working 49–54 h/week led to an elevated risk of acquiring IHD with the lower confidence bound being below 1 (RR 1.05, 95% CI 0.94 to 1.17, 18 studies, 308,405 participants, I^2^ 0%; Fig. 7).

**Fig. 8:**
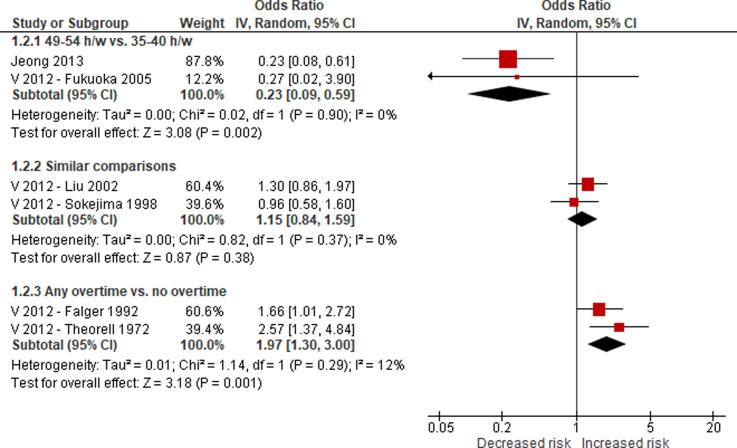
Supporting *meta*-analysis of deprioritized evidence (case-control studies), Outcome: Acquired ischaemic heart disease, Comparison: Worked 49–54 h/week compared with worked 35–40 h/week (or similar comparisons, or any overtime work). Footnotes: The similar comparisons included in the meta-analysis were: Virtanen 2012 - Liu and Tanaka, 2002: 41 to 60 h/w vs. ≤40 h/w and Virtanen 2012 - Sokejima 1998: 9.01 to 11.00 h/d vs. 7.01 to 9.00 h/d.

Of the six case-control studies with eligible evidence including a total of 2,822 participants, two provided evidence with the exact definition of the exposure and reference categories (Fukuoka et al., 2005, Jeong et al., 2013). We considered these studies to be sufficiently homogenous clinically to be combined in a quantitative meta-analysis. There was a reduction for effect of working 49–54 compared with 35–40 h/week on the risk of acquiring IHD (OR 0.23, 95% CI 0.09 to 0.59, 2 studies, 962 participants, I^2^ 0%; Fig. 8). Two case-control studies used similar comparisons (Virtanen et al., 2012 – Sokejima and Kagamimori, 1998, Virtanen et al., 2012 - Liu and Tanaka, 2002). Compared with working ≤40 or <45 h/week, there was an elevated risk with lower CI below 1 that working 41–60 h/week (or a comparable number of hours) had an effect on the risk of incident IHD (OR 1.15, 95% CI 0.84 to 1.59, 2 studies, 1231 participants, I^2^ 0%; Fig. 8). Moreover, two studies conducted several decades ago compared any overtime work with no overtime work (Virtanen et al., 2012 – Falger and Schouten, 1992, Virtanen et al., 2012 - Theorell and Rahe, 1972), with our meta-analysis of these studies finding an increase in the risk of acquiring IHD (RR 1.97, 95% CI 1.30 to 3.00, 2 studies, 629 participants, I^2^ 12%; Fig. 8). As with the previous comparison (Section 4.4.2.1), when we combined the three subgroups defined by exposure categories in one meta-analysis, our test for subgroup differences again found statistically significant differences, and we therefore again turned the overall pooled effect estimate off and only report subtotals for each subgroup. In addition, two case-control studies were not included into meta-analysis because of different comparators: compared with working 40–48 h/week, those working 49–60 h/weeks had an increase in the risk of acquiring IHD (OR 1.6, 95% CI 1.2 to 2.2, 966 participants) (Cheng et al., 2014); compared with working zero hours/week, there was an elevated risk with the lower CI below 1 and the upper almost 3 that working 49–54 h/weeks had effect on the risk of acquiring IHD (OR 1.38, 95% CI 0.67 to 2.86, 595 participants) (Ma et al., 2017).

##### 4.4.2.3 Comparison: Worked ≥55 h/week compared with worked 35–40 h/week

A total of 31 studies (22 cohort studies and nine case-control studies) with a total of 343,494 participants reported data on this comparison for this outcome. We again meta-analysed evidence from cohort studies separately from that from case-control studies; prioritized evidence from cohort studies over that from case-control studies; and use as our main meta-analysis that of the eligible cohort studies, for the reasons already detailed in 4.4.2.1, 4.4.2.2.

**Fig. 9:**
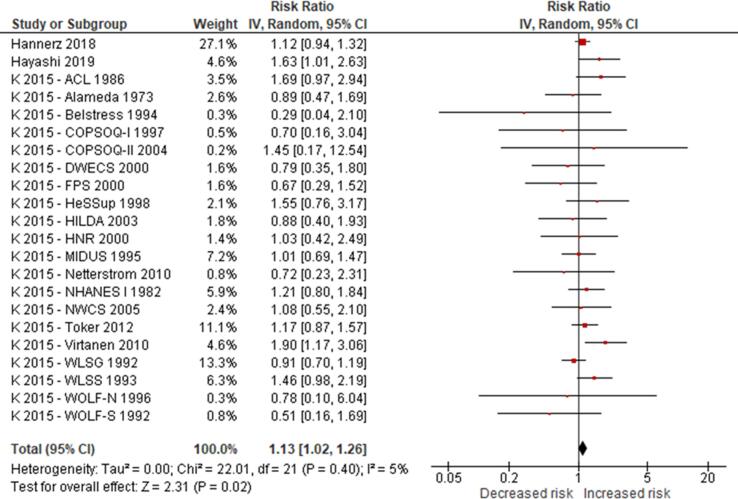
Main meta-analysis of prioritized evidence (cohort studies), Outcome: Acquired ischaemic heart disease, Comparison: Worked ≥55 h/week compared with worked 35–40 h/week.

Twenty-two cohort studies with a total of 339,680 participants from three WHO regions reported estimates of the effect of exposure to long working hours on the risk of acquiring IHD when working ≥55 h/week, compared with working 35–40 h/week. All these studies could be included in a quantitative meta-analysis. These studies that we pooled in our meta-analysis were somewhat heterogeneous in that eight studies defined the outcome as a non-fatal IHD event (Alameda, NHANES I, ACL, WLSG, WLSS, MIDUS, HILDA, and Toker et al., 2012), whereas 14 of the studies defined the outcome as a non-fatal or fatal (or “mixed”) IHD event (Netterstrøm et al., 2010, Virtanen et al., 2010, Hannerz et al., 2018, Hayashi 2019¸WOLF-S, Belstress, WOLF-N, COPSOQ-I, HeSSup, FPS, HNR, DWECS, COPSOQ-II, NWCS). As with both previous comparisons for the same outcome, we again judged these studies to be sufficiently homogenous clinically to potentially be combined, again also found no evidence for subgroup differences between studies defined by these outcome definitions (Appendix 6 in the Supplementary data), and therefore again decided to combine these studies in one meta-analysis. In our meta-analysis, compared with working 35–40 h/week, working ≥55 h/week was associated with an elevated risk with lower CI above 1 of acquiring IHD (relative risk (RR) 1.13, 95% CI 1.02 to 1.26, 22 studies, 339,680 participants, I^2^ 5%; Fig. 9).

**Fig. 10:**
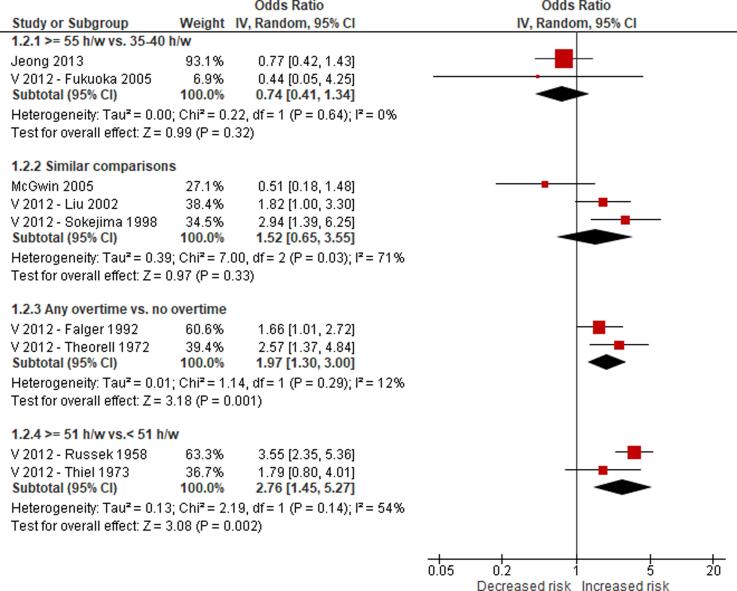
Supporting meta-analysis of deprioritized evidence (case-control studies), Outcome: Acquired ischaemic heart disease, Comparison: Worked ≥55 h/week compared with worked 35–40 h/week (or similar comparisons, or any overtime work). Footnotes: The similar comparisons included in this meta-analysis were: McGwin 2005: >50 h/w vs. <40 h/w; Virtanen 2012 - Liu and Tanaka, 2002: ≥61 h/w vs. ≤40 h/w; and Virtanen 2012 - Sokejima and Kagamimori (1998): ≥11.01 h/d vs. 7.01 to 9.00 h/d.

Of the nine case-control studies with eligible evidence including a total of 3814 participants, 2 provided evidence with the exact definition of the exposure and reference categories (Fukuoka et al., 2005, Jeong et al., 2013). We considered these studies to be sufficiently homogenous with regard to the defined outcome criteria to be combined in a quantitative meta-analysis. There was no evidence for any effect of working ≥55 compared with 35–40 h/week on the risk of acquiring IHD (OR 0.74, 95% CI 0.41 to 1.34, 2 studies, 962 participants, I^2^ 0%; Fig. 10). Three case-control studies used similar comparisons (Virtanen et al., 2012 – Sokejima and Kagamimori, 1998, Virtanen et al., 2012 - Liu and Tanaka, 2002, McGwin, 2005). Compared with working ≤40 or <45 h/week, working >50 h/week (or a comparable number of hours) led to an increase in the risk of incident IHD with lower CI below 1 (OR 1.52, 95% CI 0.65 to 3.55, 3 studies, 1923 participants, I^2^ 71%; Fig. 10). Moreover, two studies conducted several decades ago compared any overtime work with no overtime work (Virtanen et al., 2012 – Falger and Schouten, 1992, Virtanen et al., 2012 - Theorell and Rahe, 1972), finding an elevated risk of acquiring IHD by an estimated 97% (OR 1.97, 95% CI 1.30 to 3.00, 2 studies, 629 participants, I^2^ 12%; Fig. 10); two studies conducted several decades ago compared worked ≥51 h/w with worked <51 h/w (Virtanen et al., 2012 - Russek, 1958, Virtanen et al., 2012 - Lthiel 1973), also finding an increase in the risk of acquiring IHD by an estimated 176% (OR 2.76, 95% CI 1.45 to 5.27, 2 studies, 300 participants, I^2^ 54%; Fig. 10). When we combined the four subgroups defined by exposure categories in one meta-analysis, our test for subgroup differences found statistically significant differences, and we therefore turned the overall pooled effect estimate off and only report subtotals for each subgroup. In addition, 2 case-control studies were not included into meta-analysis because of different comparators: compared with working 40–48 h/week, those working >60 h/weeks had an increase in the risk of acquiring IHD (OR 2.2, 95% CI 1.6 to 3.1, 966 participants) (Cheng et al., 2014); compared with working zero hours/week, those working ≥55 h/weeks had an increase in the risk of acquiring IHD (OR 2.21, 95% CI 1.12 to 4.36, 595 participants) (Ma et al., 2017).

#### 4.4.3 Outcome: Died from IHD (IHD mortality)

##### 4.4.3.1 Comparison: Worked 41–48 h/week compared with worked 35–40 h/week

**Fig. 11:**
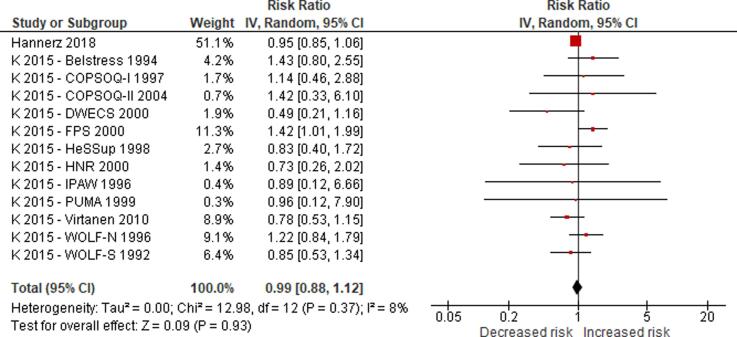
Main meta-analysis of cohort studies, Outcome: Died from ischaemic heart disease, Comparison: Worked 41–48 h/week compared with worked 35–40 h/week.

A total of 13 cohort studies with a total of 288,278 participants from one WHO region reported estimates of the effect of exposure to long working hours on the risk of dying from IHD when working 41–48 h/week, compared with 35–40 h/week. All these studies defined the outcome as a non-fatal or fatal (or “mixed”) IHD event. All these studies could be included in a quantitative meta-analysis. We found that compared with working 35–40 h/week, working 41–48 h/week was associated with a near equal (1) risk of dying from IHD (RR 0.99, 95% CI 0.88 to 1.12, 13 studies, 288,278 participants, I^2^ 8%; Fig. 11).

##### 4.4.3.2 Comparison: Worked 49–54 h/week compared with worked 35–40 h/week

**Fig. 12:**
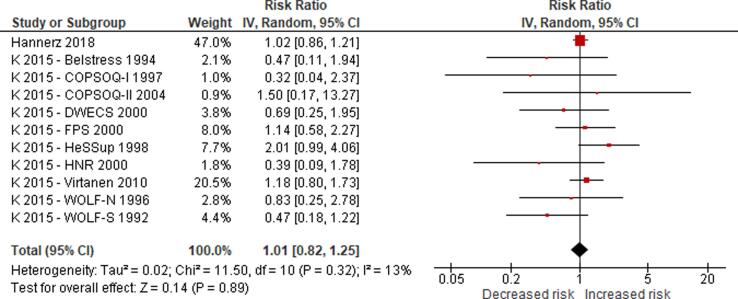
Main meta-analysis of cohort studies, Outcome: Died from ischaemic heart disease, Comparison: Worked 49–54 h/week, compared with worked 35–40 h/week.

A total of 11 cohort studies with a total of 284,474 participants from one WHO region reported estimates of the effect of exposure to long working hours on the risk of dying from IHD when working 49–54 h/week, compared with 35–40 h/week. Again, all these included studies defined the outcome as a non-fatal or fatal (or “mixed”) IHD event, and we again judged these studies to be sufficiently homogenous clinically to be combined in a meta-analysis. We found that compared with working 35–40 h/week, working 49–54 h/week there was an elevated risk with lower confidence bound below 1 of dying from IHD (RR 1.01, 95% CI 0.82 to 1.25, 11 studies, 284,474 participants, I^2^ 13%; Fig. 12).

##### 4.4.3.3 Comparison: Worked ≥55 h/week compared with worked 35–40 h/week

**Fig. 13:**
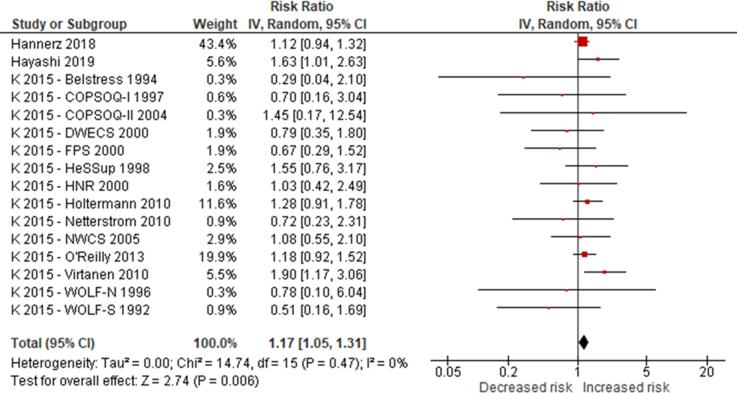
Main meta-analysis of cohort studies, Outcome: Died from ischaemic heart disease, Comparison: Worked ≥55 h/week compared with worked 35–40 h/week.

A total of 16 cohort studies with a total of 726,803 participants from two WHO regions reported estimates of the effect of exposure to long working hours on the risk of dying from IHD when working ≥55 h/week, compared with 35–40 h/week. These studies that we pooled in our meta-analysis were somewhat heterogeneous in that two studies defined the outcome as a fatal IHD event (Holtermann et al., 2010, O'Reilly and Rosato, 2013), whereas 14 of the studies defined the outcome as a non-fatal or fatal (or “mixed”) IHD event (Netterstrøm et al., 2010, Virtanen et al., 2010, Hannerz et al., 2018, Hayashi 2019¸WOLF-S, Belstress, WOLF-N, COPSOQ-I, HeSSup, FPS, HNR, DWECS, COPSOQ-II, NWCS). Applying the same criteria as in case of acquired IHD (Section 4.4.2.3), the heterogeneity of included studies was judged to be low. All these studies could consequently be included in a quantitative meta-analysis. We found that compared with working 35–40 h/week, working ≥55 h/week increased the risk of dying from IHD (RR 1.17, 95% CI 1.05 to 1.31, 16 studies, 726,803 participants, I^2^ 0%; Fig. 13).

### 4.5 Additional analyses

#### 4.5.1 Subgroup analyses

Subgroup analyses were performed for data from the main meta-analysis (cohort studies) with comparison between the group worked ≥55 h/week and the group worked 35–40 h/week. These analyses include subgrouping by WHO region, sex, and SES (Table 6). These subgroup analyses found no evidence for meaningful subgroup differences by WHO and sex, but persons with lower SES may have been at higher risk of acquiring and dying from IHD with a potential dose–response relationship observed (Table 6). The forest plots and results of additional subgroup analyses are presented in Appendix 7 in the Supplementary data.

**Table 6:** Summary of results from subgroup analyses on long working hours and ischaemic heart disease, cohort studies.

Acquired ischaemic heart disease (IHD) (IHD incidence)	Died from IHD (IHD mortality)
**WHO region** p = 0.89	**WHO region** p = 0.16
Americas 1.12 (0.92 to 1.37)	Americas –
Europe 1.13 (0.99 to 1.28)	Europe 1.15 (1.02 to 1.29)
Western Pacific 1.30 (0.73 to 2.33)	Western Pacific 1.63 (1.01 to 2.63)
**Sex** p = 0.99	**Sex** p = 0.99
Men 1.20 (0.80 to 1.82)	Men 1.21 (0.97 to 1.52)
Women 1.21 (077 to 1.91)	Women 1.21 (077 to 1.91)
**SES** p = 0.05	**SES** p = 0.05
High SES 0.94 (0.72 to 1.21)	High SES 0.94 (0.72 to 1.21)
Intermediate SES 1.10 (0.78 to 1.55)	Intermediate SES 1.10 (0.78 to 1.55)
Low SES 1.43 (1.14 to 1.79)	Low SES 1.43 (1.14 to 1.79)

#### 4.5.2 Sensitivity analyses

Sensitivity analyses were also performed for data from the main meta-analysis (cohort studies) with comparison between the group worked ≥55 h/week and the group worked 35–40 h/week. There were no meaningful differences by outcome measurement and by risk of bias (Table 7; Appendix 8 in the Supplementary data). However, studies with any “high”/”probably high” risk of bias in one or more domains may perhaps have reported somewhat more elevated risks than studies with “low”/”probably low” risk of bias in all domains for both outcomes (Table 7), with studies with “high”/”probably high” risk of selection bias increasing the effect estimates relatively more than studies with “high”/”probably high” risk of selection bias (footnotes of Table 7).

**Table 7:** Summary of results from sensitivity analyses on long working hours and ischaemic heart disease, cohort studies.

Acquired ischaemic heart disease (IHD) (IHD incidence)	Died from ischaemic heart disease (IHD mortality)
**Outcome measurement** p = 0.68	**Outcome measurement**
Health records 1.16 (1.01 to 1.32)	–
Self-reports 1.10 (0.92 to 1.32)	–
**Risk of bias** p = 0.37	**Risk of bias** p = 0.13
Any “high”/”probably high” 1.20 (1.01 to 1.41)	Any “high”/”probably high” 1.45 (1.06 to 1.99)
Only “low”/”probably low” 1.08 (0.93 to 1.25)	Only “low”/”probably low” 1.12 (0.99 to 1.26)

Footnotes: Sensitivity analysis for IHD incidence by risk of selection bias (p = 0.01).

“high”/”probably high” 1.53 (1.19 to 1.96).

“low”/”probably low” 1.07 (0.96 to 1.19).

Sensitivity analysis for IHD incidence by risk of detection bias (outcome assessment) (p = 0.64).

“high”/”probably high” 1.09 (0.88 to 1.35).

“low”/”probably low” 1.16 (1.03 to 1.31).

### 4.6 Quality of evidence

#### 4.6.1 Outcome: Acquired ischaemic heart disease (IHD incidence)

##### 4.6.1.1 Comparison: Worked 41–48 h/week, compared with worked 35–40 h/week

We did not have any serious concerns regarding risk of bias in the body of evidence on this comparison for this outcome, because we judged the risk of bias to be probably low, and therefore the quality of evidence was not downgraded for this consideration (+/- 0 levels). We also did not have any serious concerns regarding inconsistency, specifically regarding the cohort studies that were judged to be of higher quality. Therefore, no downgrading of the quality of evidence (+/- 0 levels) was done. We did not have serious concerns for indirectness, regarding the combination of the outcome definition including “mixed” (fatal or non-fatal) events and non-fatal events. Our exploratory subgroup analyses did not indicate any difference between “mixed” events and non-fatal events (Appendix 6 in the Supplementary data), and therefore the quality of evidence was not downgraded for this consideration (+/- 0 levels). We had serious concerns for imprecision, given large CIs in several studies, and we therefore downgraded by one level (-1). We did not have any serious concerns for publication bias (+/- 0 levels). We upgraded neither for a large effect estimate, nor for evidence for a dose–response. In conclusion, we started at “moderate” for observational studies and downgraded by one level (-1) for imprecision to a final rating of “low”.

##### 4.6.1.2 Comparison: Worked 49–54 h/week, compared with worked 35–40 h/week

We did not have any serious concerns regarding risk of bias in the body of evidence on this comparison for this outcome, because we judged the risk of bias to be probably low, and we therefore did not downgrade the quality of evidence for this consideration (+/- 0 levels). We also did not have any serious concerns regarding inconsistency, specifically regarding the cohort studies that were judged to be of higher quality. Therefore, no downgrading of the quality of evidence (+/- 0 levels) was done. We did not have serious concerns for indirectness, regarding the combination of the outcome definition including “mixed” (fatal or non-fatal) events and non-fatal events. Our sensitivity analyses did not indicate any difference between “mixed” events and non-fatal events (Appendix 6 in the Supplementary data), and we therefore did not downgrade the quality of evidence for this consideration (+/- 0 levels). We had serious concerns for imprecision, given large CIs in several studies, and we therefore downgraded by one level (−1). We did not have any serious concerns for publication bias (+/- 0 levels). We upgraded neither for a large effect estimate, nor for evidence for a dose–response. In summary, we started at “moderate” for observational studies and downgraded by one level (−1) for imprecision to a final rating of “low”.

##### 4.6.1.3 Comparison: Worked ≥55 h/week, compared with worked 35–40 h/week

**Fig. 14:**
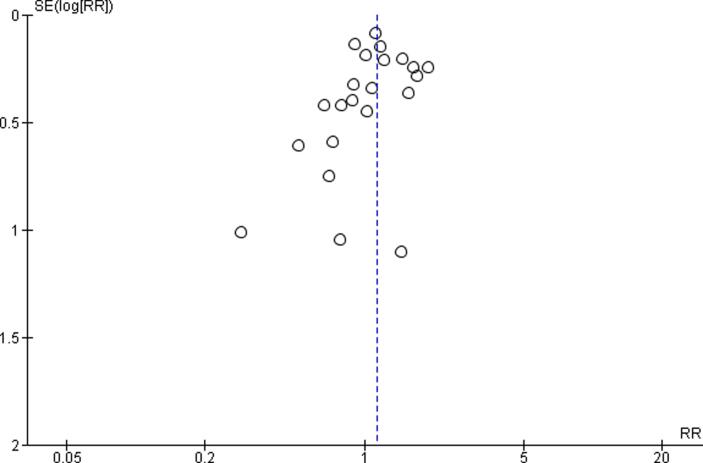
Funnel plot or prioritized evidence (cohort studies), Outcome: Acquired ischaemic heart disease, Comparison: Worked ≥55 h/week compared with worked 35–40 h/week.

We did not have any serious concerns regarding risk of bias in the body of evidence on this comparison for this outcome, because the risk of bias was judged to be probably low, and we therefore did not downgrade the quality of evidence for this consideration (+/- 0 levels). We also did not have any serious concerns regarding inconsistency, specifically regarding the cohort studies that were judged to be of higher quality. Therefore, no downgrading of the quality of evidence (+/- 0 levels) was done. We did not have serious concerns for indirectness, regarding the combination of the outcome definition including “mixed” (fatal or non-fatal) events and non-fatal events. Our sensitivity analyses did not indicate any difference between “mixed” events and non-fatal events (Appendix 6 in the Supplementary data), and therefore the quality of evidence was not downgraded for this consideration (+/- 0 levels). We had no serious concerns for imprecision, given relatively narrow CIs in most studies, and we therefore did not downgrade (+/- 0 levels). We did not have any serious concerns for publication bias (+/- 0 levels) (see Fig. 14). We upgraded neither for a large effect estimate, nor for evidence for a dose–response. In summary, we started at “moderate” for observational studies and did not down- or upgrade, and therefore arrived at the final rating of “moderate”.

#### 4.6.2 Outcome: Died from ischaemic heart disease (mortality of IHD)

##### 4.6.2.1 Comparison: Worked 41–48 h/week, compared with worked 35–40 h/week

We did not have any serious concerns regarding risk of bias in the body of evidence on this comparison for this outcome, because we judged the risk of bias to be probably low, and therefore the quality of evidence was not downgraded for this consideration (+/- 0 levels). We also did not have any serious concerns regarding inconsistency, specifically with regard to the cohort studies that were judged to be of higher quality. Therefore, no downgrading of the quality of evidence (+/- 0 levels) was done. We had serious concerns for indirectness, because the outcome definition included “mixed” (fatal or non-fatal) events only (rather than fatal events only), and we could not conduct sensitivity analyses to test for differences between mixed events and fatal events, and therefore the quality of evidence was downgraded by one level (−1). We also had serious concerns for imprecision, given large CIs in several studies, and we therefore downgraded by one level (−1). We did not have any serious concerns for publication bias (+/- 0 levels). We upgraded neither for a large effect estimate, nor for evidence for a dose-response. In summary, we started at “moderate” for observational studies and downgraded by one level (−1) for imprecision to a final rating of “low”.

##### 4.6.2.2 Comparison: Worked 49–54 h/week, compared with worked 35–40 h/week

We did not have any serious concerns regarding risk of bias in the body of evidence on this comparison for this outcome, because we judged the risk of bias to be probably low, and we therefore did not downgrade the quality of evidence for this consideration (+/- 0 levels). We also did not have any serious concerns regarding inconsistency, specifically with regard to the cohort studies that were judged to be of higher quality. Therefore, no downgrading of the quality of evidence (+/- 0 levels) was done. We had serious concerns for indirectness, because the outcome definition included “mixed” (fatal or non-fatal) events only (rather than fatal events only), and we could not conduct sensitivity analyses to test for differences between mixed events and fatal events, and therefore the quality of evidence was downgraded by one level (-1). We also had serious concerns for imprecision, given large CIs in several studies, and we therefore downgraded by one level (-1) We did not have any serious concerns for publication bias (+/- 0 levels). We upgraded neither for a large effect estimate, nor for evidence for a dose–response. In summary, we started at “moderate” for observational studies and downgraded by one level (-1) for imprecision to a final rating of “low”.

##### 4.6.2.3 Comparison: Worked ≥55 h/week, compared with worked 35–40 h/week

**Fig. 15:**
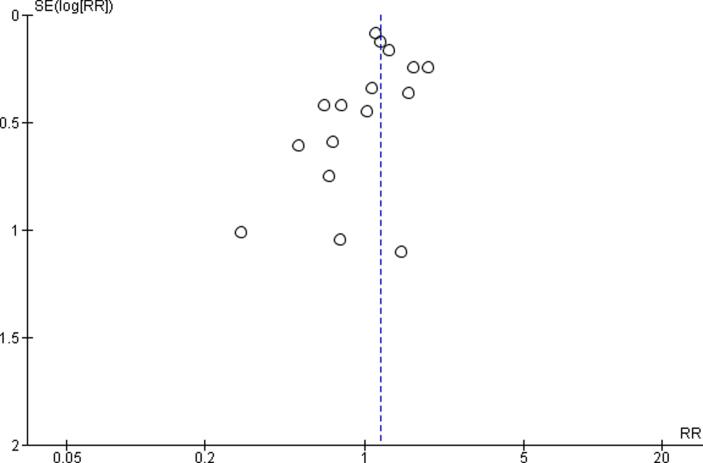
Funnel plot of prioritized evidence (cohort studies), Outcome: Died from ischaemic heart disease, Comparison: Worked ≥55 h/week compared with worked 35–40 h/week.

We did not have any serious concerns regarding risk of bias in the body of evidence on this comparison for this outcome, because we judged the risk of bias to be probably low, and we therefore did not downgrade the quality of evidence for this consideration (+/- 0 levels). We also did not have any serious concerns regarding inconsistency, specifically with regard to the cohort studies that were judged to be of higher quality. Therefore, no downgrading of the quality of evidence (+/- 0 levels) was done. We did not have serious concerns for indirectness, regarding the combination of the outcome definition including “mixed” (fatal or non-fatal) events and non-fatal events. Our sensitivity analyses did not indicate any difference between “mixed” events and non-fatal events (Appendix 6 in the Supplementary data), and we therefore did not downgrade the quality of evidence for this consideration (+/- 0 levels). We had no serious concerns for imprecision, given relatively narrow CIs in a majority of studies, and we therefore did not downgrade (+/- 0 levels). We did not have any serious concerns for publication bias (+/- 0 levels) (see Fig. 15). We upgraded neither for a large effect estimate, nor for evidence for a dose-response. In summary, we started at “moderate” for observational studies and did not down- or upgrade, and therefore arrived at the final rating of “moderate”.

### 4.7 Assessment of strength of evidence

According to our protocol we rated the strength of evidence based on a combination of four criteria outlined in the Navigation guide: (1) Quality of the entire body of evidence; (2) Direction of the effect estimate; (3) Confidence in the effect estimate; (4) Other compelling attributes.

#### 4.7.1 Quality of the entire body of evidence

Concerning the number, size, and quality of individual studies, the body of evidence is sufficient to assess the toxicity/harmfulness of the exposure. The meta-analyses based on 26 cohort studies in total, conducted in different regions, including a very large number of participants, and taking into account relevant confounders, documents a moderately increased risk of incident non-fatal and/or fatal IHD when working ≥55 h/week compared with 35–40 h/week, with the lower CI beyond 1.0 and a rather narrow overall CI. This estimate is similar, whether fatal or non-fatal events, or combined (“mixed”) events, enter the analysis. The quality of studies is adequate, given similar study protocols, consistent measurement of exposure and outcome, and clear temporal distinction between exposure and outcome, including control of reverse causation by excluding studies with proximal outcomes to exposure assessment. Overall, risk of bias of these cohort studies is low or probably low, thus supporting adequate quality. We did not consider the evidence of case-control studies in our assessment of quality and strength of evidence, giver the lower confidence we have in this study design.

#### 4.7.2 Direction of the effect estimate

The study results are sufficient to assess the direction of the effect estimate. For all three exposure categories (41–48 h/week; 49–54 h/week; ≥55 h/week) no single study documented a negative effect estimate (with the higher CI below 1.0). In the first two exposure categories, all studies except one displayed effect estimates around 1.0, and in the third exposure category five studies demonstrated positive effect estimates, with lower CIs beyond, or close to 1.0. These latter studies with a weight of over 60% accounted for an acceptable consistency of findings, both regarding fatal and non-fatal (or “mixed”) outcomes. Overall, heterogeneity was low.

#### 4.7.3 Confidence in the effect estimate

There is limited evidence to determine the level of confidence in the effect estimate, at least for the following reasons. First, while studies include the test of several relevant confounders that in part can also act as mediators, no additional data are available on causal pathways linking exposure to the health outcome under study. Indirect supportive evidence comes from studies dealing with health-adverse working conditions other than long working hours, but conditions that implicate identical pathways from exposure to outcome, such as adverse health behaviours or chronic psychosocial stress with pathophysiological effects on IHD. Second, the assumption of a dose–response relationship between the three exposure categories and the outcome was difficult to determine from our findings. There was no indication of an effect at the lowest exposure category and perhaps a slightly larger effect at the next lowest exposure category. An effect estimate with the lower CI above 1 was found at the third exposure category, ≥55 h/week. There could be a threshold, but this is difficult to ascertain from the currently available evidence. Third, the magnitude of the effect estimate was modest, given an overall pooled RR with a 95% CI of between 1.13 and 1.17. Although even a modest increase in risk can be relevant for policy under conditions of high prevalence of the exposure (which is certainly the case with long working hours), this low magnitude of the estimated effect does not increase our confidence in the effect estimate. Fourth, no intervention studies are available that demonstrate a reduction of the effect estimate because of reducing the exposure to minimal level. However, studies with the comparison “Worked any overtime compared with worked no overtime” could perhaps be seen as approximations of intervention studies, and the two studies with this comparison that we included in our systematic review and a meta-analysis for the outcome “Acquired IHD” found that working any overtime led to a large increase in the risk of the outcome (pooled OR 1.97, 95% CI 1.30 to 3.00, 2 studies, I^2^ 12%; see Fig. 10).

#### 4.7.4 Other compelling attributes

We were not able to access data that could offer evidence for a discussion of other compelling attributes in assessing the strength of evidence. In summary, we conclude that there is limited evidence of the toxicity of long working hours, defined as ≥55 h/week, for elevated risk of fatal or non-fatal IHD.

Additional assessment of strength of evidence based on the Bradford Hill criteria is on Appendix 9 in the Supplementary data (though note that this is already covered via our approach to evaluating the quality of evidence as described above) (Dragano et al., 2017, Kivimaki et al., 2012, Theorell et al., 2016).

#### 4.7.5 Rating by outcome and comparison

Based on the considerations presented above, we judged the existing bodies of evidence as:

• Inadequate evidence for harmfulness for the exposure categories 41–48 and 49–54 h/week for IHD prevalence, incidence and mortality and for the exposure category ≥55 h/week for IHD prevalence.
• Sufficient evidence for harmfulness for the exposure categories ≥55 h/week for IHD incidence and mortality.

## 5 Discussion

### 5.1 Summary of evidence

As shown in the table of summary of findings (Table 8), our systematic review found no eligible study on the outcome of IHD prevalence. It found low quality of evidence of weak or no associations between the exposure categories of working 41–48 h/week and working 49–54 h/week and the outcomes of IHD incidence and mortality, when compared to 35–40 h/week. Based on the other considerations for evaluating the strength of evidence we concluded that there was inadequate evidence of toxicity based on human evidence. We found moderate quality evidence of clinically meaningful associations of working ≥55 h/week with elevated risk of acquired or died from IHD and concluded there is sufficient evidence of toxicity from the human evidence. Particularly, findings based on 24 cohort studies documented modest, but relatively robust effects of working ≥55 h/week on risk of non-fatal and fatal IHD, given the large sample size, the standardized adjustment for confounding, and the probably low risk of bias on most domains. A risk elevation by 13–17 percent is considered modest, but in view of the high prevalence of long working hours and considerable incidence/mortality rates of IHD in working populations, this risk deserves attention in terms of preventive occupational health measures. Overall, the heterogeneity of findings is low, and sensitivity analyses confirm the robustness of results.

**Table 8:** Summary of findings.

Effect of exposure to long working hours on ischemic heart disease among workers								
Population: all ≥15 years workers Settings: all countries and work settings Exposure: worked 41–48, 49–54 or ≥55 h/week Comparator: worked 35–40 h/week								
Outcomes	Exposure category	Illustrative comparative risks (95% CI)		Relative effect (95% CI)	No. of participants (studies)	Quality of the evidence	Strength of evidence for human evidence	Comments
		Assumed risk Unexposed workers (worked 35–40 h/week)	Corresponding risk Workers in the exposure category					
**Has ischaemic heart disease**	**–**	**–**	**–**	**–**	–	–	−	No evidence was found on this outcome.
**Acquired ischaemic heart disease** (measured with administrative record or self-report) Follow-up: 1–20 years	**Worked 41**–**48 h/week**	**150 cases per 100,000 person years**^a^	**147 per 100,000 person years** (137 to161)	**RR 0.98** (0.91 to 1.07)	312,209 (20 studies)	⊕⊝⊝ Low^b^	Inadequate evidence of toxicity/harmfulness	Better indicated by lower values Additional evidence from nine case-control studies also provided no evidence for an effect for this comparison on this outcome. We are very uncertain about the effect of this exposure category on this outcome.
	**Worked 49**–**54 h/week**		**158 per 100,000 person years** (141 to 176)	**RR 1.05** (0.94 to 1.17)	308,405 (18 studies)	⊕⊝⊝ Low^b^	Inadequate evidence of toxicity/harmfulness	Better indicated by lower values Additional evidence from eight case-control studies also provided no evidence for an effect for this comparison on this outcome. We are very uncertain about the effect of this exposure category on this outcome.
	**Worked** ≥**55 h/week**		**170 per 100,000 person years** (153 to 189)	**RR 1.13** (1.02 to 1.26)	339,680 (22 studies)	⊕⊕⊝ Moderate	Sufficient evidence of toxicity/harmfulness	Better indicated by lower values Additional evidence from 11 case-control studies also suggests a small increase in the risk for the outcome for this comparison. Compared with working 35–40 h/week, working ≥55 h/week may have led to an increase in having acquired ischemic heart disease.
**Died from ischemic heart disease (mortality)** (measured with administrative record) Follow-up: 8–30 years	**41**–**48 h/w**	**150 cases per 100,000 person years**^a^	**149 per 100,000 person years** (132 to168)	**RR 0.99** (0.88 to 1.12)	288,278 (13 studies)	⊕⊝⊝ Low^b^^,^^c^	Inadequate evidence of toxicity/harmfulness	Better indicated by lower values We are very uncertain about the effect of this exposure category on this outcome
	**49**–**54 h/w**		**152 per 100,000 person years** (123 to 188)	**RR 1.01** (0.82 to 1.25)	284,474 (11 studies)	⊕⊝⊝ Low^b^^,^^c^	Inadequate evidence of toxicity/harmfulness	Better indicated by lower values. We are very uncertain about the effect of this exposure category on this outcome
	**≥55 h/w**		**176 per 10,000 person years** (158 to 196)	**RR 1.17** (1.05 to 1.31)	726,803 (16 studies)	⊕⊕⊝ Moderate	Sufficient evidence of toxicity/harmfulness	Better indicated by lower values Compared with working 35–40 h/week, working ≥55 h/week may have led to an increase in dying due to ischemic heart disease.
**CI**: confidence interval; **RR**: relative risk.								
Navigation Guide quality of evidence ratings **High quality:** Further research is very unlikely to change our confidence in the estimate of effect. **Moderate quality:** Further research is likely to have an important impact on our confidence in the estimate of effect and may change the estimate. **Low quality:** Further research is very likely to have an important impact on our confidence in the estimate of effect and is likely to change the estimate.								

a We extracted the risk among workers working 35–40 h/week from Hannerz et al. (2018) as the assumed risk. (Note that this study provided one baseline risk for both non-fatal and/or fatal ischemic heart disease events, so that it was not possible to differentiate assumed risk for exclusively non-fatal events and fatal events separately.)

b Downgraded by one grade, because of serious imprecision (i.e., large CIs in several included studies).

c Downgraded by one grades, because of serious indirectness (i.e., outcome definition included “mixed” (fatal and non-fatal) events, and no sensitivity analysis could be conducted to test for differences between mixed events and fatal and non-fatal events, respectively).

### 5.2 Comparison to previous systematic review evidence

Five previous systematic reviews and meta-analyses (Kivimaki et al., 2015, Virtanen et al., 2012, Virtanen and Kivimaki, 2018, Kang et al., 2012, Wong et al., 2019) have lent support to the notion that long working hours are associated with a modestly increased risk of incident fatal or non-fatal IHD. Our analysis corroborates this evidence. Considering the differences between previously published comprehensive systematic reviews and the current analysis, the following facts deserve attention.

First, two of the previous systematic reviews and meta-analyses (Kang et al., 2012, Wong et al., 2019) did not carefully define the exposure and outcome, any long working hours without dose differentiation and any cardiovascular disease including both heart disease and stroke were included. In addition, studies with different research designs were analysed together.

Second, with a focus on coronary heart disease, the evidence resulting from the Virtanen et al., meta-analysis (2012) revealed a number of relevant limitations, such as divergence in the assessment of the exposure, limited number of studies included (N = 12), limited statistical control of relevant confounding factors (e.g., age, sex, and SES) and of reverse causation. In contrast, our meta-analysis included a consistent definition of categories of long working hours, identified a larger number of studies from different WHO regions, and adjusted all analyses for the effects of relevant confounders (at least age, sex, and SES). The risk of bias due to reverse causation in most cohort studies was reduced by excluding participants with IHD at baseline.

Third, the Kivimaki et al., meta-analysis (2015) represents the most comprehensive systematic review on this topic up to the year of its publication. Major strengths are the inclusion of published and unpublished studies (thus addressing publication bias), the analysis of reverse causation, the test of a dose–response relationship between long working hours and IHD, and selected approaches towards subgroup analyses. These strengths were also met by the current study (see protocol paper Li et al., 2018), but additional strengths of the current study are identified below.

Fourth, the findings of the Virtanen and Kivimaki meta-analysis (2018) are largely identical with their previous review, adding one newly published report from Denmark (Hannerz et al., 2018), without substantially altering the overall effect estimate. Again, findings include IHD and stroke as separate health outcomes, whereas our systematic review focuses exclusively on IHD. While the empirical basis of the Virtanen and Kivimaki paper (2018) is only marginally different from their previous report, it includes an extensive discussion of potential mechanisms linking long working hours to cardiovascular disease. Being compared with these two recent comprehensive publications, our systematic review and meta-analysis documents the following additional strengths. First, we extended the number of included cohort studies by including one recently published study (Hayashi et al., 2019). Second, we extended the types of eligible study designs by including case-control studies and other non-randomized intervention studies. Third, we conducted subgroup analyses to strengthen the quality of evidence, for instance, variations of associations between long working hours and IHD according to WHO region, sex, age and SES were analysed. Finally, and importantly, none of the previous systematic reviews and meta-analyses distinguish non-fatal IHD events from fatal events, usually both types of events were mixed. However, burden of disease estimation requires evidence separately on incidence (non-fatal IHD events) and mortality (fatal IHD events). In this current report, we conducted analyses for non-fatal events and fatal events, respectively, with sensitivity analyses to compare non-fatal events with mixed events, or fatal events with mixed events. In summary, although our review built on the important work of previous systematic reviews including the 2015 Kivimaki et al systematic review and its 2018 update, our review further updates, extends and differentiates the existing body of systematic review evidence.

### 5.3 Limitations and strengths of this systematic review

#### 5.3.1 Limitations

Our systematic review has several limitations. First, while we conducted a broad and sensitive search, we may have missed eligible studies, for example, due to them being published in languages other than English. However, we searched many academic and grey literature databases using a comprehensive search strategy and consulted additional experts who also did not identify any additional eligible studies. Considering the large number of included studies, the size of participants and the number of disease events, it seems unlikely that the overall results would have been affected by this fact.

Second, we did not receive a substantial amount of the missing data we requested for the studies included in this systematic review. We requested missing data from principal study authors at least three times, but the principal study authors generally did not share these requested missing data with us or only shared selected data. As a result, we can only present limited evidence in this systematic review on:

• The subgroup analyses by age, sex, occupation and industrial sector.
• Dose-response associations between the different exposure categories and the outcomes (statistical testing was not possible).

In some cases, we know that the missing data requested were readily available to the principal study authors, but these requested missing data were nevertheless not shared with us. This has introduced some uncertainties in the evidence that could have been resolved had the principal study authors shared the requested missing data.

Third, the validity of exposure assessment was somehow restricted, not only due to lack of objective measurements, but also since exposure to long working hours was assessed at baseline only, thus preventing the analysis of potential changes of exposure over time. Moreover, importantly, given the purely quantitative assessment of long working hours, as well as the restriction to people’s first job, additional data on the potentially ‘toxic/harmful’ effects of long working hours were missed. This lack of contextual, qualitative data is considered a serious limitation of the current state of research in this field, not just of this review. For instance, in a recent study, a significantly increased IHD risk associated with overtime work was restricted to the group without financial reward or free time offered as compensation, whereas the group with rewarded overtime work did not exhibit an elevated risk, compared to those working standard time (Li and Siegrist, 2018).

#### 5.3.2 Strengths

Our systematic review and meta-analysis have a number of strengths, including:

• Strictly speaking, previous systematic reviews have not undergone all steps of systematic review (see Fig. 1 in (Woodruff and Sutton, 2014)), but our systematic review and meta-analysis have done so, including having pre-published a protocol and assessed strength of evidence, and this presents a substantial improvement in systematic review methods on the topic.
• Previous systematic reviews have not sought to differentiate IHD prevalence from IHD incidence (i.e., non-fatal events) and IHD mortality (fatal events), but our systematic review improves accuracy by differentiating these three different outcomes.
• Previous systematic reviews have not commonly and not comprehensively provided detailed analyses across all analytic steps of the systematic review and meta-analysis for comparisons of standard categories of exposure to long working hours compared with standard working hours. However, we have provided such analyses for three such comparisons commonly used in the epidemiological literature across all steps of the systematic review, and again this provides an improvement in accuracy of systematic review evidence on this topic.
• Whereas previous systematic review evidence has not commonly and comprehensively assessed risk of bias and quality of evidence using established systematic review frameworks with dedicated tools and approaches, we have rigorously applied the Navigation Guide framework in this systematic review, which should have ensured rigor and transparency in this systematic review.
• In previous systematic reviews, strength of the evidence was not commonly assessed, but in our systematic review we have applied pre-specified criteria to rate the strength of evidence for each comparison for each included outcome. This is a novel contribution to the systematic review and meta-analytic body of evidence on the topic.
• Finally, to our knowledge, this is the first systematic review and meta-analysis conducted specifically for a global occupational burden of disease study, and, as such, it provides a model for future systematic reviews that will help ensure that these global health estimates adhere fully with the *GATHER Guidelines for Accurate and Transparent Health Estimates Reporting* (Stevens et al., 2016).

## 6 Use of evidence for burden of disease estimation

This systematic review and meta-analysis was conducted by WHO and ILO, supported by a large network of experts, for the development of the WHO/ILO Joint Estimates, as part of the WHO/ILO Work-related Burden of Disease and Injury Study (Ryder, 2017). More specifically, it provides the crucial evidence base for the organizations to consider producing estimates of the burden of deaths and DALYs from IHD attributable to exposure to long working hours. The systematic review found large bodies of evidence from several prospective cohort studies for comparison of the exposure category ≥55 working hours/week to the category 35–40 working hours/week for the outcomes of IHD incidence and mortality. These bodies of evidence were judged to be of moderate quality and to provide sufficient evidence for toxicity/harmfulness. Producing estimates of the burden for IHD attributable to exposure to the category of working ≥55 working hours/week appears evidence-based and warranted, and the parameters reviewed (including the pooled RRs from the meta-analyses for these comparisons) appear suitable as input data for WHO/ILO modelling of work-related burden of disease and injury.

## 7 Conclusions

We judged the existing bodies of evidence as inadequate evidence for harmfulness for the exposure categories 41–48 and 49–54 h/week for IHD prevalence, incidence and mortality, and for the exposure category ≥55 h/week for IHD prevalence. Evidence on exposure to working ≥55 h/week was judged as sufficient evidence of harmfulness for IHD incidence and mortality. The RRs for the comparisons ≥55 h/week compared with 35–40 h/week are suitable as input data for WHO/ILO modelling of work-related burden of disease and injury.

## 8 Differences between protocol and systematic review

• Our protocol did not specify how to deal with studies with outcomes definitions being “mixed”, in terms of including both fatal and non-fatal events. We added such criteria for dealing with these studies with the outcome definition being “mixed”.
• The search strategy published in our protocol did not incorporate a recently developed strategy based on analytical text mining (Hausner et al., 2016, Stansfield et al., 2017) that was shown to be highly efficient. We therefore initially adopted the two strategies (protocol and analytical text mining) in parallel, using the following tools for text mining: Voyant (https://voyant-tools.org), PubReMiner (https://hgserver2.amc.nl/cgi-bin/miner/miner2.cgi) and Yale MeSH Analyzer (http://mesh.med.yale.edu). We then observed a substantially higher ability of identifying relevant studies by the latter strategy, based on 34 included studies of two previously published systematic reviews (Kivimaki et al., 2015, Virtanen et al., 2012).
• Our protocol said that we would search the Embase database, but since we did not have access to this database, the Scopus database was searched instead, which includes all records from Embase.
• In the protocol, we planned to convert OR into RR, if possible. To conduct conversion, information on “prevalence of outcome in reference group or baseline risk” is required. However, such information was not available from any included studies. For case-control studies, ORs were reported and were synthesized directly. For cohort studies, ORs, HRs, and RRs were reported and were used for meta-analyses without any conversion, in line with an earlier systematic review and meta-analysis on this topic [(Kivimaki et al., 2015, Virtanen et al., 2012), Page 1741: “Because disease incidence was low in the cohort studies, we regarded ORs as close approximations of RR and combined them with HRs, resulting in a common estimate of RR.” Supplementary appendix Page 11: “Our sensitivity analyses also showed that the pooled relative risk for the association between long working hours and coronary heart disease is unchanged if study-specific odds ratios (pooled relative risk 1.04, 95% CI 0.79–1.37, p = 0.78) are used instead of study-specific hazard ratios (pooled relative risk 1.05, 95% CI 0.80–1.38, p = 0.72) (eFigure 8).”].

## 9 Financial support

All authors are salaried staff members of their respective institutions. The publication was prepared with financial support from the 10.13039/100004423World Health Organization cooperative agreement with the 10.13039/100000125Centres for Disease Control and Prevention National Institute for Occupational Safety and Health of the United States of America (Grant 1E11OH0010676-02; Grant 6NE11OH010461-02-01; and Grant 5NE11OH010461-03-00).

## 10 Sponsors

The sponsors of this systematic review are the World Health Organization and the International Labour Organization.

## Author contributions

Had the idea for the systematic review: FP, Ivan Ivanov (WHO), Nancy Leppink (ILO).

Coordinated the entire series of systematic reviews: FP, YU.

Selected the lead reviewers and gathered the review teams: FP, Ivan Ivanov (WHO), Nancy Leppink (ILO).

Were the lead reviewers of this systematic review: JL, JS.

Led the design of the systematic review including developed the standard methods: FP.

Contributed substantially to the design of the systematic review: JL, JS, YU, AD, LG, SI, DVP, RR, GS.

Conducted the search: MIM, JL, BR.

Selected studies: JS, JL, MIM.

Extracted data: AT, MMF, MR, JS, JL.

Requested missing data: JL, JS, AD, LG, RR.

Assessed risk of bias: RR, EC, JS, JL, DVP, AD, BR.

Conducted the meta-analyses: JL, FP, JS.

Assessed quality of evidence: JL, JS.

Assessed evidence on causality: JS, JL.

Developed the standards and wrote the template for all systematic reviews in the series: FP.

Wrote the first draft of the manuscript using the template: JL, JS.

Revising the manuscript critically for important intellectual content: FP, AD, GS, BR, RR, RLM, TJW.

Ensured tailoring of the systematic review for WHO/ILO estimation purposes: FP.

Ensured harmonization across systematic reviews in the series: FP.

Approved the final version of the systematic review to be published: All authors.

Agreed to be accountable for all aspects of the work in ensuring that questions related to the accuracy or integrity of any part of the work are appropriately investigated and resolved: All authors.

## Declaration of Competing Interest

The authors declare that they have no known competing financial interests or personal relationships that could have appeared to influence the work reported in this paper.

## References

[b0005] 104th International Labour Conference. Transition from the Informal to the Formal Economy (Recommendation No. 204). Geneva: International Labour Organization 2015.

[b0010] Anderson L.M., Petticrew M., Rehfuess E., Armstrong R., Ueffing E., Baker P., Francis D., Tugwell P. (2011). Using logic models to capture complexity in systematic reviews. Res.. Synth. Methods.

[b0015] Arditi C., Burnand B., Peytremann-Bridevaux I. (2016). Adding non-randomised studies to a Cochrane review brings complementary information for healthcare stakeholders: an augmented systematic review and meta-analysis. BMC Health Serv. Res..

[b0020] Balshem H., Helfand M., Schunemann H.J., Oxman A.D., Kunz R., Brozek J., Vist G.E., Falck-Ytter Y., Meerpohl J., Norris S., Guyatt G.H. (2011). GRADE guidelines: 3. Rating the quality of evidence. J. Clin. Epidemiol..

[b0025] Barroga E.F., Kojima T. (2013). Research study designs: an appraisal for peer reviewers and science editors. Eur. Sci. Ed..

[b0030] Beller, E.M., Glasziou, P.P., Altman, D.G., Hopewell, S., Bastian, H., Chalmers, I., Gotzsche, P.C., Lasserson, T., Tovey, D., Group, P.f.A., 2013. PRISMA for Abstracts: reporting systematic reviews in journal and conference abstracts. PLoS Med 10, e1001419. 10.1371/journal.pmed.1001419 PMC3621753 23585737

[b0035] Chandola T., Heraclides A., Kumari M. (2010). Psychophysiological biomarkers of workplace stressors. Neurosci. Biobehav. Rev..

[b0040] Cheng Y., Du C.L., Hwang J.J., Chen I.S., Chen M.F., Su T.C. (2014). Working hours, sleep duration and the risk of acute coronary heart disease: a case-control study of middle-aged men in Taiwan. Int. J. Cardiol..

[b0045] Deeks, J., Higgins, J., Altman, D., 2011. Chapter 9: Analysing data and undertaking meta-analyses in: Higgins J., Green S., eds. Cochrane Handbook for Systematic Reviews of Interventions Version 510 [updated March 2011] The Cochrane Collaboration, 2011 Available from wwwhandbookcochraneorg.

[b0050] Descatha A., Sembajwe G., Baer M., Boccuni F., Di Tecco C., Duret C., Evanoff B.A., Gagliardi D., Ivanov I.D., Leppink N., Magnusson Hanson L.L., Marinaccio A., Ozguler A., Pega F., Pico F., Prüss-Üstün A.M., Ronchetti M., Roquelaure Y., Sabbath E., Stevens G.A., Tsutsumi A., Ujita Y., Iavicoli S. (2018). WHO/ILO work-related burden of disease and injury: Protocol for systematic reviews of exposure to long working hours and of the effect of exposure to long working hours on stroke. Environ. Int..

[b0055] Dragano, N., Siegrist, J., Nyberg, S.T., Lunau, T., Fransson, E.I., Alfredsson, L., Bjorner, J.B., Borritz, M., Burr, H., Erbel, R., Fahlen, G., Goldberg, M., Hamer, M., Heikkila, K., Jockel, K.H., Knutsson, A., Madsen, I.E.H., Nielsen, M.L., Nordin, M., Oksanen, T., Pejtersen, J.H., Pentti, J., Rugulies, R., Salo, P., Schupp, J., Singh-Manoux, A., Steptoe, A., Theorell, T., Vahtera, J., Westerholm, P.J.M., Westerlund, H., Virtanen, M., Zins, M., Batty, G.D., Kivimaki, M., consortium, I.P.-W., 2107. Effort-reward imbalance at work and incident coronary heart disease: a multicohort study of 90,164 individuals. Epidemiology 28, 619–626. 10.1097/EDE.0000000000000666 PMC5457838 28570388

[b0060] Drazen J.M., de Leeuw P.W., Laine C., Mulrow C., DeAngelis C.D., Frizelle F.A., Godlee F., Haug C., Hebert P.C., James A., Kotzin S., Marusic A., Reyes H., Rosenberg J., Sahni P., Van der Weyden M.B., Zhaori G. (2010). Toward more uniform conflict disclosures: the updated ICMJE conflict of interest reporting form. JAMA.

[b0065] Drazen J.M., Van der Weyden M.B., Sahni P., Rosenberg J., Marusic A., Laine C., Kotzin S., Horton R., Hebert P.C., Haug C., Godlee F., Frizelle F.A., de Leeuw P.W., DeAngelis C.D. (2010). Uniform format for disclosure of competing interests in ICMJE journals. JAMA.

[b0070] Dulleck U., Schaffner M., Torgler B. (2014). Heartbeat and economic decisions: observing mental stress among proposers and responders in the ultimatum bargaining game. PLoS ONE.

[b0075] Ezzati M., Lopez A.D., Rodgers A., Murray C.J.L. (2004). Comparative Quantification of Health Risks: Global and Regional Burdedn of Disease Attributable to Selected Major Risk Factors ededs.

[b0080] Falger P.R., Schouten E.G. (1992). Exhaustion, psychological stressors in the work environment, and acute myocardial infarction in adult men. J. Psychosom. Res..

[b0085] Falk A., Kosse F., Menrath I., Verde P.E., Siegrist J. (2018). Unfair Pay and Health. Manage. Sci..

[b0090] Figueroa J.L. (2014). Distributional effects of Oportunidades on early child development. Soc. Sci. Med..

[b0095] Forsyth S.R., Odierna D.H., Krauth D., Bero L.A. (2014). Conflicts of interest and critiques of the use of systematic reviews in policymaking: an analysis of opinion articles. Syst. Rev..

[b0100] Fukuoka Y., Dracup K., Froelicher E.S., Ohno M., Hirayama H., Shiina H., Kobayashi F. (2005). Do Japanese workers who experience an acute myocardial infarction believe their prolonged working hours are a cause?. Int. J. Cardiol..

[b0105] Godderis L., Bakusic J., Boonen E., Delvaux E., Ivanov I.D., Lambrechts M.-C., Latorraca C.O., Leppink N., Martimbianco A.L., Pega F., Prüss-Üstün A.M., Riera R., Ujita Y., Pachito D.V. (2018). WHO/ILO work-related burden of disease and injury: protocol for systematic reviews of exposure to long working hours and of the effect of exposure to long working hours on alcohol use and alcohol use disorder. Environ. Int..

[b0110] Goodman J.E., Lynch H.N., Beck N.B. (2017). More clarity needed in the Navigation Guide systematic review framework. Environ. Int..

[b0115] Gunasekara F.I., Richardson K., Carter K., Blakely T. (2014). Fixed effects analysis of repeated measures data. Int. J. Epidemiol..

[b0120] Hannerz H., Larsen A.D., Garde A.H. (2018). Long weekly working hours and ischaemic heart disease: a follow-up study among 145 861 randomly selected workers in Denmark. BMJ Open.

[b0125] Hausner E., Guddat C., Hermanns T., Lampert U., Waffenschmidt S. (2016). Prospective comparison of search strategies for systematic reviews: an objective approach yielded higher sensitivity than a conceptual one. J. Clin. Epidemiol..

[b0130] Hayashi R., Iso H., Yamagishi K., Yatsuya H., Saito I., Kokubo Y., Eshak E.S., Sawada N., Tsugane S., Japan Public Health Center-Based Prospective Study, G. (2019). Working Hours and Risk of Acute Myocardial Infarction and Stroke Among Middle-Aged Japanese Men- The Japan Public Health Center-Based Prospective Study Cohort II. Circ. J..

[b0135] Higgins, J., Altman, D., Sterne, J., 2011. Chapter 8: Assessing risk of bias in included studies. In: Higgins J., Green S. (Eds.), Cochrane Handbook for Systematic Reviews of Interventions Version 510 [updated March 2011] The Cochrane Collaboration, 2011 Available from http://handbookcochraneorg.

[b0140] Higgins, J., Green, S., 2011. Cochrane Handbook for Systematic Reviews of Interventions Version 5.1.0 [updated March 2011]. The Cochrane Collaboration, 2011. Available from http://handbook.cochrane.org. ed^eds.

[b0145] Holtermann A., Mortensen O.S., Burr H., Sogaard K., Gyntelberg F., Suadicani P. (2010). Long work hours and physical fitness: 30-year risk of ischaemic heart disease and all-cause mortality among middle-aged Caucasian men. Heart.

[b0150] Hulshof C.T.J., Colosio C., Daams J.G., Ivanov I.D., Prakash K.C., Kuijer P., Leppink N., Mandic-Rajcevic S., Masci F., van der Molen H.F., Neupane S., Nygard C.H., Oakman J., Pega F., Proper K., Pruss-Ustun A.M., Ujita Y., Frings-Dresen M.H.W. (2019). WHO/ILO work-related burden of disease and injury: protocol for systematic reviews of exposure to occupational ergonomic risk factors and of the effect of exposure to occupational ergonomic risk factors on osteoarthritis of hip or knee and selected other musculoskeletal diseases. Environ. Int..

[b0155] Imai T., Kuwahara K., Miyamoto T., Okazaki H., Nishihara A., Kabe I., Mizoue T., Dohi S., Japan Epidemiology Collaboration on Occupational Health Study, G. (2016). Validity and reproducibility of self-reported working hours among Japanese male employees. J. Occup. Health.

[b0160] International Labour Organization, 1999. ILO estimates over 1 million work-related fatalities each year. Geneva: International Labour Organization.

[b0165] International Labour Organization, 2014. Safety and health at work: a vision for sustainable prevention: XX World Congress on Safety and Health at Work 2014: Global Forum for Prevention, 24-27 August 2014, Frankfurt, Germany. Geneva: International Labour Organization.

[b0170] Jarczok M.N., Jarczok M., Mauss D., Koenig J., Li J., Herr R.M., Thayer J.F. (2013). Autonomic nervous system activity and workplace stressors–a systematic review. Neurosci. Biobehav. Rev..

[b0175] Jeong I., Rhie J., Kim I., Ryu I., Jung P.K., Park Y.S., Lim Y.S., Kim H.R., Park S.G., Im H.J., Lee M.Y., Won J.U. (2013). Working hours and cardiovascular disease in Korean workers: a case-control study. J. Occup. Health.

[b0180] Johnson P.I., Koustas E., Vesterinen H.M., Sutton P., Atchley D.S., Kim A.N., Campbell M., Donald J.M., Sen S., Bero L., Zeise L., Woodruff T.J. (2016). Application of the Navigation Guide systematic review methodology to the evidence for developmental and reproductive toxicity of triclosan. Environ. Int..

[b0185] Johnson P.I., Sutton P., Atchley D.S., Koustas E., Lam J., Sen S., Robinson K.A., Axelrad D.A., Woodruff T.J. (2014). The Navigation Guide - evidence-based medicine meets environmental health: systematic review of human evidence for PFOA effects on fetal growth. Environ. Health Perspect..

[b0190] Kang M.Y., Park H., Seo J.C., Kim D., Lim Y.H., Lim S., Cho S.H., Hong Y.C. (2012). Long working hours and cardiovascular disease: a meta-analysis of epidemiologic studies. J. Occup. Environ. Med..

[b0195] Kaplan J.R., Manuck S.B. (1994). Antiatherogenic effects of beta-adrenergic blocking agents: theoretical, experimental, and epidemiologic considerations. Am. Heart J..

[b0200] Kivimaki, M., Jokela, M., Nyberg, S.T., Singh-Manoux, A., Fransson, E.I., Alfredsson, L., Bjorner, J.B., Borritz, M., Burr, H., Casini, A., Clays, E., De Bacquer, D., Dragano, N., Erbel, R., Geuskens, G.A., Hamer, M., Hooftman, W.E., Houtman, I.L., Jockel, K.H., Kittel, F., Knutsson, A., Koskenvuo, M., Lunau, T., Madsen, I.E., Nielsen, M.L., Nordin, M., Oksanen, T., Pejtersen, J.H., Pentti, J., Rugulies, R., Salo, P., Shipley, M.J., Siegrist, J., Steptoe, A., Suominen, S.B., Theorell, T., Vahtera, J., Westerholm, P.J., Westerlund, H., O'Reilly, D., Kumari, M., Batty, G.D., Ferrie, J.E., Virtanen, M., Consortium, I.P.-W., 2015. Long working hours and risk of coronary heart disease and stroke: a systematic review and meta-analysis of published and unpublished data for 603,838 individuals. Lancet 386, 1739–1746. 10.1016/S0140-6736(15)60295-1 26298822

[b0205] Kivimaki, M., Nyberg, S.T., Batty, G.D., Fransson, E.I., Heikkila, K., Alfredsson, L., Bjorner, J.B., Borritz, M., Burr, H., Casini, A., Clays, E., De Bacquer, D., Dragano, N., Ferrie, J.E., Geuskens, G.A., Goldberg, M., Hamer, M., Hooftman, W.E., Houtman, I.L., Joensuu, M., Jokela, M., Kittel, F., Knutsson, A., Koskenvuo, M., Koskinen, A., Kouvonen, A., Kumari, M., Madsen, I.E., Marmot, M.G., Nielsen, M.L., Nordin, M., Oksanen, T., Pentti, J., Rugulies, R., Salo, P., Siegrist, J., Singh-Manoux, A., Suominen, S.B., Vaananen, A., Vahtera, J., Virtanen, M., Westerholm, P.J., Westerlund, H., Zins, M., Steptoe, A., Theorell, T., Consortium, I.P.-W., 2012. Job strain as a risk factor for coronary heart disease: a collaborative meta-analysis of individual participant data. Lancet 380, 1491–1497. 10.1016/S0140-6736(12)60994-5 PMC3486012 22981903

[b0210] Kivimaki M., Steptoe A. (2018). Effects of stress on the development and progression of cardiovascular disease. Nat. Rev. Cardiol..

[b0215] Koustas E., Lam J., Sutton P., Johnson P.I., Atchley D.S., Sen S., Robinson K.A., Axelrad D.A., Woodruff T.J. (2014). The Navigation Guide - evidence-based medicine meets environmental health: systematic review of nonhuman evidence for PFOA effects on fetal growth. Environ. Health Perspect..

[b0220] Lam, J., Koustas, E., Sutton, P., Cabana M., Whitaker E., Padula, A., Vesterinen, H., Daniels, N., Woodruff, T.J., 2016a. Applying the Navigation Guide: Case Study #6. Association Between Formaldehyde Exposures and Asthma (in preparation).

[b0225] Lam J., Koustas E., Sutton P., Johnson P.I., Atchley D.S., Sen S., Robinson K.A., Axelrad D.A., Woodruff T.J. (2014). The Navigation Guide - evidence-based medicine meets environmental health: integration of animal and human evidence for PFOA effects on fetal growth. Environ. Health Perspect..

[b0230] Lam J., Lanphear B., Bellinger D., Axelrad D., McPartland J., Sutton P., Davidson L.I., Daniels N., Sen S., Woodruff T.J. (2017). Developmental PBDE exposure and IQ/ADHD in childhood: a systematic review and meta-analysis. Environ. Health Perspect..

[b0235] Lam, J., Sutton, P., Halladay, A., Davidson, L.I., Lawler, C., Newschaffer, C.J., Kalkbrenner, A., Joseph J. Zilber School of Public Health, Windham, G.C., Daniels, N., Sen, S., Woodruff, T.J., 2016b. Applying the Navigation Guide Systematic Review Methodology Case Study #4: Association between Developmental Exposures to Ambient Air Pollution and Autism. PLoS One 21.

[b0240] Lam, J., Sutton, P., Padula, A.M., Cabana, M.D., Koustas, E., Vesterinen, H.M., Whitaker, E., Skalla, L., Daniels, N., Woodruff, T.J., 2016c. Applying the Navigation Guide Systematic Review Methodology Case Study #6: Association between Formaldehyde Exposure and Asthma: A Systematic Review of the Evidence: (Protocol registered in PROSPERO, CRD42016038766). San Francisco, CA: University of California at San Francisco.

[b0245] Lee S., McCann D., Messenger A.G. (2007). Working time around the world: Trends in Working Hours, Laws and Policies in a Global Comparative Perspective.

[b0250] Li J., Brisson C., Clays E., Ferrario M.M., Ivanov I.D., Landsbergis P., Leppink N., Pega F., Pikhart H., Prüss-Üstün A.M., Rugulies R., Schnall P.L., Stevens G.A., Tsutsumi A., Ujita Y., Siegrist J. (2018). WHO/ILO work-related burden of disease and injury: Protocol for systematic reviews of exposure to long working hours and of the effect of exposure to long working hours on ischaemic heart disease. Environ. Int..

[b0255] Li J., Siegrist J. (2018). The role of compensation in explaining harmful effects of overtime work on self-reported heart disease: preliminary evidence from a Germany prospective cohort study. Am. J. Ind. Med..

[b0260] Liberati A., Altman D.G., Tetzlaff J., Mulrow C., Gotzsche P.C., Ioannidis J.P., Clarke M., Devereaux P.J., Kleijnen J., Moher D. (2009). The PRISMA statement for reporting systematic reviews and meta-analyses of studies that evaluate health care interventions: explanation and elaboration. PLoS Med..

[b0265] Liu, Y., Tanaka, H., Fukuoka Heart Study, G., 2002. Overtime work, insufficient sleep, and risk of non-fatal acute myocardial infarction in Japanese men. Occup. Environ. Med. 59, 447–451. 10.1136/oem.59.7.447 PMC1740308 12107292

[b0270] Ma Y., Wang Y.J., Chen B.R., Shi H.J., Wang H., Khurwolah M.R., Li Y.F., Xie Z.Y., Yang Y., Wang L.S. (2017). Study on association of working hours and occupational physical activity with the occurrence of coronary heart disease in a Chinese population. PLoS ONE.

[b0275] Mandrioli D., Schlunssen V., Adam B., Cohen R.A., Colosio C., Chen W., Fischer A., Godderis L., Goen T., Ivanov I.D., Leppink N., Mandic-Rajcevic S., Masci F., Nemery B., Pega F., Pruss-Ustun A., Sgargi D., Ujita Y., van der Mierden S., Zungu M., Scheepers P.T.J. (2018). WHO/ILO work-related burden of disease and injury: Protocol for systematic reviews of occupational exposure to dusts and/or fibres and of the effect of occupational exposure to dusts and/or fibres on pneumoconiosis. Environ. Int..

[b0280] McEwen B.S. (1998). Protective and damaging effects of stress mediators. N. Engl. J. Med..

[b0285] McGwin, G., Jr., 2005. Extended work schedules and health outcomes in the U.S. NIOSH 2005 Feb: 1-80 (NIOSHTIC No.: 20028863).

[b0290] Mendis, S., Puska, P., Norrving, B. (Eds.), 2011. Global Atlas on cardiovascular disease prevention and control: Policies and interventions. World Health Organization, Geneva.

[b0295] Moher, D., Shamseer, L., Clarke, M., Ghersi, D., Liberati, A., Petticrew, M., Shekelle, P., Stewart, L.A., Group, P.-P., 2015. Preferred reporting items for systematic review and meta-analysis protocols (PRISMA-P) 2015 statement. Syst. Rev. 4, 1. 10.1186/2046-4053-4-1 PMC4320440 25554246

[b0300] Morgan R.L., Thayer K.A., Bero L., Bruce N., Falck-Ytter Y., Ghersi D., Guyatt G., Hooijmans C., Langendam M., Mandrioli D., Mustafa R.A., Rehfuess E.A., Rooney A.A., Shea B., Silbergeld E.K., Sutton P., Wolfe M.S., Woodruff T.J., Verbeek J.H., Holloway A.C., Santesso N., Schunemann H.J. (2016). GRADE: Assessing the quality of evidence in environmental and occupational health. Environ. Int..

[b0305] Morgan R.L., Whaley P., Thayer K.A., Schunemann H.J. (2018). Identifying the PECO: a framework for formulating good questions to explore the association of environmental and other exposures with health outcomes. Environ. Int..

[b0310] Muggah E., Graves E., Bennett C., Manuel D.G. (2013). Ascertainment of chronic diseases using population health data: a comparison of health administrative data and patient self-report. BMC Public Health.

[b0315] Murray, C.J.L., Ezzati, M., Lopez, A.D., Rodgers, A., Vander Hoorn, S., 2004. Comparative quantification of health risks: conceptual framework and methodological issues. In: Ezzati, M., Lopez, A.D., Rodgers, A., Murray, C.J.L. (Eds.), Comparative Quantification of Health Risks: Global and Regional Burdedn of Disease Attributable to Selected Major Risk Factors. World Health Organization, Geneva.

[b0320] Nakata A. (2012). Psychosocial job stress and immunity: a systematic review. Methods Mol. Biol..

[b0325] National Academies of Sciences, E., and Medicine, 2017. Application of Systematic Review Methods in an Overall Strategy for Evaluating Low-Dose Toxicity from Endocrine Active Chemicals. Washington (DC): National Academies Press (US). 28896009

[b0330] Netterstrøm B., Kristensen T.S., Jensen G., Schnor P. (2010). Is the demand-control model still a usefull tool to assess work-related psychosocial risk for ischemic heart disease? Results from 14 year follow up in the Copenhagen City Heart study. Int. J. Occup. Med. Environ. Health.

[b0335] O'Reilly D., Rosato M. (2013). Worked to death? A census-based longitudinal study of the relationship between the numbers of hours spent working and mortality risk. Int. J. Epidemiol..

[b0340] Paulo M.S., Adam B., Akagwu C., Akparibo I., Al-Rifai R.H., Bazrafshan S., Gobba F., Green A.C., Ivanov I., Kezic S., Leppink N., Loney T., Modenese A., Pega F., Peters C.E., Pruss-Ustun A.M., Tenkate T., Ujita Y., Wittlich M., John S.M. (2019). WHO/ILO work-related burden of disease and injury: protocol for systematic reviews of occupational exposure to solar ultraviolet radiation and of the effect of occupational exposure to solar ultraviolet radiation on melanoma and non-melanoma skin cancer. Environ. Int..

[b0345] Pega F., Blakely T., Glymour M.M., Carter K.N., Kawachi I. (2016). Using marginal structural modeling to estimate the cumulative impact of an unconditional tax credit on self-rated health. Am. J. Epidemiol..

[b0350] Pega F., Liu S.Y., Walter S., Lhachimi S.K. (2015). Unconditional cash transfers for assistance in humanitarian disasters: effect on use of health services and health outcomes in low- and middle-income countries. Cochrane Database System. Rev..

[b0355] Pega F., Liu S.Y., Walter S., Pabayo R., Saith R., Lhachimi S.K. (2017). Unconditional cash transfers for reducing poverty and vulnerabilities: effect on use of health services and health outcomes in low- and middle-income countries. Cochrane Database System. Rev..

[b0365] Pruss-Ustun A., Wolf J., Corvalan C., Bos R., Neira M. (2017). Preventing disease through healthy environments: a global assessment of the burden of disease from enviornmental risks. Department of Public Health, Environmental and Social Detereminants of Health.

[b0360] Pega F., Norris S., Backes C., Bero L., Descatha A., Gagliardi D., Godderis L., Loney T., Modenese A., Morgan R., Pachito D., Paulo M., Scheepers P., Schlünssen V., Sgargi D., Silbergeld E., Sørensen K., Sutton P., Tenkate T., Torreão D., Ujita Y., van Deventer E., Woodruff T., Mandrioli D. (2020). RoB-SPEO: A tool for assessing risk of bias in studies estimating the prevalence of exposure to occupational risk factors developed from the WHO/ILO Joint Estimates of the Work-related Burden of Disease and Injury. Environ. Int..

[b0370] Rehfuess E.A., Booth A., Brereton L., Burns J., Gerhardus A., Mozygemba K., Oortwijn W., Pfadenhauer L.M., Tummers M., van der Wilt G.J., Rohwer A. (2018). Towards a taxonomy of logic models in systematic reviews and health technology assessments: a priori, staged, and iterative approaches. Res. Synth. Methods.

[b0375] Rooney A.A., Cooper G.S., Jahnke G.D., Lam J., Morgan R.L., Boyles A.L., Ratcliffe J.M., Kraft A.D., Schunemann H.J., Schwingl P., Walker T.D., Thayer K.A., Lunn R.M. (2016). How credible are the study results? Evaluating and applying internal validity tools to literature-based assessments of environmental health hazards. Environ. Int..

[b0380] Rugulies R., Ando E., Ayuso-Mateos J.L., Bonafede M., Cabello M., Di Tecco C., Dragano N., Durand-Moreau Q., Eguchi H., Gao J., Garde A.H., Iavicoli S., Ivanov I.D., Leppink N., Madsen I.E.H., Pega F., Pruss-Ustun A.M., Rondinone B.M., Sorensen K., Tsuno K., Ujita Y., Zadow A. (2019). WHO/ILO work-related burden of disease and injury: protocol for systematic reviews of exposure to long working hours and of the effect of exposure to long working hours on depression. Environ. Int..

[b0385] Russek H.I., Zohman B.L. (1958). Relative significance of heredity, diet and occupational stress in coronary heart disease of young adults; based on an analysis of 100 patients between the ages of 25 and 40 years and a similar group of 100 normal control subjects. Am. J. Med. Sci..

[b0390] Ryder, G., 2017. Welcome address from the Director General of the International Labour Organization. XXI World Congress on Safety and Health at Work. Sands Expo and Convention Centre, Singapore.

[b0395] Schunemann H., Hill S., Guyatt G., Akl E.A., Ahmed F. (2011). The GRADE approach and Bradford Hill's criteria for causation. J. Epidemiol. Community Health.

[b0400] Schünemann, H., Oxman, A., Vist, G., Higgins, J., Deeks, J., Glasziou, P., Guyatt, G., 2011. Chapter 12: Interpreting results and drawing conclusions. In: Higgins, J., Green, S. (Eds.) Cochrane Handbook for Systematic Reviews of Interventions Version 510 [updated March 2011]. Available from www.handbook.cochrane.org: The Cochrane Collaboration.

[b0405] Shamseer L., Moher D., Clarke M., Ghersi D., Liberati A., Petticrew M., Shekelle P., Stewart L.A. (2015). Group, P.-P Preferred reporting items for systematic review and meta-analysis protocols (PRISMA-P) 2015: elaboration and explanation. BMJ.

[b0410] Sokejima S., Kagamimori S. (1998). Working hours as a risk factor for acute myocardial infarction in Japan: case-control study. BMJ.

[b0415] Sonnentag S., Venz L., Casper A. (2017). Advances in recovery research: What have we learned? What should be done next?. J. Occup. Health Psychol..

[b0420] Stansfield C., O'Mara-Eves A., Thomas J. (2017). Text mining for search term development in systematic reviewing: a discussion of some methods and challenges. Res. Synth. Methods.

[b0425] Stevens G.A., Alkema L., Black R.E., Boerma J.T., Collins G.S., Ezzati M., Grove J.T., Hogan D.R., Hogan M.C., Horton R., Lawn J.E., Marusic A., Mathers C.D., Murray C.J., Rudan I., Salomon J.A., Simpson P.J., Vos T., Welch V. (2016). Guidelines for Accurate and Transparent Health Estimates Reporting: the GATHER statement. Lancet.

[b0430] Taris T.W., Ybema J.F., Beckers D.G., Verheijden M.W., Geurts S.A., Kompier M.A. (2011). Investigating the associations among overtime work, health behaviors, and health: a longitudinal study among full-time employees. Int. J. Behav. Med..

[b0435] Teixeira, L.R., Azevedo, T.M., Bortkiewicz, A., Correa da Silva, D.T., de Abreu, W., de Almeida, M.S., de Araujo, M.A.N., Gadzicka, E., Ivanov, I.D., Leppink, N., Macedo, M.R.V., de, S.M.E.M.G., Pawlaczyk-Luszczynska, M., Pega, F., Pruss-Ustun, A.M., Siedlecka, J., Stevens, G.A., Ujita, Y., Braga, J.U., 2019. WHO/ILO work-related burden of disease and injury: Protocol for systematic reviews of exposure to occupational noise and of the effect of exposure to occupational noise on cardiovascular disease. Environ. Int. 125, 567–578. 10.1016/j.envint.2018.09.040 30683322

[b0440] Tenkate T., Adam B., Al-Rifai R.H., Chou B.R., Gobba F., Ivanov I.D., Leppink N., Loney T., Pega F., Peters C.E., Pruss-Ustun A.M., Silva Paulo M., Ujita Y., Wittlich M., Modenese A. (2019). WHO/ILO work-related burden of disease and injury: protocol for systematic reviews of occupational exposure to solar ultraviolet radiation and of the effect of occupational exposure to solar ultraviolet radiation on cataract. Environ. Int..

[b0445] Theorell T., Jood K., Jarvholm L.S., Vingard E., Perk J., Ostergren P.O., Hall C. (2016). A systematic review of studies in the contributions of the work environment to ischaemic heart disease development. Eur. J. Public Health.

[b0450] Theorell T., Rahe R.H. (1972). Behavior and life satisfactions characteristics of Swedish subjects with myocardial infarction. J. Chronic Dis..

[b0455] Thiel H.G., Parker D., Bruce T.A. (1973). Stress factors and the risk of myocardial infarction. J. Psychosom. Res..

[b0460] Toker S., Melamed S., Berliner S., Zeltser D., Shapira I. (2012). Burnout and risk of coronary heart disease: a prospective study of 8838 employees. Psychosom. Med..

[b0465] Veritas Health Innovation. Covidence systematic review software, Veritas Health Innovation, Melbourne, Australia. Available at www.covidence.org.

[b0470] Vesterinen H., Johnson P., Atchley D., Sutton P., Lam J., Zlatnik M., Sen S., Woodruff T. (2014). The relationship between fetal growth and maternal glomerular filtration rate: a systematic review. J. Maternal Fetal Neonatal. Med..

[b0475] Virtanen M., Ferrie J.E., Gimeno D., Vahtera J., Elovainio M., Singh-Manoux A., Marmot M.G., Kivimaki M. (2009). Long working hours and sleep disturbances: the Whitehall II prospective cohort study. Sleep.

[b0480] Virtanen M., Ferrie J.E., Singh-Manoux A., Shipley M.J., Vahtera J., Marmot M.G., Kivimaki M. (2010). Overtime work and incident coronary heart disease: the Whitehall II prospective cohort study. Eur. Heart J..

[b0485] Virtanen M., Heikkila K., Jokela M., Ferrie J.E., Batty G.D., Vahtera J., Kivimaki M. (2012). Long working hours and coronary heart disease: a systematic review and meta-analysis. Am. J. Epidemiol..

[b0490] Virtanen M., Jokela M., Nyberg S.T., Madsen I.E., Lallukka T., Ahola K., Alfredsson L., Batty G.D., Bjorner J.B., Borritz M., Burr H., Casini A., Clays E., De Bacquer D., Dragano N., Erbel R., Ferrie J.E., Fransson E.I., Hamer M., Heikkila K., Jockel K.H., Kittel F., Knutsson A., Koskenvuo M., Ladwig K.H., Lunau T., Nielsen M.L., Nordin M., Oksanen T., Pejtersen J.H., Pentti J., Rugulies R., Salo P., Schupp J., Siegrist J., Singh-Manoux A., Steptoe A., Suominen S.B., Theorell T., Vahtera J., Wagner G.G., Westerholm P.J., Westerlund H., Kivimaki M. (2015). Long working hours and alcohol use: systematic review and meta-analysis of published studies and unpublished individual participant data. BMJ.

[b0495] Virtanen M., Kivimaki M. (2018). Long working hours and risk of cardiovascular disease. Curr. Cardiol. Rep..

[b0500] Viswanathan, M., Ansari, M.T., Berkman, N.D., Chang, S., Hartling, L., McPheeters, M., Santaguida, P.L., Shamliyan, T., Singh, K., Tsertsvadze, A., Treadwell, J.R., 2008. Assessing the Risk of Bias of Individual Studies in Systematic Reviews of Health Care Interventions. Methods Guide for Effectiveness and Comparative Effectiveness Reviews. Rockville (MD). 22479713

[b0505] Wong K., Chan A.H.S., Ngan S.C. The (2019). Effect of long working hours and overtime on occupational health: a meta-analysis of evidence from 1998 to 2018. Int. J. Environ. Res. Public Health.

[b0510] Woodruff T.J., Sutton P. (2014). The Navigation Guide systematic review methodology: a rigorous and transparent method for translating environmental health science into better health outcomes. Environ. Health Perspect..

[b0515] World Health Organization (2015). ICD-10: International Statistical Classification of Diseases and Related Health Problems.

[b0520] World Health Organization (2017). Department of Information, Evidence and Research, ed. WHO Methods and Data Sources for Global Burden of Disease Estimates 2000–2015 Global Health Estimates Technical Paper WHO/HIS/IER/GHE/20171.

[b0525] World Health Organization (2020). Disease Burden and Mortality Estimates: Disease Burden, 2000–2016.

