# Supplementary material for: The effect of exposure to long working hours on ischaemic heart disease: A systematic review and meta-analysis from the WHO/ILO Joint Estimates of the Work-related Burden of Disease and Injury
Source: Environ Int. 2020 Sep;142:105739. doi: 10.1016/j.envint.2020.105739 (PMC7339147; doi:10.1016/j.envint.2020.105739)
Supplement: Supplementary data 1 [file mmc1.docx]

Appendix 1 Description of missing data requested and received

| Study ID | Description of requested missing data | Person(s) from whom missing data were requested | Date of request(s)  (YYYY-MM-DD) | Data received |
| --- | --- | --- | --- | --- |
| Cheng 2014 | LWH re-categorization: 35-40, 41-48, 49-54, ≥55 hours/week.  Stratification by age, sex, and SES, if possible. | Dr. Yawen Cheng | 2019-02-22  2019-03-11  2019-03-27 | The principal study author reported that the primary data cannot be re-categorized into our standard exposure categories. |
| Fukuoka 2005 | LWH re-categorization: 35-40, 41-48, 49-54, ≥55 hours/week.  Stratification by age, sex, and SES, if possible. | Dr. Yoshimi Fukuoka | 2019-02-22  2019-03-11  2019-04-17 | The principal study author shared the requested missing data that they could offer. |
| Hannerz 2018 | LWH re-categorization: 35-40, 41-48, 49-54, ≥55 hours/week.  Stratification by age, sex, and SES, if possible. | Dr. Harald Hannerz | 2019-02-22  2019-07-09 | The principal study author shared the requested missing data that they could offer. |
| Jeong 2013 | LWH re-categorization: 35-40, 41-48, 49-54, ≥55 hours/week.  Stratification by age, sex, and SES, if possible. | Dr. Inchul Jeong | 2018-09-22  2018-11-05 | The principal study author shared the requested missing data that they could offer. |
| Liu 2002 | LWH re-categorization: 35-40, 41-48, 49-54, ≥55 hours/week.  Stratification by age, sex, and SES, if possible. | Dr. Y. Liu | 2019-02-22  2019-03-11  2019-04-17 | The principal study author did not respond. |
| Ma 2017 | LWH re-categorization: 35-40, 41-48, 49-54, ≥55 hours/week.  Stratification by age, sex, and SES, if possible. | Dr. Lian-Sheng Wang | 2019-02-22  2019-03-11  2019-04-17 | The principal study author did not respond. |
| Marchand 2017 | LWH re-categorization: 35-40, 41-48, 49-54, ≥55 hours/week.  Stratification by age, sex, and SES, if possible. | Dr. Alain Marchand | 2019-02-22  2019-03-11 | The principal study author reported that the primary data cannot be re-categorized into our standard exposure categories. |
| Sokejima 1998 | LWH re-categorization: 35-40, 41-48, 49-54, ≥55 hours/week.  Stratification by age, sex, and SES, if possible. | Dr. Shigeru Sokejima | 2019-02-22  2019-03-11  2019-04-17 | The principal study author did not respond. |
| Theorell 1972 | LWH re-categorization: 35-40, 41-48, 49-54, ≥55 hours/week.  Stratification by age, sex, and SES, if possible. | Dr. Töres Theorell | 2019-02-22 | The principal study author reported that the primary data cannot be found because this study was conducted more than three decades ago. |
| Kivimaki 2015 | LWH re-categorization: 35-40, 41-48, 49-54, ≥55 hours/week.  Stratification by age, sex, and SES, if possible. | Dr. Mika Kivimaki | 2019-03-30  2019-06-18  2019-06-30 | The principal study author shared the requested missing data that they could offer. |
| Hayashi 2019 | LWH re-categorization: 35-40, 41-48, 49-54, ≥55 hours/week.  Stratification by age, sex, and SES, if possible. | Dr. Hiroyasu Iso | 2019-06-03  2019-06-11  2019-07-02 | The principal study author did not respond. |

LWH: long working hours

*Appendix 2 Selected excluded studies and reason for their exclusion*

| Study (Study ID) | Reason for exclusion |
| --- | --- |
| Albert 2017 | Ineligible outcome |
| Alfredsson 1993 | Ineligible exposure |
| Alfredsson 1982 | Ineligible exposure |
| Alfredsson 1983 | Ineligible exposure |
| Allesoe 2015 | Ineligible exposure |
| Allesøe 2010 | Ineligible exposure |
| Allesoe 2016 | Ineligible patient population |
| Alterman 1994 | Ineligible exposure |
| Andre-Petersson 2007 | Ineligible exposure |
| Anonymous author 1998 | Ineligible study type |
| Apostolopoulos 2010 | Ineligible study type |
| Asgeirsdottir 2014 | Ineligible exposure |
| Bannai 2014 | Ineligible study type |
| Berstad 2017 | Ineligible exposure |
| Bigert 2003 | Ineligible patient population |
| Bigert 2013 | Ineligible patient population |
| Bildt 2006 | Ineligible exposure |
| Boedeker 2001 | Ineligible outcome |
| Boggild 2001 | Ineligible outcome |
| Brisbois 2010 | Ineligible study type |
| Buell 1960 | Ineligible study type |
| Chen 2018 | Ineligible outcome |
| Chevrolle 1963 | Ineligible study type |
| Ciswicka-Sznajderman 1973 | Ineligible exposure |
| Conway 2017 | Ineligible outcome |
| Conway 2016 | Ineligible outcome |
| Dembe 2005 | Ineligible outcome |
| Dembe 2016 | Ineligible study type |
| Gafarov 2015 | Ineligible exposure |
| Kang 2012 | Ineligible study type |
| Kivimaki 2011 | Duplicate study |
| Kivimaki 2017 | Ineligible outcome |
| Landsbergis 2013 | Ineligible study type |
| Lee 2018 | Ineligible study type |
| Lee 2019 | Ineligible outcome |
| Lin 2018 | Ineligible study type |
| Marchand 2017 | Ineligible exposure |
| Mortensen 2017 | Ineligible outcome |
| Mortensen 2010 | Duplicate study |
| Nowrouzi-Kia 2018 | Ineligible study type |
| Shin 2017 | Ineligible study type |
| Szerencsi 2014 | Ineligible exposure |
| Szerencsi 2013 | Ineligible exposure |
| Tarumi 2003 | Ineligible outcome |
| Tuchsen 2005 | Ineligible patient population |
| Uchiyama 2005 | Ineligible outcome |
| van der Hulst 2003 | Ineligible study type |
| Virtanen 2012 | Ineligible study type |

Appendix 3 Search strategies for electronic academic databases

**WHO International Clinical Trials Register Platform (Standard search)**

Date searched: 06.07.2018

Records retrieved: 238

Strategy:

work* AND hours AND myocardial OR

work* AND hours AND heart OR

work* AND hours AND coronary OR

work* AND hours AND cardiovascular OR

work* AND hours AND angina OR

work* AND burnout AND myocardial OR

work* AND burnout AND heart OR

work* AND burnout AND coronary OR

work* AND burnout AND cardiovascular OR

work* AND burnout AND angina OR

work* AND stress AND myocardial OR

work* AND stress AND heart OR

work* AND stress AND coronary OR

work* AND stress AND cardiovascular OR

work* AND stress AND angina OR

employee* AND myocardial OR

employee* AND heart OR

employee* AND coronary OR

employee* AND cardiovascular OR

employee* AND angina OR

overwork* OR

overtime*

**MEDLINE (Ovid)**

Date searched: 06.07.2018; Date of top-up search: 27.08.2019

Records retrieved: 1118 (main search); 43 (top-up search)

Strategy:

1. Workload/
2. Burnout, Professional/
3. Work Schedule Tolerance/
4. ((work or working or worked) adj3 hours).tw.
5. ((work or working or worked) adj3 overtime).tw.
6. ((working or work) adj1 long).tw.
7. ((job or work or occcupational) adj (strain or stress)).tw.
8. (overwork*).tw.
9. (work* adj3 overload*).tw.
10. ((work or occupational or professional) adj3 burnout).tw.
11. or/1-10
12. exp Myocardial Ischemia/
13. *Cardiovascular Diseases/ep, et, mo (epidemiology, etiology, mortality)
14. angina.tw.
15. coronary heart disease*.tw.
16. cardiovascular disease*.tw.
17. isch?emic heart disease*.tw.
18. (myocardial adj (infarct* or ischemia)).tw.
19. coronary event*.tw.
20. cardiovascular event*.tw.
21. or/12-20
22. 11 and 21
23. (comment or letter or editorial or case reports).pt.
24. 22 not 23
25. exp animals/ not humans/
26. 24 not 25

**Scopus**

Date searched: 06.07.2018

Records retrieved: 1412

Strategy:

TITLE-ABS-KEY(((work W/3 hours) OR (working W/3 hours) OR (worked W/3 hours) OR (work W/3 overtime) OR (working W/3 overtime) OR (worked W/3 overtime) OR (working W/1 long) OR (work W/1 long) OR "job strain" OR "job stress" OR "work strain" OR "work stress" OR "occcupational strain" OR "occupational stress" OR overwork* OR (work* W/3 overload*) OR (work W/3 burnout) OR (occupational W/3 burnout) OR (professional W/3 burnout)) AND (angina OR "coronary heart disease*" OR "cardiovascular disease*" OR "ischemic heart disease*" OR "ischaemic heart disease*" OR "myocardial infarct*" OR "myocardial ischemia" OR "coronary event*" OR "cardiovascular event*"))

AND NOT ( LIMIT-TO ( DOCTYPE , "ar" ) OR LIMIT-TO ( DOCTYPE , "re" ) OR LIMIT-TO ( DOCTYPE , "cp" ) OR LIMIT-TO ( DOCTYPE , "no" ) OR LIMIT-TO ( DOCTYPE , "ch" ) OR LIMIT-TO ( DOCTYPE , "sh" ) OR LIMIT-TO ( DOCTYPE , "ip" ) OR LIMIT-TO ( DOCTYPE , "bk" ) ) = Article, Review, Conference Paper, Book Chapter, Short Survey, Article in Press, Book

**Web of Science**

Date searched: 06.07.2018

Records retrieved: 582

Databases used: Science Citation Index Expanded (1945-present), Social Sciences Citation Index (1956-present), Arts & Humanities Citation Index (1975-present), Emerging Sources Citation Index (2015-present)

Strategy:

TS=(((work NEAR/3 hours) OR (working NEAR/3 hours) OR (worked NEAR/3 hours) OR (work NEAR/3 overtime) OR (working NEAR/3 overtime) OR (worked NEAR/3 overtime) OR (working NEAR/1 long) OR (work NEAR/1 long) OR overwork* OR (work* NEAR/3 overload*) OR (work NEAR/3 burnout) OR (occupational NEAR/3 burnout) OR (professional NEAR/3 burnout)) AND (angina OR "coronary heart disease*" OR "cardiovascular disease*" OR "isch$emic heart disease*" OR "myocardial infarct*" OR "myocardial ischemia" OR "coronary event*" OR "cardiovascular event*"))

AND NOT DOCUMENT TYPES: (Article OR Abstract of Published Item OR Correction OR Correction, Addition OR Data Paper OR Discussion OR Early Access OR Meeting Abstract OR Meeting Summary OR Note OR Proceedings Paper OR Reprint OR Retracted Publication OR Retraction OR Review)

**CISDOC (**<http://www.ilo.org/dyn/cisdoc2/cismain.home>**)**

Date searched: 06.07.2018

Records retrieved: 41

Strategy:

(overtime myocardial) OR (overtime "heart disease*") OR (overtime coronary)

(overtime cardiovascular) OR (overtime angina)

("long work*" myocardial) OR ("long work*" "heart disease*")

("long work*" coronary) OR ("long work*" cardiovascular)

("long work*" angina)

("long hours" myocardial) OR ("long hours" "heart disease*")

("long hours" coronary) OR ("long hours" cardiovascular)

("long hours" angina)

("work* hours" myocardial) OR ("work* hours" "heart disease*")

("work* hours" coronary) OR ("work* hours" cardiovascular)

("work* hours" angina)

(overload myocardial) OR (overload "heart disease*")

(overload coronary) OR (overload cardiovascular) OR (overload angina)

(overwork* myocardial) OR (overwork* "heart disease*")

(overwork* coronary) OR (overwork* cardiovascular) OR (overwork* angina)

(workload myocardial) OR (workload "heart disease*") OR (workload coronary)

(workload cardiovascular) OR (workload angina)

**PsycINFO (Ovid)**

Date searched: 06.07.2018

Records retrieved: 398

Strategy:

1. Occupational Stress/
2. Work Load/
3. Work-Life Balance/
4. Work Scheduling/
5. Work Week Length/
6. ((work or working or worked) adj3 hours).tw.
7. ((work or working or worked) adj3 overtime).tw.
8. ((working or work) adj1 long).tw.
9. ((job or work or occcupational) adj (strain or stress)).tw.
10. (overwork*).tw.
11. (work* adj3 overload*).tw.
12. ((work or occupational or professional) adj3 burnout).tw.
13. or/1-12
14. Cardiovascular Disorders/
15. exp Heart Disorders/
16. angina.tw.
17. coronary heart disease*.tw.
18. cardiovascular disease*.tw.
19. isch?emic heart disease*.tw.
20. (myocardial adj (infarct* or ischemia)).tw.
21. coronary event*.tw.
22. cardiovascular event*.tw.
23. or/14-22
24. 13 and 23
25. book.pt.
26. 24 not 25

*Appendix 4 Questions used in includes studies that assess the outcome with self-reported physician diagnosis*

|  | **Study ID** | **Question** |
| --- | --- | --- |
| *1* | Kivimaki 2015 - ACL 1986 | “Have you had a heart attack or other heart trouble during the last 12 months?” |
| *2* | Kivimaki 2015 - Alameda 1973 | “Here is a list of medical conditions that usually last for some time. Have you EVER had any of these conditions? Please answer Yes or No for each condition.” Relevant item: “Heart trouble” |
| *3* | Kivimaki 2015 - HILDA 2003 | “Have you ever been told by a doctor or nurse that you have any of the long-term health conditions listed below? Please only include those conditions that have lasted or are likely to last for six months or more. Relevant item: “Heart / Coronary disease” |
| *4* | Kivimaki 2015 - MIDUS 1995 | “Have you ever had heart trouble suspected or confirmed by a doctor?” |
| *5* | Kivimaki 2015 - NHANES I 1982 | “Did a doctor ever tell you that you had any of the following conditions?” Relevant item: Heart attack |
| *6* | Kivimaki 2015 - WLSG 1992 | “Has a medical professional ever said you have heart trouble?” |
| *7* | Kivimaki 2015 - WLSS 1993 | “Has a medical professional ever said you have heart trouble?” |

Source: Personal communication, Markus Jokela, 31 Jan 2017.

*Appendix 5 Justifications for risk of bias ratings*

The justifications risk of bias ratings for each domain by study are identical for both outcomes with any studies with eligible evidence (i.e., IHD incidence and mortality).

*Table A5.1 Risk of bias, [Kivimaki 2015 - Virtanen 2010]*

| **Domain** | **Rating** | **Justification for rating** |
| --- | --- | --- |
| Bias in selection of participants into the study | Probably high | Prospective cohort study, prevalent cases at baseline were excluded.  Low participation rate (73%) would potentially introduce bias. |
| Bias due to a lack of blinding of study personnel | Probably low | Not clear if investigators were blinded but it is not assumed that this is a major bias in this observational study |
| Bias due to exposure misclassification | Probably low | Working hours were self-reported, which seem to be as precise as administrative data |
| Bias in the outcome misclassification | Low | Register-based outcome |
| Confounding | Low | Analyses were adjusted to most important confounders (age, sex, and socioeconomic status) and other covariates |
| Bias due to incomplete outcome data | Low | Register-based outcome |
| Bias due to selective reporting of outcomes | Low | Not identified |
| Bias due to conflict of interest | Low | Not identified |
| Other bias | Low | Not identified |

*Table A5.2 Risk of bias, [Kivimaki 2015 - Netterstrom 2010]*

| **Domain** | **Rating** | **Justification for rating** |
| --- | --- | --- |
| Bias in selection of participants into the study | Probably low | Prospective cohort study, prevalent cases at baseline were excluded |
| Bias due to a lack of blinding of study personnel | Probably low | Not clear if investigators were blinded but it is not assumed that this is a major bias in this observational study |
| Bias due to exposure misclassification | Probably low | Working hours were self-reported, which seem to be as precise as administrative data |
| Bias in the outcome misclassification | Low | Register-based outcome |
| Confounding | Probably low | Analyses were adjusted to most important confounders (age, sex, and socioeconomic status) |
| Bias due to incomplete outcome data | Low | Register-based outcome |
| Bias due to selective reporting of outcomes | Low | Not identified |
| Bias due to conflict of interest | Low | Not identified |
| Other bias | Low | Not identified |

*Table A5.3 Risk of bias, [Kivimaki 2015 - Toker 2012]*

| **Domain** | **Rating** | **Justification for rating** |
| --- | --- | --- |
| Bias in selection of participants into the study | Probably low | Prospective cohort study, prevalent cases at baseline were excluded |
| Bias due to a lack of blinding of study personnel | Probably low | Not clear if investigators were blinded but it is not assumed that this is a major bias in this observational study |
| Bias due to exposure misclassification | Probably low | Working hours were self-reported, which seem to be as precise as administrative data |
| Bias in the outcome misclassification | Low | Clinical assessment of IHD |
| Confounding | Probably high | Analyses were adjusted to most important confounders (age, sex, and socioeconomic status) and other covariates, adjusting for burnout and long working hours is probably overadjustment. |
| Bias due to incomplete outcome data | Low | Almost all participants followed up and those few who did not show for the follow-up assessment provided self-reported assessment of IHD incidence |
| Bias due to selective reporting of outcomes | Low | Not identified |
| Bias due to conflict of interest | Low | Not identified |
| Other bias | Low | Not identified |

*Table A5.4 Risk of bias, [Kivimaki 2015 - WOLF-S 1992]*

| **Domain** | **Rating** | **Justification for rating** |
| --- | --- | --- |
| Bias in selection of participants into the study | Probably low | Prospective cohort study, prevalent cases at baseline were excluded |
| Bias due to a lack of blinding of study personnel | Probably low | Not clear if investigators were blinded but it is not assumed that this is a major bias in this observational study |
| Bias due to exposure misclassification | Probably low | Working hours were self-reported, which seem to be as precise as administrative data |
| Bias in the outcome misclassification | Low | Clinical diagnosis |
| Confounding | Probably low | Analyses were adjusted to most important confounders (age, sex, and socioeconomic status) |
| Bias due to incomplete outcome data | Low | Not identified |
| Bias due to selective reporting of outcomes | Low | Not identified |
| Bias due to conflict of interest | Low | Not identified |
| Other bias | Low | Not identified |

*Table A5.5 Risk of bias, [Kivimaki 2015 - Belstress 1994]*

| **Domain** | **Rating** | **Justification for rating** |
| --- | --- | --- |
| Bias in selection of participants into the study | Probably low | Prospective cohort study, prevalent cases at baseline were excluded |
| Bias due to a lack of blinding of study personnel | Probably low | Not clear if investigators were blinded but it is not assumed that this is a major bias in this observational study |
| Bias due to exposure misclassification | Probably low | Working hours were self-reported, which seem to be as precise as administrative data |
| Bias in the outcome misclassification | Low | Clinical diagnosis |
| Confounding | Probably low | Analyses were adjusted to most important confounders (age, sex, and socioeconomic status) |
| Bias due to incomplete outcome data | Low | Not identified |
| Bias due to selective reporting of outcomes | Low | Not identified |
| Bias due to conflict of interest | Low | Not identified |
| Other bias | Low | Not identified |

*Table A5.6 Risk of bias, [Kivimaki 2015 - WOLF-N 1996]*

| **Domain** | **Rating** | **Justification for rating** |
| --- | --- | --- |
| Bias in selection of participants into the study | Probably low | Prospective cohort study, prevalent cases at baseline were excluded |
| Bias due to a lack of blinding of study personnel | Probably low | Not clear if investigators were blinded but it is not assumed that this is a major bias in this observational study |
| Bias due to exposure misclassification | Probably low | Working hours were self-reported, which seem to be as precise as administrative data |
| Bias in the outcome misclassification | Low | Clinical diagnosis |
| Confounding | Probably low | Analyses were adjusted to most important confounders (age, sex, and socioeconomic status) |
| Bias due to incomplete outcome data | Low | Not identified |
| Bias due to selective reporting of outcomes | Low | Not identified |
| Bias due to conflict of interest | Low | Not identified |
| Other bias | Low | Not identified |

*Table A5.7 Risk of bias, [Kivimaki 2015 - COPSOQ-I 1997]*

| **Domain** | **Rating** | **Justification for rating** |
| --- | --- | --- |
| Bias in selection of participants into the study | Probably high | Prospective cohort study, prevalent cases at baseline were excluded.  Low participation rate (62%) would potentially introduce bias. |
| Bias due to a lack of blinding of study personnel | Probably low | Not clear if investigators were blinded but it is not assumed that this is a major bias in this observational study |
| Bias due to exposure misclassification | Probably low | Working hours were self-reported, which seem to be as precise as administrative data |
| Bias in the outcome misclassification | Low | Clinical diagnosis |
| Confounding | Probably low | Analyses were adjusted to most important confounders (age, sex, and socioeconomic status) |
| Bias due to incomplete outcome data | Low | Not identified |
| Bias due to selective reporting of outcomes | Low | Not identified |
| Bias due to conflict of interest | Low | Not identified |
| Other bias | Low | Not identified |

*Table A5.8 Risk of bias, [Kivimaki 2015 - HeSSup 1998]*

| **Domain** | **Rating** | **Justification for rating** |
| --- | --- | --- |
| Bias in selection of participants into the study | Probably high | Prospective cohort study, prevalent cases at baseline were excluded.  Low participation rate (40%) would potentially introduce bias. |
| Bias due to a lack of blinding of study personnel | Probably low | Not clear if investigators were blinded but it is not assumed that this is a major bias in this observational study |
| Bias due to exposure misclassification | Probably low | Working hours were self-reported, which seem to be as precise as administrative data |
| Bias in the outcome misclassification | Low | Clinical diagnosis |
| Confounding | Probably low | Analyses were adjusted to most important confounders (age, sex, and socioeconomic status) |
| Bias due to incomplete outcome data | Low | Not identified |
| Bias due to selective reporting of outcomes | Low | Not identified |
| Bias due to conflict of interest | Low | Not identified |
| Other bias | Low | Not identified |

*Table A5.9 Risk of bias, [Kivimaki 2015 - FPS 2000]*

| **Domain** | **Rating** | **Justification for rating** |
| --- | --- | --- |
| Bias in selection of participants into the study | Probably high | Prospective cohort study, prevalent cases at baseline were excluded.  Low participation rate (67-70%) would potentially introduce bias. |
| Bias due to a lack of blinding of study personnel | Probably low | Not clear if investigators were blinded but it is not assumed that this is a major bias in this observational study |
| Bias due to exposure misclassification | Probably low | Working hours were self-reported, which seem to be as precise as administrative data |
| Bias in the outcome misclassification | Low | Clinical diagnosis |
| Confounding | Probably low | Analyses were adjusted to most important confounders (age, sex, and socioeconomic status) |
| Bias due to incomplete outcome data | Low | Not identified |
| Bias due to selective reporting of outcomes | Low | Not identified |
| Bias due to conflict of interest | Low | Not identified |
| Other bias | Low | Not identified |

*Table A5.10 Risk of bias, [Kivimaki 2015 - HNR 2000]*

| **Domain** | **Rating** | **Justification for rating** |
| --- | --- | --- |
| Bias in selection of participants into the study | Probably low | Prospective cohort study, prevalent cases at baseline were excluded |
| Bias due to a lack of blinding of study personnel | Probably low | Not clear if investigators were blinded but it is not assumed that this is a major bias in this observational study |
| Bias due to exposure misclassification | Probably low | Working hours were self-reported, which seem to be as precise as administrative data |
| Bias in the outcome misclassification | Low | Clinical diagnosis |
| Confounding | Probably low | Analyses were adjusted to most important confounders (age, sex, and socioeconomic status) |
| Bias due to incomplete outcome data | Low | Not identified |
| Bias due to selective reporting of outcomes | Low | Not identified |
| Bias due to conflict of interest | Low | Not identified |
| Other bias | Low | Not identified |

*Table A5.11 Risk of bias, [Kivimaki 2015 - DWECS 2000]*

| **Domain** | **Rating** | **Justification for rating** |
| --- | --- | --- |
| Bias in selection of participants into the study | Probably low | Prospective cohort study, prevalent cases at baseline were excluded |
| Bias due to a lack of blinding of study personnel | Probably low | Not clear if investigators were blinded but it is not assumed that this is a major bias in this observational study |
| Bias due to exposure misclassification | Probably low | Working hours were self-reported, which seem to be as precise as administrative data |
| Bias in the outcome misclassification | Low | Clinical diagnosis |
| Confounding | Probably low | Analyses were adjusted to most important confounders (age, sex, and socioeconomic status) |
| Bias due to incomplete outcome data | Low | Not identified |
| Bias due to selective reporting of outcomes | Low | Not identified |
| Bias due to conflict of interest | Low | Not identified |
| Other bias | Low | Not identified |

*Table A5.12 Risk of bias, [Kivimaki 2015 - COPSOQ-II 2004]*

| **Domain** | **Rating** | **Justification for rating** |
| --- | --- | --- |
| Bias in selection of participants into the study | Probably high | Prospective cohort study, prevalent cases at baseline were excluded.  Low participation rate (60%) would potentially introduce bias. |
| Bias due to a lack of blinding of study personnel | Probably low | Not clear if investigators were blinded but it is not assumed that this is a major bias in this observational study |
| Bias due to exposure misclassification | Probably low | Working hours were self-reported, which seem to be as precise as administrative data |
| Bias in the outcome misclassification | Low | Clinical diagnosis |
| Confounding | Probably low | Analyses were adjusted to most important confounders (age, sex, and socioeconomic status) |
| Bias due to incomplete outcome data | Low | Not identified |
| Bias due to selective reporting of outcomes | Low | Not identified |
| Bias due to conflict of interest | Low | Not identified |
| Other bias | Low | Not identified |

*Table A5.13 Risk of bias, [Kivimaki 2015 - IPAW 1996]*

| **Domain** | **Rating** | **Justification for rating** |
| --- | --- | --- |
| Bias in selection of participants into the study | Probably high | Prospective cohort study, prevalent cases at baseline were excluded.  Low participation rate (76%) would potentially introduce bias. |
| Bias due to a lack of blinding of study personnel | Probably low | Not clear if investigators were blinded but it is not assumed that this is a major bias in this observational study |
| Bias due to exposure misclassification | Probably low | Working hours were self-reported, which seem to be as precise as administrative data |
| Bias in the outcome misclassification | Low | Clinical diagnosis |
| Confounding | Probably low | Analyses were adjusted to most important confounders (age, sex, and socioeconomic status) |
| Bias due to incomplete outcome data | Low | Not identified |
| Bias due to selective reporting of outcomes | Low | Not identified |
| Bias due to conflict of interest | Low | Not identified |
| Other bias | Low | Not identified |

*Table A5.14 Risk of bias, [Kivimaki 2015 - PUMA 1999]*

| **Domain** | **Rating** | **Justification for rating** |
| --- | --- | --- |
| Bias in selection of participants into the study | Probably low | Prospective cohort study, prevalent cases at baseline were excluded |
| Bias due to a lack of blinding of study personnel | Probably low | Not clear if investigators were blinded but it is not assumed that this is a major bias in this observational study |
| Bias due to exposure misclassification | Probably low | Working hours were self-reported, which seem to be as precise as administrative data |
| Bias in the outcome misclassification | Low | Clinical diagnosis |
| Confounding | Probably low | Analyses were adjusted to most important confounders (age, sex, and socioeconomic status) |
| Bias due to incomplete outcome data | Low | Not identified |
| Bias due to selective reporting of outcomes | Low | Not identified |
| Bias due to conflict of interest | Low | Not identified |
| Other bias | Low | Not identified |

*Table A5.15 Risk of bias, [Kivimaki 2015 - NWCS 2005]*

| **Domain** | **Rating** | **Justification for rating** |
| --- | --- | --- |
| Bias in selection of participants into the study | Probably low | Prospective cohort study, prevalent cases at baseline were excluded |
| Bias due to a lack of blinding of study personnel | Probably low | Not clear if investigators were blinded but it is not assumed that this is a major bias in this observational study |
| Bias due to exposure misclassification | Probably low | Working hours were self-reported, which seem to be as precise as administrative data |
| Bias in the outcome misclassification | Low | Clinical diagnosis |
| Confounding | Probably low | Analyses were adjusted to most important confounders (age, sex, and socioeconomic status) |
| Bias due to incomplete outcome data | Low | Not identified |
| Bias due to selective reporting of outcomes | Low | Not identified |
| Bias due to conflict of interest | Low | Not identified |
| Other bias | Low | Not identified |

*Table A5.16 Risk of bias, [Kivimaki 2015 - Alameda 1973]*

| **Domain** | **Rating** | **Justification for rating** |
| --- | --- | --- |
| Bias in selection of participants into the study | Probably low | Prospective cohort study, prevalent cases at baseline were excluded |
| Bias due to a lack of blinding of study personnel | Probably low | Not clear if investigators were blinded but it is not assumed that this is a major bias in this observational study |
| Bias due to exposure misclassification | Probably low | Working hours were self-reported, which seem to be as precise as administrative data |
| Bias in the outcome misclassification | High | Self-reported heart trouble |
| Confounding | Probably low | Analyses were adjusted to most important confounders (age, sex, and socioeconomic status) |
| Bias due to incomplete outcome data | Low | Not identified |
| Bias due to selective reporting of outcomes | Low | Not identified |
| Bias due to conflict of interest | Low | Not identified |
| Other bias | Low | Not identified |

*Table A5.17 Risk of bias, [Kivimaki 2015 - NHANES I 1982]*

| **Domain** | **Rating** | **Justification for rating** |
| --- | --- | --- |
| Bias in selection of participants into the study | Probably low | Prospective cohort study, prevalent cases at baseline were excluded |
| Bias due to a lack of blinding of study personnel | Probably low | Not clear if investigators were blinded but it is not assumed that this is a major bias in this observational study |
| Bias due to exposure misclassification | Probably low | Working hours were self-reported, which seem to be as precise as administrative data |
| Bias in the outcome misclassification | Probably low | Self-reported heart attack with physician diagnosis |
| Confounding | Probably low | Analyses were adjusted to most important confounders (age, sex, and socioeconomic status) |
| Bias due to incomplete outcome data | Low | Not identified |
| Bias due to selective reporting of outcomes | Low | Not identified |
| Bias due to conflict of interest | Low | Not identified |
| Other bias | Low | Not identified |

*Table A5.18 Risk of bias, [Kivimaki 2015 - ACL 1986]*

| **Domain** | **Rating** | **Justification for rating** |
| --- | --- | --- |
| Bias in selection of participants into the study | Probably high | Prospective cohort study, prevalent cases at baseline were excluded.  Probabilistic sampling methods were employed and 30% of sampled households and 32% of sampled individuals were not interviewed. Furthermore, an oversampling of blacks and persons aged 60 years and older than might represent a bias. |
| Bias due to a lack of blinding of study personnel | Probably low | Not clear if investigators were blinded but it is not assumed that this is a major bias in this observational study |
| Bias due to exposure misclassification | Probably low | Working hours were self-reported, which seem to be as precise as administrative data |
| Bias in the outcome misclassification | Probably high | Self-reported heart attack or other heart trouble |
| Confounding | Probably low | Analyses were adjusted to most important confounders (age, sex, and socioeconomic status) |
| Bias due to incomplete outcome data | Low | Not identified |
| Bias due to selective reporting of outcomes | Low | Not identified |
| Bias due to conflict of interest | Low | Not identified |
| Other bias | Low | Not identified |

*Table A5.19 Risk of bias, [Kivimaki 2015 - WLSG 1992]*

| **Domain** | **Rating** | **Justification for rating** |
| --- | --- | --- |
| Bias in selection of participants into the study | Probably low | Prospective cohort study, prevalent cases at baseline were excluded |
| Bias due to a lack of blinding of study personnel | Probably low | Not clear if investigators were blinded but it is not assumed that this is a major bias in this observational study |
| Bias due to exposure misclassification | Probably low | Working hours were self-reported, which seem to be as precise as administrative data |
| Bias in the outcome misclassification | Probably high | Self-reported heart trouble with physician diagnosis |
| Confounding | Probably low | Analyses were adjusted to most important confounders (age, sex, and socioeconomic status) |
| Bias due to incomplete outcome data | Low | Not identified |
| Bias due to selective reporting of outcomes | Low | Not identified |
| Bias due to conflict of interest | Low | Not identified |
| Other bias | Low | Not identified |

*Table A5.20 Risk of bias, [Kivimaki 2015 - WLSS 1993]*

| **Domain** | **Rating** | **Justification for rating** |
| --- | --- | --- |
| Bias in selection of participants into the study | Probably low | Prospective cohort study, prevalent cases at baseline were excluded |
| Bias due to a lack of blinding of study personnel | Probably low | Not clear if investigators were blinded but it is not assumed that this is a major bias in this observational study |
| Bias due to exposure misclassification | Probably low | Working hours were self-reported, which seem to be as precise as administrative data |
| Bias in the outcome misclassification | Probably high | Self-reported heart trouble with physician diagnosis |
| Confounding | Probably low | Analyses were adjusted to most important confounders (age, sex, and socioeconomic status) |
| Bias due to incomplete outcome data | Low | Not identified |
| Bias due to selective reporting of outcomes | Low | Not identified |
| Bias due to conflict of interest | Low | Not identified |
| Other bias | Low | Not identified |

*Table A5.21 Risk of bias, [Kivimaki 2015 - MIDUS 1995]*

| **Domain** | **Rating** | **Justification for rating** |
| --- | --- | --- |
| Bias in selection of participants into the study | Probably low | Prospective cohort study, prevalent cases at baseline were excluded |
| Bias due to a lack of blinding of study personnel | Probably low | Not clear if investigators were blinded but it is not assumed that this is a major bias in this observational study |
| Bias due to exposure misclassification | Probably low | Working hours were self-reported, which seem to be as precise as administrative data |
| Bias in the outcome misclassification | High | Self-reported heart trouble with physician suspection or diagnosis |
| Confounding | Probably low | Analyses were adjusted to most important confounders (age, sex, and socioeconomic status) |
| Bias due to incomplete outcome data | Low | Not identified |
| Bias due to selective reporting of outcomes | Low | Not identified |
| Bias due to conflict of interest | Low | Not identified |
| Other bias | Low | Not identified |

*Table A5.22 Risk of bias, [Kivimaki 2015 - HILDA 2003]*

| **Domain** | **Rating** | **Justification for rating** |
| --- | --- | --- |
| Bias in selection of participants into the study | Probably low | Prospective cohort study, prevalent cases at baseline were excluded |
| Bias due to a lack of blinding of study personnel | Probably low | Not clear if investigators were blinded but it is not assumed that this is a major bias in this observational study |
| Bias due to exposure misclassification | Probably low | Working hours were self-reported, which seem to be as precise as administrative data |
| Bias in the outcome misclassification | Probably high | Self-reported heart disease with physician or nurse diagnosis |
| Confounding | Probably low | Analyses were adjusted to most important confounders (age, sex, and socioeconomic status) |
| Bias due to incomplete outcome data | Low | Not identified |
| Bias due to selective reporting of outcomes | Low | Not identified |
| Bias due to conflict of interest | Low | Not identified |
| Other bias | Low | Not identified |

*Table A5.23 Risk of bias, [Hannerz 2018]*

| **Domain** | **Rating** | **Justification for rating** |
| --- | --- | --- |
| Bias in selection of participants into the study | Probably low | Prospective cohort study, prevalent cases at baseline were excluded |
| Bias due to a lack of blinding of study personnel | Probably low | Not clear if investigators were blinded but it is not assumed that this is a major bias in this observational study |
| Bias due to exposure misclassification | Probably low | Working hours were self-reported, which seem to be as precise as administrative data |
| Bias in the outcome misclassification | Low | Register-based outcome assessment |
| Confounding | Probably low | Analyses were adjusted to most important confounders (age, sex, and socioeconomic status) |
| Bias due to incomplete outcome data | Low | Register-based outcome assessment |
| Bias due to selective reporting of outcomes | Low | Not identified |
| Bias due to conflict of interest | Low | Not identified |
| Other bias | Low | Not identified |

*Table A5.24 Risk of bias, [Hayashi 2019]*

| **Domain** | **Rating** | **Justification for rating** |
| --- | --- | --- |
| Bias in selection of participants into the study | Probably high | Prospective cohort study, prevalent cases at baseline were excluded, but follow-up data was unavailable in one region out of six regions |
| Bias due to a lack of blinding of study personnel | Probably low | Not clear if investigators were blinded but it is not assumed that this is a major bias in this observational study |
| Bias due to exposure misclassification | Probably low | Working hours were self-reported, which seem to be as precise as administrative data |
| Bias in the outcome misclassification | Low | Register-based outcome assessment |
| Confounding | Probably low | Analyses were adjusted to most important confounders (age, and socioeconomic status) |
| Bias due to incomplete outcome data | Low | Register-based outcome assessment |
| Bias due to selective reporting of outcomes | Low | Not identified |
| Bias due to conflict of interest | Low | Not identified |
| Other bias | Low | Not identified |

*Table A5.25 Risk of bias, [Virtanen 2012 - Russek 1958]*

| **Domain** | **Rating** | **Justification for rating** |
| --- | --- | --- |
| Bias in selection of participants into the study | High | Unmatched case-control study, although it is said that the control group were of similar age, occupation and ethnicity |
| Bias due to a lack of blinding of study personnel | Probably low | Investigators were not blinded as the examined their own patients, but it is not assumed that this affected the exposure-outcome association |
| Bias due to exposure misclassification | Probably low | Working hours were self-reported, which seem to be as precise as administrative data |
| Bias in the outcome misclassification | Low | Clinical assessment of IHD |
| Confounding | High | Unmatched case-control study and no adjustments were made |
| Bias due to incomplete outcome data | Low | Incomplete outcome data is not an issue for case-control study |
| Bias due to selective reporting of outcomes | Low | Not identified |
| Bias due to conflict of interest | Low | Not identified |
| Other bias | Low | Not identified |

*Table A5.26 Risk of bias, [Virtanen 2012 - Theorell 1972]*

| **Domain** | **Rating** | **Justification for rating** |
| --- | --- | --- |
| Bias in selection of participants into the study | Probably high | Case-control study, not clear how matched, cannot be confident that cases and controls came from the same population |
| Bias due to a lack of blinding of study personnel | Probably low | Not clear if investigators were blinded but it is not assumed that this is a major bias in this observational study |
| Bias due to exposure misclassification | Probably low | Working hours were self-reported, which seem to be as precise as administrative data |
| Bias in the outcome misclassification | Low | Clinical assessment of IHD |
| Confounding | Probably high | Controls seem not to be matched and analyses were not adjusted |
| Bias due to incomplete outcome data | Low | Incomplete outcome data is not an issue for case-control study |
| Bias due to selective reporting of outcomes | Low | Not identified |
| Bias due to conflict of interest | Low | Not identified |
| Other bias | Low | Not identified |

*Table A5.27 Risk of bias, [Virtanen 2012 - Thiel 1973]*

| **Domain** | **Rating** | **Justification for rating** |
| --- | --- | --- |
| Bias in selection of participants into the study | Probably high | Case-control study, men matched by age only |
| Bias due to a lack of blinding of study personnel | Probably low | Not clear if investigators were blinded but it is not assumed that this is a major bias in this observational study |
| Bias due to exposure misclassification | Probably low | Working hours were self-reported, which seem to be as precise as administrative data |
| Bias in the outcome misclassification | Low | Clinical assessment of IHD |
| Confounding | Probably high | Analyses do not seem to be adjusted for confounders |
| Bias due to incomplete outcome data | Low | Incomplete outcome data is not an issue for case-control study |
| Bias due to selective reporting of outcomes | Low | Not identified |
| Bias due to conflict of interest | Low | Not identified |
| Other bias | Low | Not identified |

*Table A5.28 Risk of bias, [Virtanen 2012 - Falger 1992]*

| **Domain** | **Rating** | **Justification for rating** |
| --- | --- | --- |
| Bias in selection of participants into the study | Probably high | Case-control study, controls were matched by sex (men only), age, and area of residence. |
| Bias due to a lack of blinding of study personnel | Probably low | Not clear if investigators were blinded but it is not assumed that this is a major bias in this observational study |
| Bias due to exposure misclassification | Probably low | Working hours were self-reported, which seem to be as precise as administrative data |
| Bias in the outcome misclassification | Low | Clinical assessment of IHD |
| Confounding | Probably high | Matched and adjusted for age but not for education |
| Bias due to incomplete outcome data | Low | Incomplete outcome data is not an issue for case-control study |
| Bias due to selective reporting of outcomes | Low | Not identified |
| Bias due to conflict of interest | Low | Not identified |
| Other bias | Low | Not identified |

*Table A5.29 Risk of bias, [Virtanen 2012 - Sokejima 1998]*

| **Domain** | **Rating** | **Justification for rating** |
| --- | --- | --- |
| Bias in selection of participants into the study | Probably high | Case-control study in men only, matched by age and occupation |
| Bias due to a lack of blinding of study personnel | Probably low | Not clear if investigators were blinded but it is not assumed that this is a major bias in this observational study |
| Bias due to exposure misclassification | Probably low | Working hours were self-reported, which seem to be as precise as administrative data |
| Bias in the outcome misclassification | Low | Clinical assessment of IHD |
| Confounding | Low | Analyses were adjusted for age and occupation |
| Bias due to incomplete outcome data | Low | Incomplete outcome data is not an issue for case-control study |
| Bias due to selective reporting of outcomes | Low | Not identified |
| Bias due to conflict of interest | Low | Not identified |
| Other bias | Low | Not identified |

*Table A5.30 Risk of bias, [Virtanen 2012 - Liu 2002]*

| **Domain** | **Rating** | **Justification for rating** |
| --- | --- | --- |
| Bias in selection of participants into the study | Probably high | Case-control study matched by sex, age and residence |
| Bias due to a lack of blinding of study personnel | Probably low | Not clear if investigators were blinded but it is not assumed that this is a major bias in this observational study |
| Bias due to exposure misclassification | Probably low | Working hours were self-reported, which seem to be as precise as administrative data |
| Bias in the outcome misclassification | Low | Clinical assessment of IHD |
| Confounding | Probably low | Matched by sex and age, and adjusted for job type |
| Bias due to incomplete outcome data | Low | Incomplete outcome data is not an issue for case-control study |
| Bias due to selective reporting of outcomes | Low | Not identified |
| Bias due to conflict of interest | Low | Not identified |
| Other bias | Low | Not identified |

*Table A5.31 Risk of bias, [Virtanen 2012 - Fukuoka 2005]*

| **Domain** | **Rating** | **Justification for rating** |
| --- | --- | --- |
| Bias in selection of participants into the study | Probably high | Matched case-control by age and sex |
| Bias due to a lack of blinding of study personnel | Probably low | Not clear if investigators were blinded but it is not assumed that this is a major bias in this observational study |
| Bias due to exposure misclassification | Probably low | Working hours were self-reported, which seem to be as precise as administrative data |
| Bias in the outcome misclassification | Low | Clinical assessment of IHD |
| Confounding | Probably high | Matched and adjusted for age and sex, but not for education |
| Bias due to incomplete outcome data | Low | Incomplete outcome data is not an issue for case-control study |
| Bias due to selective reporting of outcomes | Low | Not identified |
| Bias due to conflict of interest | Low | Not identified |
| Other bias | Low | Not identified |

*Table A5.32 Risk of bias, [Jeong 2013]*

| **Domain** | **Rating** | **Justification for rating** |
| --- | --- | --- |
| Bias in selection of participants into the study | Probably high | Case-control study matched by sex, age, type of occupation and region. |
| Bias due to a lack of blinding of study personnel | Probably low | Not clear if investigators were blinded but it is not assumed that this is a major bias in this observational study |
| Bias due to exposure misclassification | Probably low | Working hours were self-reported, which seem to be as precise as administrative data |
| Bias in the outcome misclassification | Low | Clinical assessment of IHD |
| Confounding | Probably low | Matched by sex and age, and adjusted for education |
| Bias due to incomplete outcome data | Low | Incomplete outcome data is not an issue for case-control study |
| Bias due to selective reporting of outcomes | Low | Not identified |
| Bias due to conflict of interest | Low | Not identified |
| Other bias | Low | Not identified |

*Table A5.33Risk of bias, [Cheng 2014]*

| **Domain** | **Rating** | **Justification for rating** |
| --- | --- | --- |
| Bias in selection of participants into the study | Probably high | Matched case-control study by sex (men only), age, education and area of residence |
| Bias due to a lack of blinding of study personnel | Probably low | Not clear if investigators were blinded but it is not assumed that this is a major bias in this observational study |
| Bias due to exposure misclassification | Probably low | Working hours were self-reported, which seem to be as precise as administrative data |
| Bias in the outcome misclassification | Low | Clinical assessment of IHD |
| Confounding | Probably low | Matched and adjusted for age and education |
| Bias due to incomplete outcome data | Low | Incomplete outcome data is not an issue for case-control study |
| Bias due to selective reporting of outcomes | Low | Not identified |
| Bias due to conflict of interest | Low | Not identified |
| Other bias | Low | Not identified |

*Table A5.34 Risk of bias, [Ma 2017]*

| **Domain** | **Rating** | **Justification for rating** |
| --- | --- | --- |
| Bias in selection of participants into the study | Probably high | Case-control study, coronary aniography (CAG) positive were the case and CAG negative were control |
| Bias due to a lack of blinding of study personnel | Probably low | Not clear if investigators were blinded but it is not assumed that this is a major bias in this observational study |
| Bias due to exposure misclassification | Probably low | Working hours were self-reported, which seem to be as precise as administrative data |
| Bias in the outcome misclassification | Low | Clinical assessment of IHD |
| Confounding | Probably low | Analyses were adjusted for age, sex and education |
| Bias due to incomplete outcome data | Low | Incomplete outcome data is not an issue for case-control study |
| Bias due to selective reporting of outcomes | Low | Not identified |
| Bias due to conflict of interest | Low | Not identified |
| Other bias | Low | Not identified |

*Table A5.35 Risk of bias, [McGwin 2005]*

| **Domain** | **Rating** | **Justification for rating** |
| --- | --- | --- |
| Bias in selection of participants into the study | Probably low | Matched case-control study by age, sex, race, time of injury, being nested in a national cohort study |
| Bias due to a lack of blinding of study personnel | Probably low | Not clear if investigators were blinded but it is not assumed that this is a major bias in this observational study |
| Bias due to exposure misclassification | Probably low | Working hours were self-reported, which seem to be as precise as administrative data |
| Bias in the outcome misclassification | Low | Clinical assessment of IHD |
| Confounding | Probably high | Analyses were not adjusted for socioeconomic status |
| Bias due to incomplete outcome data | Low | Incomplete outcome data is not an issue for case-control study |
| Bias due to selective reporting of outcomes | Low | Not identified |
| Bias due to conflict of interest | Low | Not identified |
| Other bias | Low | Not identified |

*Table A5.36 Risk of bias, [Kivimaki 2015 - Holtermann 2010]*

| **Domain** | **Rating** | **Justification for rating** |
| --- | --- | --- |
| Bias in selection of participants into the study | Probably low | Prospective cohort study, male employees in Copenhagen |
| Bias due to a lack of blinding of study personnel | Probably low | Not clear if investigators were blinded but it is not assumed that this is a major bias in this observational study |
| Bias due to exposure misclassification | Probably low | Working hours were self-reported, which seem to be as precise as administrative data |
| Bias in the outcome misclassification | Low | Death certificates with ICD codes |
| Confounding | Probably low | Analyses were adjusted to most important confounders (age, sex, and socioeconomic status) |
| Bias due to incomplete outcome data | Low | Register follow-up on mortality |
| Bias due to selective reporting of outcomes | Low | Not identified |
| Bias due to conflict of interest | Low | Not identified |
| Other bias | Low | Not identified |

*Table A5.37 Risk of bias, [Kivimaki 2015 - O'Reilly 2013]*

| **Domain** | **Rating** | **Justification for rating** |
| --- | --- | --- |
| Bias in selection of participants into the study | Probably low | Prospective cohort study, census-based data |
| Bias due to a lack of blinding of study personnel | Probably low | Not clear if investigators were blinded but it is not assumed that this is a major bias in this observational study |
| Bias due to exposure misclassification | Probably low | Working hours were self-reported, which seem to be as precise as administrative data |
| Bias in the outcome misclassification | Low | Death certificates with ICD codes |
| Confounding | Low | Analyses were adjusted to most important confounders (age, sex, and socioeconomic status) |
| Bias due to incomplete outcome data | Low | Register follow-up on mortality |
| Bias due to selective reporting of outcomes | Low | Not identified |
| Bias due to conflict of interest | Low | Not identified |
| Other bias | Low | Not identified |

*Appendix 6 Exploratory subgroup analyses to determine statistical heterogeneity of studies with pure fatal or non-fatal IHD events and studies with non-fatal and/or fatal IHD events (“mixed”)*

*Fig. A6.1 Exploratory subgroup analysis, Acquired ischaemic heart disease (non-fatal IHD vs. mixed non-fatal/fatal IHD), worked 41-48 hours/week compared with worked 35-40 hours/week, cohort studies*

*
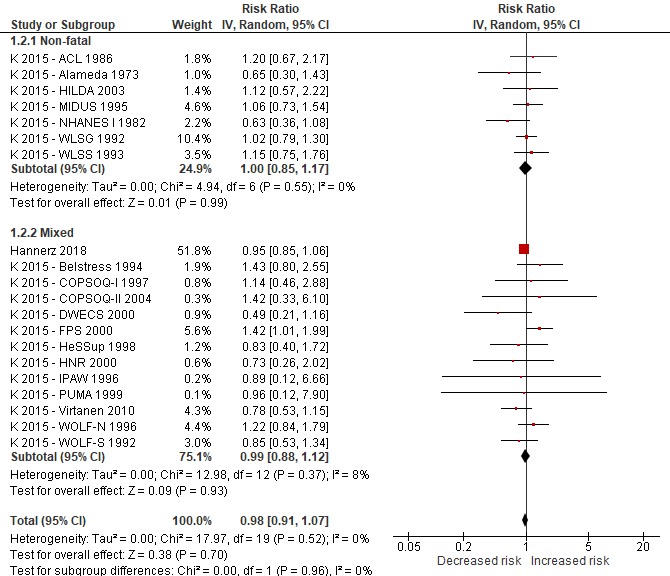
*

*Fig. A6.2 Exploratory subgroup analysis, Acquired ischaemic heart disease (non-fatal IHD vs. mixed non-fatal/fatal IHD), worked 49-54 hours/week compared with worked 35-40 hours/week, cohort studies*


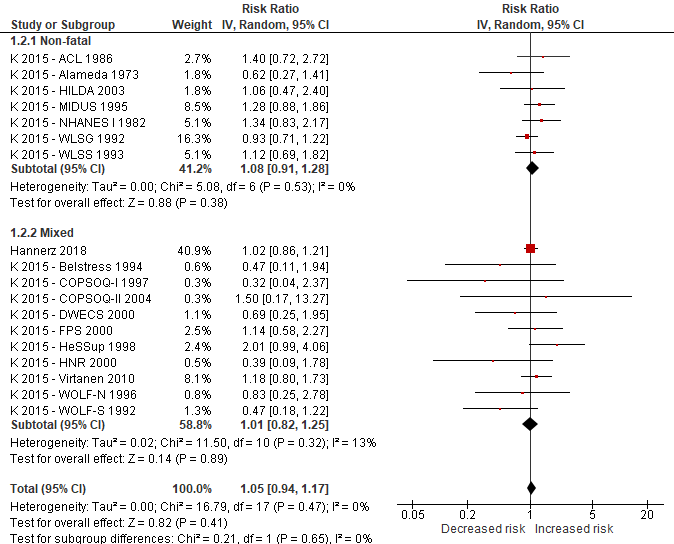


*Fig. A6.3 Exploratory subgroup analysis, Acquired ischaemic heart disease (non-fatal IHD vs. mixed non-fatal/fatal IHD), worked ≥55 hours/week compared with worked 35-40 hours/week, cohort studies*


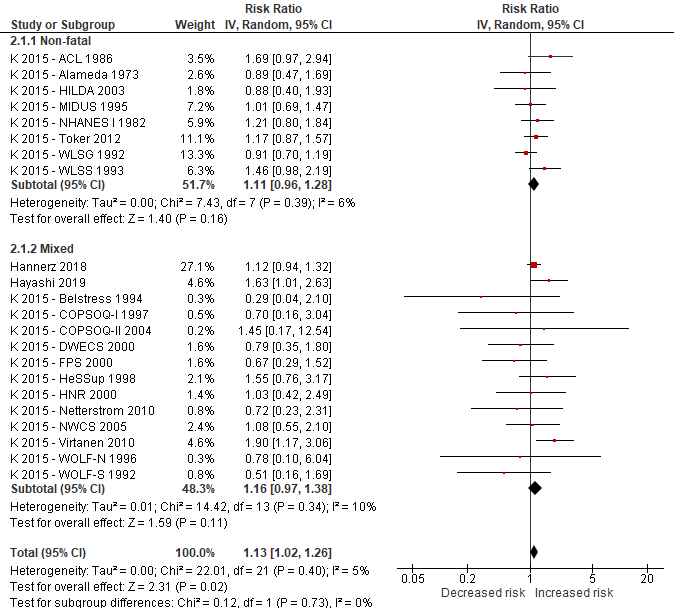


*Fig. A6.4 Exploratory subgroup analysis, Died from ischaemic heart disease (fatal IHD vs. mixed non-fatal/fatal IHD), worked ≥55 hours/week compared with worked 35-40 hours/week, cohort studies*


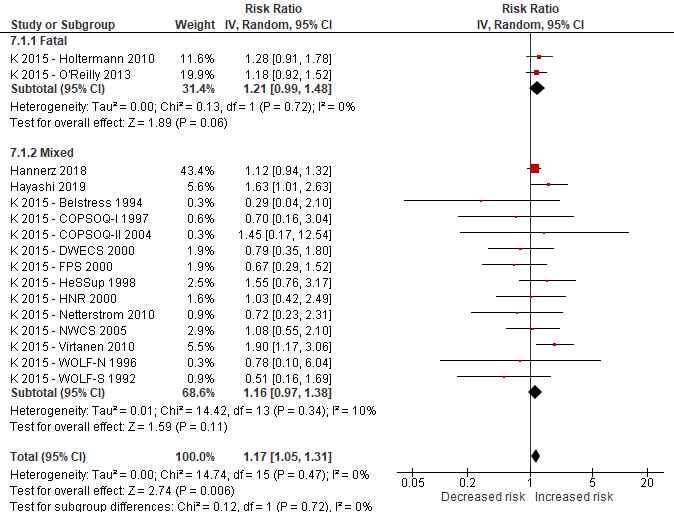


*Appendix 7 Additional subgroup analyses*

A7.1. Has ischaemic heart disease (prevalence of IHD)

The systematic review identified no evidence on this outcome.

A7.2. Acquired ischaemic heart disease (IHD incidence)

A7.2.1. By WHO region

We did not find an obvious difference between the three WHO regions under study (test for subgroup differences p = 0.89) (Fig. A7.1).

Fig. A7.1 Subgroup analysis by WHO region, Acquired ischaemic heart disease, worked ≥55 hours/week compared with worked 35-40 hours/week, cohort studies


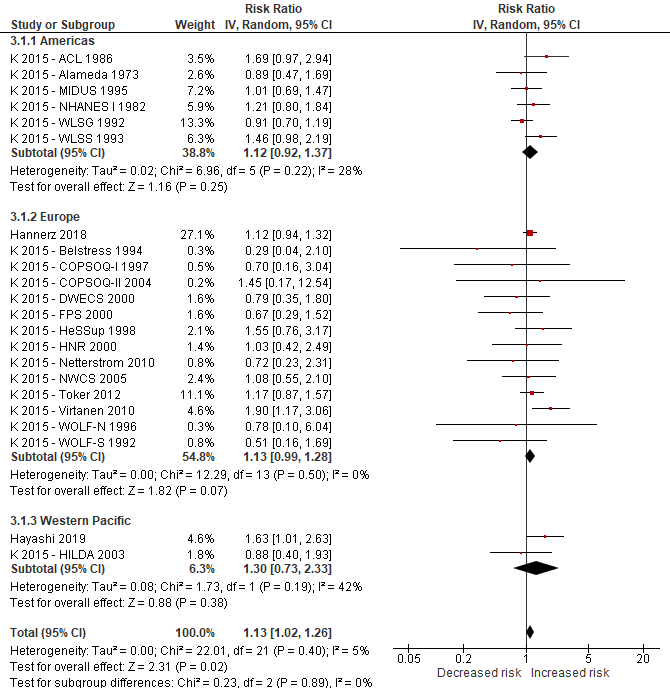


A7.2.2. By sex

There was no evidence for any difference in effect estimates by sex (test for subgroup differences = 0.99) (Fig. A7.2).

Fig. A7.2 Subgroup analysis by sex, Acquired ischaemic heart, worked ≥55 hours/week compared with worked 35-40 hours/week, cohort studies


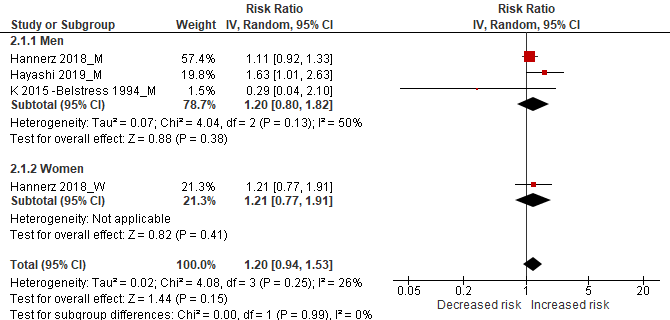


A.7.2.3. By age group

Stratified analysis by age was available by one pooled estimate only. The Kivimaki 2015 meta-analysis of individual-participant data from 20 unpublished studies including non-fatal or “mixed” (non-fatal or fatal) IHD events of an unclear number of participants reported no significant effect modification by age on the risk of IHD of working ≥55 hours/week, compared with working 35-40 hours/week (< 50 years: RR 1.19, 95% CI 0.91 to 1.57; ≥50 years: RR 1.06, 95% CI 0.90 to 1.24; p = 0.50; 20 studies, number of participants not reported, I^2^ not reported).

A7.2.4. By industrial sector

Analyses did not document any differences.

A7.2.5. By occupation

Analyses did not document any differences.

A7.2.6. By formality of economy

Analyses did not document any differences

A7.2.7. By SES

Subgroup analysis according to SES revealed a somewhat stronger effect in the low SES group (test for subgroup differences p = 0.05) (Fig. A7.3).

Fig. A7.3 Subgroup analysis by SES, Acquired ischaemic heart disease, working ≥55 hours/week compared with working. 35-40 hours/week, cohort studies


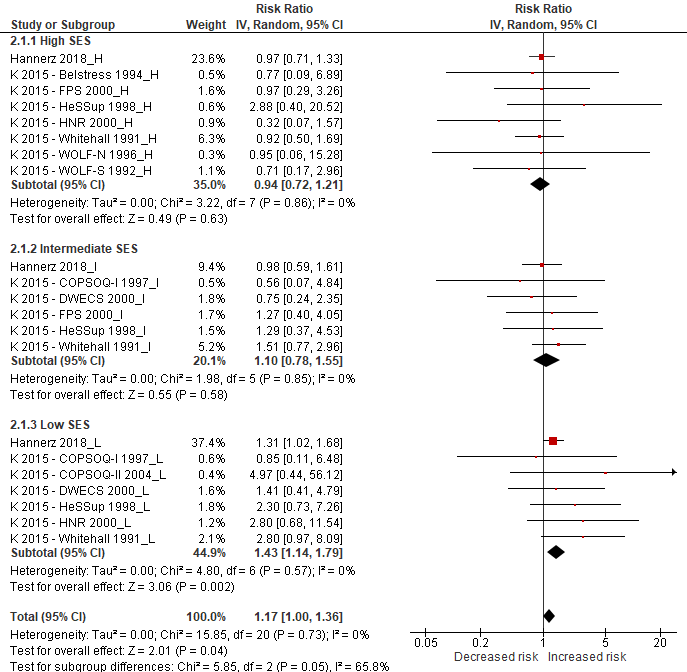


A7.3. Died from ischaemic heart disease (mortality from IHD)

A7.3.1. By WHO region

Again, we did not find an obvious difference between the three WHO regions under study (Fig. A7.4).

Fig. A7.4 Subgroup analysis by WHO region, Died from ischaemic heart disease, worked ≥55 hours/week compared with worked 35-40 hours/week, cohort studies


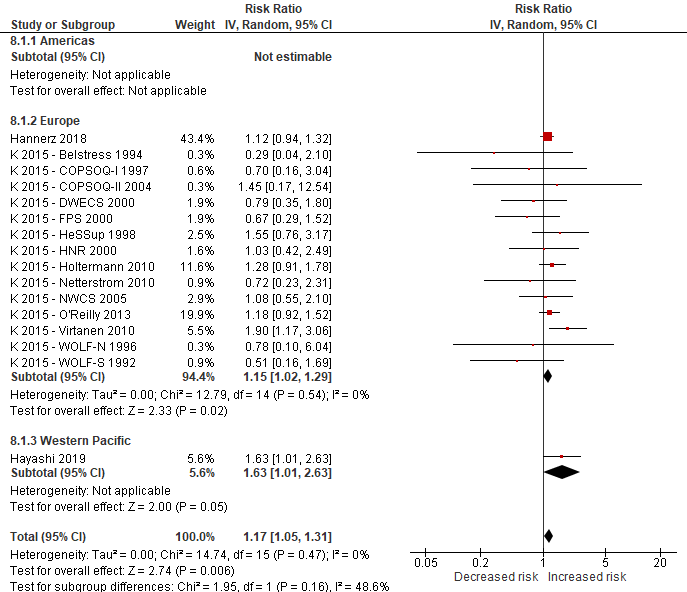


A7.3.2. By sex

There was no evidence for any difference in effect estimates by sex (test for subgroup differences = 0.99) (Fig. A7.5).

Fig. A7.5 Subgroup analysis by sex, Died from ischaemic heart, worked ≥55 hours/week compared with worked 35-40 hours/week, cohort studies


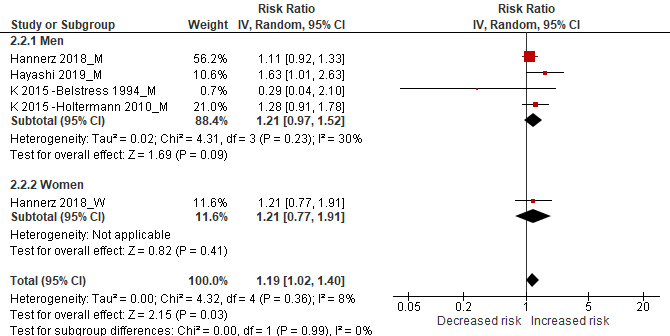


A7.3.3. By age group

Stratified analysis by age was available by one pooled estimate only. The Kivimaki 2015 systematic review and meta-analysis of individual-participant data from 20 unpublished studies including non-fatal or “mixed” (non-fatal or fatal) IHD events of an unclear number of participants reported no significant effect modification by age on the risk of IHD of working ≥55 hours/week, compared with working 35-40 hours/week (< 50 years: RR 1.19, 95% CI 0.91 to 1.57; ≥50 years: RR 1.06, 95% CI 0.90 to 1.24; p = 0.50; 20 studies, number of participants not reported, I^2^ not reported).

A7.3.4. By industrial sector

No studies provided effect estimates disaggregated by industrial sector, and we could therefore not assess differences in effect estimates by industrial sector.

A7.3.5. By occupation

No studies provided effect estimates disaggregated by occupation, and we could therefore not assess differences in effect estimates by occupation.

A7.3.6. By formality of economy

No studies provided effect estimates disaggregated by industrial sector, and we could therefore not assess differences in effect estimates by industrial sector.

A7.3.7. By SES

Again, subgroup analysis according to SES revealed a somewhat stronger effect in the low SES group (test for subgroup differences p = 0.05) (Fig. A7.6).

Fig. A7.6 Subgroup analysis by SES, died from ischaemic heart disease, working ≥ 55 hours/week compared with working. 35-40 hours/week, cohort studies


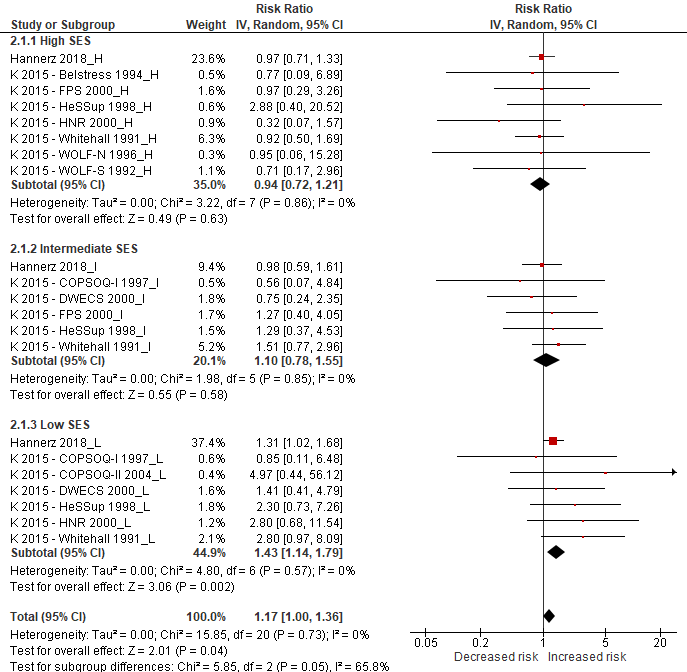


*Appendix 8 Sensitivity analyses*

A8.1. Acquired ischaemic heart disease (IHD incidence)

A8.1.1. Studies with documented or approximated ICD-10 diagnostic codes

There were similar RRs for studies using “health records” and studies with “self-reports” (test for subgroup differences = 0.68) (Fig. A8.1).

Fig. A8.1 Sensitivity analysis, Acquired ischaemic heart disease (“health records” vs. “self-reports”), worked ≥55 hours/week compared with worked 35-40 hours/week, cohort studies


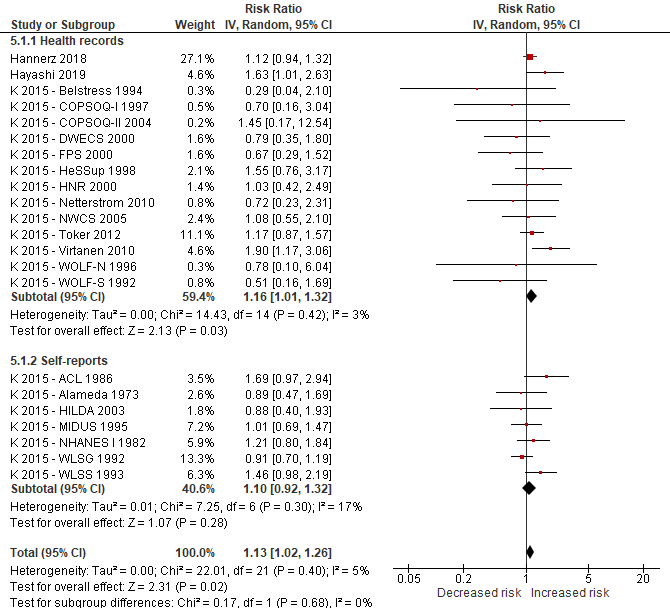


A8.1.2. Studies judged to be of “low” or “probably low” risk of bias

There were no noticeable differences between studies with “low”/“probably low” risk of bias in all RoB domains and studies with at least one rating of “high” or “probably high” in any RoB domains “ (test for subgroup differences = 0.37) (Fig. A8.2).

*Fig. A8.2 Sensitivity analysis, Acquired ischaemic heart disease (“low”/“probably low” risk of bias vs. “high”/“probably high” risk of bias), worked ≥55 hours/week compared with worked 35-40 hours/week, cohort studies*

**
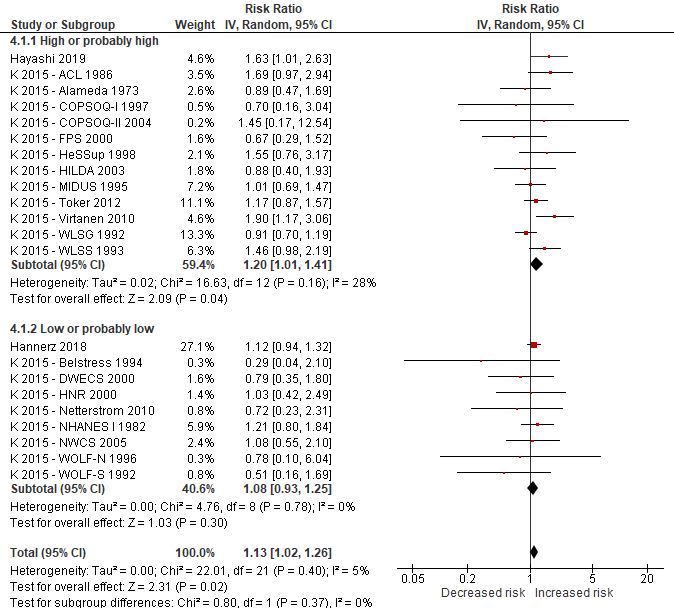
**

A8.2. Died from ischaemic heart disease (mortality from IHD)

A8.2.1. Studies with documented or approximated ICD-10 diagnostic codes

The IHD outcome was based on health records in all studies.

A8.2.2. Studies judged to be of “low” or “probably low” risk of bias

There was no evidence for any difference between studies with “low”/“probably low” RoB ratings across all RoB domains and studies with any “high”/“probably high” RoB rating in at least one RoB domain (test for subgroup differences = 0.13) (Fig. A8.3).

*Fig. A8.3* Sensitivity analysis, Died from ischaemic heart disease (“low”/“probably low” risk of bias vs. “high”/“probably high” risk of bias), worked ≥55 hours/week compared with worked 35-40 hours/week, cohort studies


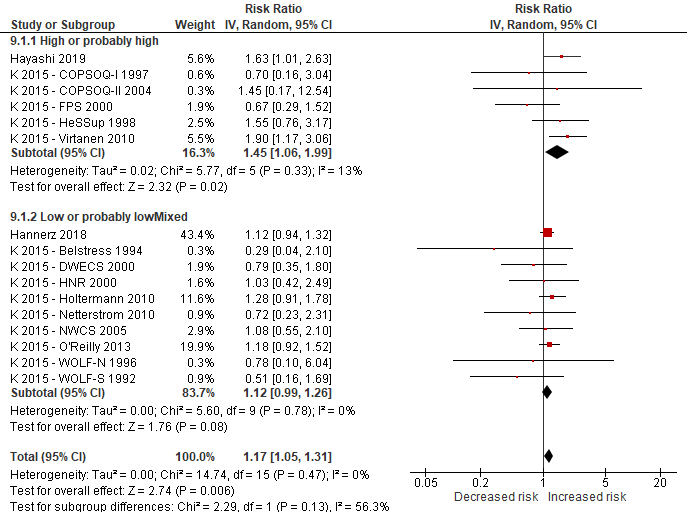


*Appendix 9 Supplementary information on strength of evidence: Bradford Hill criteria*

Given the restriction to observational epidemiologic investigations the standard Navigation Guide methodology ([Lam et al. 2016c](#_ENREF_48)) to rate the strength of the evidence could only partially be applied (see 4.7). The following remarks provide additional information according to relevant criteria from the list proposed by Bradford Hill. Note that this is discussed in the main document and that the approach of evaluating only the human evidence stream is consistent with the GRADE methodology that has adopted the Bradford Hill considerations ([Schunemann et al. 2011](#_ENREF_79)). So, this assessment below mostly overlaps with the more formal analysis in the main text.

**Temporal sequence**: All cohort studies met this criterion as exposure was defined at a time when the study population was either free from outcomes, or when participants with prevalent outcome (IHD) were excluded from the study. Moreover, to reduce bias due to reverse causation, outcome events occurring during the first three years of follow-up were excluded from analysis in the majority of cohort studies ([i.e. in the 20 unpublished studies; Kivimaki et al. 2015](#_ENREF_40)).

**Strength of association**: Overall, the cohort studies revealed a weak strength of associations, with no risk estimate approaching or exceeding the level of 2.0.

**Consistency of associations**: Five out of 20 cohort studies on acquired IHD with a weight of over 60% accounted for an acceptable consistency of findings, despite a relatively large number of non-significant findings. Similarly, among studies analysing risk of dying from IHD, five out of 16 studies with a weight of 86.0% accounted for an acceptable consistency. In both meta-analyses, heterogeneity (I2) was low and test for overall effect was significant.

**Dose-response relationship**: Due to missing data from unpublished cohort studies estimating risk ratios of IHD for the categories 41-48 h/w and 49-54 h/w, our data did not allow a robust test of this causality criterion. However, a pooled analysis of these latter data did not document a dose-response relationship. Second, the assumption of a dose-response relationship between the three exposure categories and the outcome was difficult to determine from our findings. There was no indication of an effect estimate at the lowest exposure categories and perhaps slightly bigger effect at the next lowest exposure category. A positive effect estimate with the lower CI above 1 at the third exposure category, ≥55 hours/week (of 0.98 (0.91, 1.07) (41-48 hours), 1.05 (0.94-1.17) (49-54 hours), 1.13 (1.02-1.26) (>55 hours)). There could be a threshold, but it is difficult to ascertain from the current available evidence.

**Confounding**: Although the link between long working hours and IHD may be influenced, or even mediated, by several behavioural and other work-related factors, and although residual confounding cannot be excluded, all results of the cohort studies entering our meta-analyses were adjusted for the important confounding effects of age, sex and SES. Therefore, this criterion is met at least to a substantial extent.

**Biological plausibility**: To our knowledge, no cohort study exploring the effect of long working hours on IHD has included chemical, physical or biological indicators of pathways that can mediate the observed association, documenting evidence on its biological plausibility. However, with more time spent at work, exposure to different types of toxic substances or conditions is accumulating. Evidence on elevated IHD risks of toxic substances or conditions at work has been demonstrated for noise, shift work, physical activity ([Theorell et al. 2016](#_ENREF_89)), and chronic psychosocial stress at work, as measured by ’job strain’ ([Kivimaki et al. 2012](#_ENREF_41)) or effort-reward imbalance ([Dragano et al. 2017](#_ENREF_11)). There is now growing evidence on chronic activation of stress-physiological pathways among working people exposed to job strain or effort-reward imbalance at work, thus affecting IHD development ([Kivimaki and Steptoe 2018](#_ENREF_42)). Although the cohort studies on long working hours did not include data on these additional health-adverse exposures it is likely that many occupations subjected to long working hours experience one or several of these conditions. Therefore, there is limited support for the notion of biological plausibility of the reported association, mainly due to adverse long-term effects of chronic activation of stress-physiological pathways.
